# Microbotanical residues for the study of early hominin tools

**DOI:** 10.1038/s41598-022-06959-1

**Published:** 2022-02-22

**Authors:** Julio Mercader, George Belev, Pastory Bushozi, Siobhán Clarke, Julien Favreau, Makarius Itambu, Zhu Jianfeng, Samson Koromo, Fergus Larter, Patrick Lee, Jason Maley, Juan Luis Fernández-Marchena, Abdallah Mohamed, Aloyce Mwambwiga, Benja Ngisaruni, Meshack Kingi, Lucas Olesilau, Robert Patalano, Antonella Pedergnana, Ramaswami Sammynaiken, Joakim Siljedal, María Soto, Laura Tucker, Dale Walde, Andreu Ollé

**Affiliations:** 1 Department of Anthropology and Archaeology, University of Calgary, 2500 University Drive NW, Calgary, AB T2N 1N4 Canada; 2 Institut Català de Paleoecologia Humana i Evolució Social (IPHES-CERCA), Zona Educacional 4, Campus Sescelades URV (Edifici W3), 43007 Tarragona, Spain; 3 Department of Geoscience, University of Calgary, 2500 University Drive NW, Calgary, AB T2N 1N4 Canada; 4 Department of Archaeology, Max Planck Institute for the Science of Human History, Kahlaische Strasse 10, 07745 Jena, Germany; 5 Saskatchewan Structural Sciences Centre, Rm. G81 Thorvaldson Building 110 Science Place, Saskatoon, SK S7N 5C9 Canada; 6 Department of Archaeology and Heritage Studies, University of Dar Es Salaam, PO Box 35091, Dar Es Salaam, Tanzania; 7 Department of Anthropology, McMaster University, 100 Main St W, Hamilton, ON L8P 1H6 Canada; 8 Faculty of Arts and Social Sciences, University of Iringa, P.O Box 200, Iringa, Tanzania; 9 Department of Anthropology, University of Toronto, 27 King’s College Circle, Toronto, ON M5S 1A1 Canada; 10 Seminari d’Estudis i Recerques Prehistòriques, Secció de Prehistòria i Arqueologia, Departament d’Història i Arqueologia, Facultat de Geografia i Història, Universitat de Barcelona, c/Montalegre 6-8, 08001 Barcelona, Spain; 11 Natural History Museum, PO Box 2160, Arusha, Tanzania; 12 Ngorongoro Conservation Area Authority, Ngorongoro, P.O. Box 1, Arusha, Tanzania; 13 Institute of Evolutionary Medicine, University of Zurich, Winterthurerstrasse 190, 8057 Zurich, Switzerland; 14 Madrid Institute for Advanced Study (MIAS), Casa de Velázquez, Calle de Paul Guinard, 3, 28040 Madrid, Spain; 15 Departamento de Prehistoria y Arqueología, Facultad de Filosofía y Letras, Universidad Autónoma de Madrid, Ciudad Universitaria de Cantoblanco, 28049 Madrid, Spain; 16 Departament d’Història i Història de l’Art, Universitat Rovira i Virgili, Avinguda de Catalunya 35, 43002 Tarragona, Spain

**Keywords:** Ecology, Evolution, Environmental social sciences

## Abstract

More than 2 million years ago in East Africa, the earliest hominin stone tools evolved amidst changes in resource base, with pounding technology playing a key role in this adaptive process. Olduvai Gorge (now Oldupai) is a famed locality that remains paramount for the study of human evolution, also yielding some of the oldest battering tools in the world. However, direct evidence of the resources processed with these technologies is lacking entirely. One way to obtain this evidence is through the analysis of surviving residues. Yet, linking residues with past processing activities is not simple. In the case of plant exploitation, this link can only be established by assessing site-based reference collections inclusive of both anthropogenic and natural residues as a necessary first step and comparative starting point. In this paper, we assess microbotanical remains from rock clasts sourced at the same quarry utilized by Oldowan hominins at Oldupai Gorge. We mapped this signal and analysed it quantitatively to classify its spatial distribution objectively, extracting proxies for taxonomic identification and further comparison with freestanding soils. In addition, we used blanks to manufacture pounding tools for blind, controlled replication of plant processing. We discovered that stone blanks are in fact environmental reservoirs in which plant remains are trapped by lithobionts, preserved as hardened accretions. Tool use, on the other hand, creates residue clusters; however, their spatial distribution can be discriminated from purely natural assemblages by the georeferencing of residues and statistical analysis of resulting patterns. To conclude, we provide a protocol for best practice and a workflow that has the advantage of overcoming environmental noise, reducing the risk of false positive, delivering a firm understanding of residues as polygenic mixtures, a reliable use of controls, and most importantly, a stronger link between microbotanical remains and stone tool use.

## Introduction

Paleoanthropologists have studied the origins of tool use among human and non-human primates for decades, with pounding technology playing a central role in this quest for its shared legacy of technical gestures with monkeys and apes^1–5^. Percussion-based technology was a key driver during the emergence of the earliest securely dated and understood human technology, the Oldowan, galvanizing human response to shifting environmental conditions and diets more than two and a half million years ago^6–9^. Some researchers have even posited that plant consumption^10–13^ was foundational to the earliest Oldowan toolkits^14,15^, with a later input from meat^16,17^. Unfortunately, direct evidence of plant processing by early Pleistocene hominins is absent, with stone tools at the center of this pursuit, for they allow the extractive behavior at the interface between technology, diet, and the environment.

As tool form alone is not enough to tell function, archaeologists have resorted to ‘trace’ studies^18^ to include mostly macro-wear^19–26^ and, more rarely, micro-wear approaches^9,27–29^. Unfortunately, without direct evidence of the actual materials exploited, ‘trace’ approaches can only outline possible hominin activities; and often yield to equifinality and ambiguity. Studying plant remains as in-situ traces is very challenging as well^30,31^, in that taxonomic ascription is not easy without extracting residues for anatomical inspection. Further, there tends to be a mismatch when comparing archaeological materials with fresh experimental smears for reference^32^. Arguably, however, the greatest handicap to study plant use through stone tool residue is the absence of landscape-level referentials to discriminate environmental noise. Moreover, microbotanical remains from archaeological artifacts are prone to excavation and laboratory contamination^33–35^, a serious concern for tool functional interpretation in ancient phytolith and starch studies^36–41^ that routinely model natural controls to argue for or against the authenticity of putative residues, a practice going back four decades^42^.

**Figure 1:**
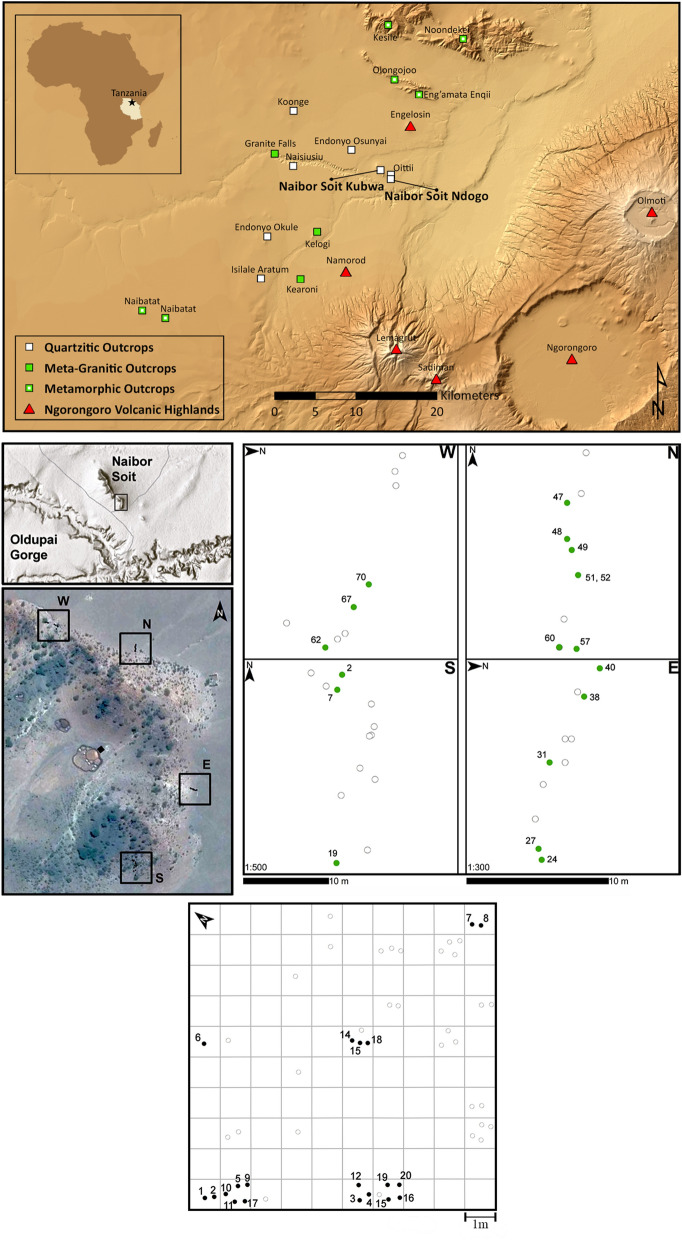
Location map of study area and sampling localities. Top panel: main geological outcrops in the region and location of Naibor Soit Kubwa. Center-left: sampling transects, with cardinal orientation (N, S, E, W), for surface rock collection. The larger image shows the position of the excavated sampling grid in the center (solid black square). Center-right: detailed provenance map within their respective sampling transects (N, S, E, W). Bottom panel: excavation grid showing location of buried blanks.

We fill this gap with the first baseline for the study of plant residues at the most influential and oldest locality for the understanding of prehistoric percussion tools^19,43,44^: Olduvai Gorge (now Oldupai)(Fig. 1), with implications for other sites as well. We assess microbotanical remains on rock clasts from an outcrop known to have been the main source of raw material during the Early Stone Age^45,46^. We mapped this signal, and analysed it quantitatively to classify its spatial distribution objectively^47^, extracting microbotanical proxies for taxonomic identification and systematic comparison with freestanding plant microremains from local soils^35,48^. In addition, we used blanks to manufacture pounding tools during blind, controlled replication of plant processing. Then, we studied these experimental artifacts with the same analytical methods employed for non-anthropogenic blanks. The comparison revealed that rock blanks carry over proxies from the environmental context in which they occurred. Overprinting these natural proxies, human utilization confounds the original ecological signal, adds new markers of technical gesture, and reflects the functional processes that ensued in activity areas. Overall, composition and location of phytogenic palimpsests help discriminate natural from anthropogenic agency in residue accumulation, as well as environmental false positives, thus setting the minimum requirement for future work on plant residues from Oldowan pounding tools.

## Materials and methods

A group of us from the University of Calgary collected materials in northern Tanzania at the quarzitic outcrop called Naibor Soit Kubwa, in 2016. Sampling took place along four transects, with clast location recorded (Fig. 1). One person retrieved every blank, while wearing mask and cleanroom grade gloves, to put it inside a sealed, trace-free plastic bag that remained closed under controlled lab conditions until analysis in 2018:

From the surface, we acquired 28 cobbles and pebbles. (An additional nine surface clasts were collected for experimental archaeology.) We then excavated to a maximum depth of ten centimeters in the outcrop’s topsoil, digging out 28 rock specimens. From each of these two sets, we used 20 stones for microbotanical work and eight for GIS location and distribution analyses. All clasts were characterized (Supplementary Table 1, Supplementary Fig. 1): Roundness was determined following Krumbein’s index^49^, and specimens classified per color, grain size, texture, gloss, transparency, and mineralogy compared to pre-established lithologies within a large reference collection^50,51^ https://www.frdr.ca/repo/handle/doi:10.20383/101.0150. Quartzite blanks have a coarse texture with crystals ≥ 10 mm. Petrographic research ( Supplementary Text) shows secondary overgrowths and bulging recrystallizations, with intra-crystalline fractures infilled by hematite and limonite. Microfissures from the rock surface down are 20–100 µm wide, and phyllosilicate coatings 50–100 µm thick (Supplementary Fig. 2).

We characterized soil composition^52^ by Nuclear Magnetic Resonance (NMR) (Supplementary Method 1), powder X-ray diffraction (P-XRD) (Supplementary Method 2), and Raman spectroscopy (Supplementary Method 3), all showing amorphous silica and aluminum, as well as crystalline silica such as quartz, feldspar, and muscovite, along with aluminosilicate minerals (Extended Data Figs. 1, 2, 3). Molecularly, NMR identified three coordinated aluminum species, and amorphous silica. P-XRD also noted amorphous silica, as suggested by a broad feature 20° to 30°. Raman spectra highlighted crystalline SiO_2_ polymorphs such as quartz, coesite, cristobalite, stishovite, and tridymite, albeit in low frequency.

**Figure 2:**
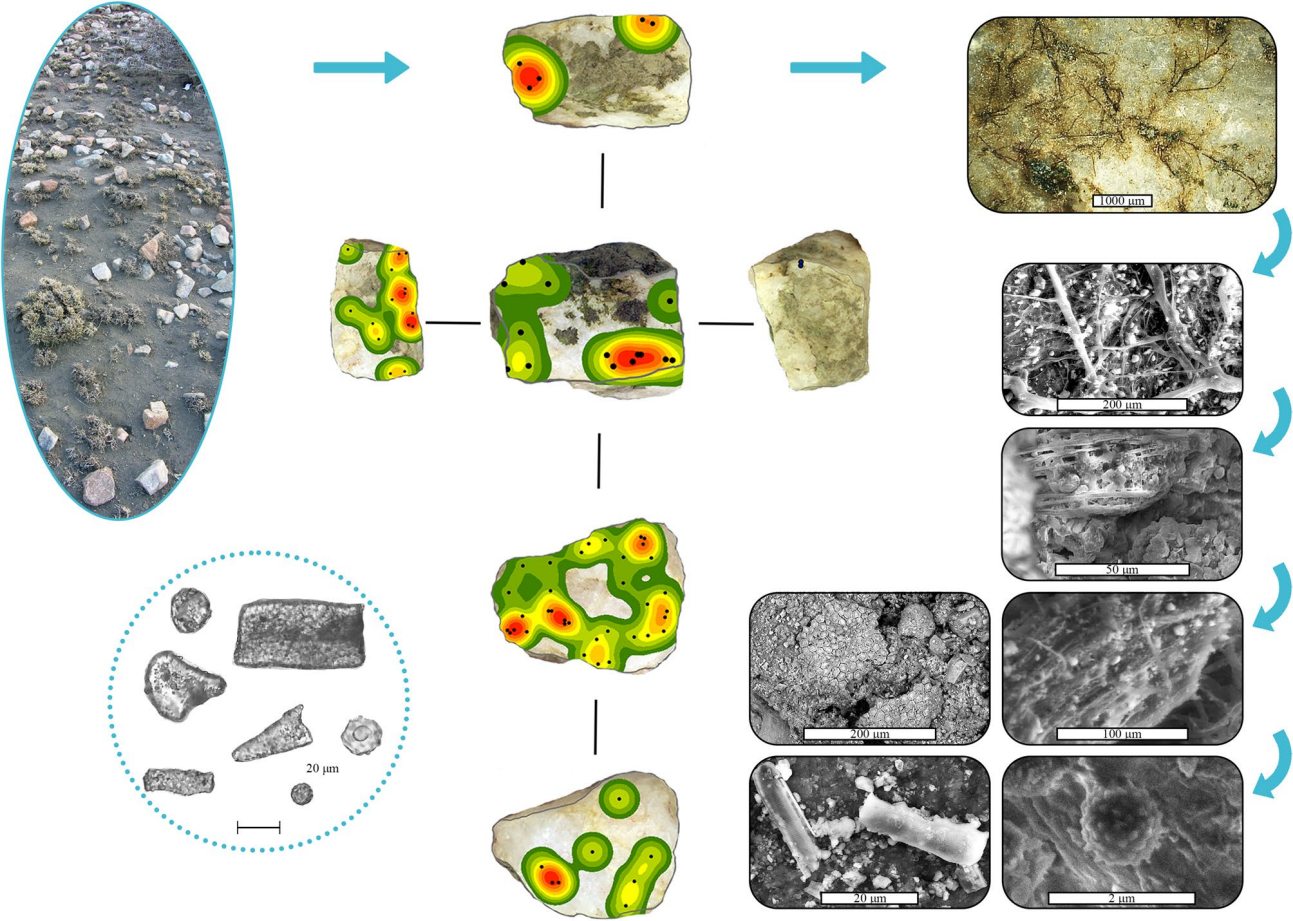
From surface rock to proxy microcosm. Ovate panel, upper left, shows a view of the surface prior to sampling; small bushes for scale. Center images: each subpanel shows a different facet/side of the rock labelled as East 31 (see Fig. 1, center right for provenance). Statistical analysis of georeferenced residue at the mm scale confirms dispersed scatters, in which the areas shown in red hold the highest density, dark green the lowest. Rectangular panels, from top right to bottom right, reveal microcosms from lichens, trapping spores of diverse morphologies, phytoliths, and epidermal tissue. Microlaminations precipitated successively, sandwiching microbotanical remains. Microbial exudates are apparent. Lower left circle shows a compilation of phytoliths, including globular and tabular shapes, together with scutiform and bulliform types.

**Figure 3:**
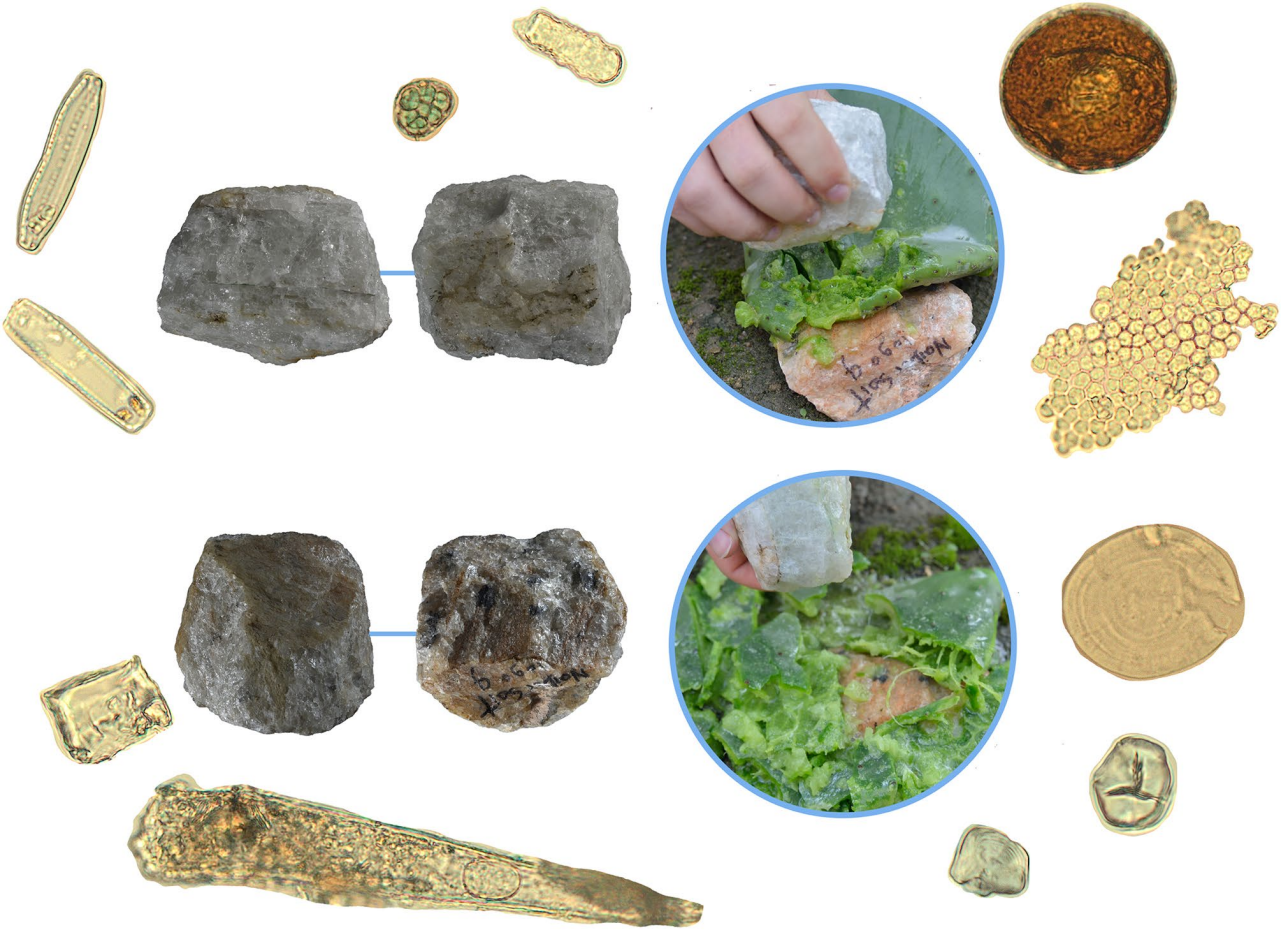
Vignette of microbotanical palimpsest amassed during utilization of Naibor Soit quartzite to smash cactus cladode. Center-left, shows the experimental tool's two main facets, active and passive, while the center right shows images of the pounding process itself. All around these panels, there are microbotanical particles drawn to scale. For reference, the top right spore (dark brown globular) measures ~ 50 microns in diameter. Clockwise, epidermal tissue; similar to other pieces observed in situ (cf. Fig. 2). Known environmental contaminants include Triticeae lenticular starches and orthogonal granules from maize (T-shape centric fissures). Starch granule from cactus pulp. Phytoliths from Oldupai Gorge include large hair and bulliform cells: bottom and corner left. Diatoms, also from Oldupai Gorge, are in the upper left corner. Lastly, in the top center there are a non-pollen palynomorph and a phytolith.

The micro-wear research team from the Catalan Institute of Human Palaeoecology and Social Evolution received surface blanks for lithic experimentation. Stone dimensions and mass were noted (Supplementary Table 2). Lithic modification mimicked the tabular shape that is common in Oldowan ‘anvils’, while pursuing orthogonal reduction for ‘spheroids’^19,44,53^ (Supplementary Fig. 3). All pieces were reduced by free hand, right-handedly, using quartzite pebbles as hammers. Blank reduction stopped when the shape was deemed fit for pounding, not trying to remove the entirety of the cortical surface. After knapping, a subset of these blanks (n = 5) was sonicated (50 kHz) in lab grade acetone for 2 min, to facilitate moulding of the cleaned surface with high precision silicone peels (silicon-based dental impression material: Provil Novo Light—Heraeus Kulzer Inc.) Moulding recorded the experimental tool surfaces prior to use, thus enabling future comparisons of utilized and unused facets. We then engaged in six technical actions (Supplementary Table 3) (Supplementary Video 1–6). The materials pounded included nuts (*Corylus avellana*), underground storage organs (*Solanum tuberosum*), cladode/fruits (*Opuntia ficus*–*indica*), and woody tissue (*Morus alba*), while including animal flesh (*Sus domesticus*) and bone (*Bos taurus*) for comparison (Extended Data Fig. 1). (All animal products utilized for experimentation were purchased at a local meat shop in Tarragona. No live animals were used or sacrificed for research purposes.)

Residue mapping from natural stones was conducted independently by the University of Calgary team; parallel to scanning under a stereo microscope (Olympus SZX12: 7×–90×) for a total recording time of 2390 min (~ 40 h), with average exposure, at any given observation lapse, of eight minutes to avoid contamination. Recording occurred in a cleanroom with filtered air and positive pressure (0.3 lm, HEPA Class H14. Airflow: 26.8 m^3^ min^−1^) (Earth Sciences Building 811). The entire stone surface was inspected along contiguous, but not overlapping transects at low magnification (7×–20×) to locate residues. When these were detected, we shifted to higher power (up to 90×). Further characterization employed 3-D digital microscopy (Hirox KH-8700: 35×–5000× HFOV 8.6–60 μm) and Environmental Scanning Electron Microscopy (FEI Quanta 600: 60×–10,000× HFOV 6.9 mm to 41.4 μm, low-vacuum mode, chamber pressure 0.68 torr 20–30 kV), as well as elemental analysis by Energy Dispersive X-rays (Scios 2). We employed both high-vacuum mode (1x to 5x 10^-5^ mbar) and low-vacuum (15.0 kV) (Supplementary Table 4; Supplementary Method 4).

Quantitative analysis of spatial patterns comprised eight stones per analytical context: surface, topsoil, and experimental (n = 24). These were photographed and georeferenced through ArcMap 10.5.1, overimposing one mm^2^ cells for a Cartesian grid (Supplementary Fig. 4). This image became a polygon shapefile, outlining and measuring rock facets in cm^2^. Kernel density of the shapefile was calculated using a 0.1 cm^2^ search radius, and the shapefile as processing extent (Extended Data Fig. 4). This created a rasterized image of areal scatters per mm^2^. The Nearest Neighbor Index was on the average Euclidean distance between residues as point features. The z-score and p-value of the Nearest Neighbor Ratio nominated accumulation patterns as ‘clustered’, ‘dispersed’ or ‘random’, calculating statistical significance.

**Figure 4:**
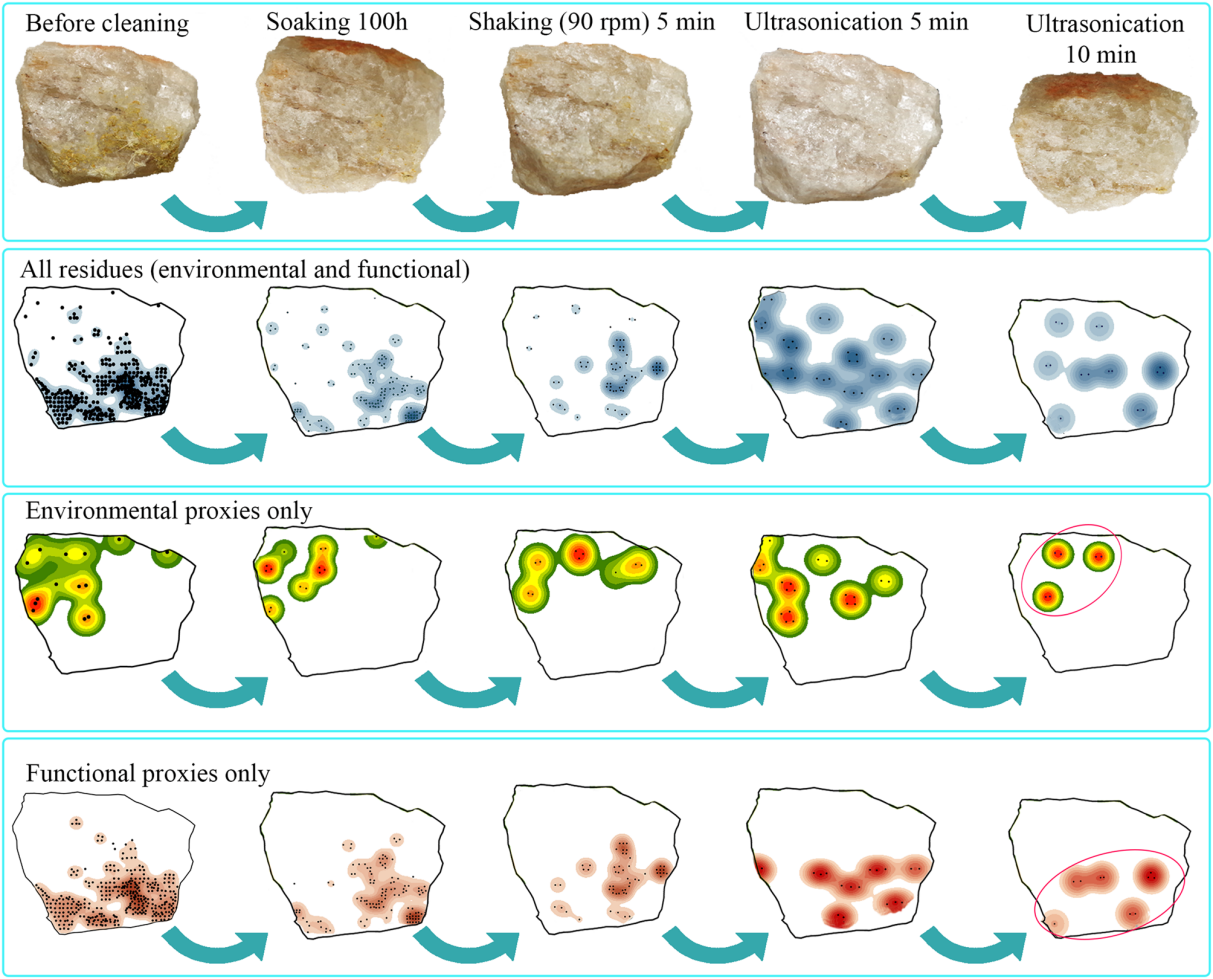
Impact of laboratory cleaning on spatial distribution of residues:natural and anthropogenic**.**

All stone samples were soaked for 100 h in previously boiled reversed osmosis, deionized water in a glass beaker sterilized by autoclaving. The residue thus extracted constituted ‘cycle 1’. Orbital shaking of the stone at 90 rpm followed, separating additional residue as ‘cycle 2’. Ultrasonic cleaning of the stone (40 kHz) ensued for 5 min, with this new set of dislodged residue called cycle 3, followed by another full sonic cycle (no.4).

We restricted our analysis to true coatings, verifiably cemented to the rock specimen. We understand coatings as accreted weathering products forming mixtures that are geological, pedogenetic, and/or biologically mediated, such as skins, hardened matrices, films, lithobiontic mats, accretions, oxides, crusts, and glazes^54^. Microbotanical extractions from cycle 4 ensured exclusion of loose soil; still guaranteeing representability in terms of similar residue distributions and contents, as shown quantitatively (Supplementary Table 5).

Phytolith analysis followed previous work in the study area by the University of Calgary group^35,48^. From a classification perspective, we used the International Code for Phytolith Nomenclature 1.0^55^ to name and describe specimens. As for starch extraction, microscopy, and classification we applied previously published, geographically appropriate procedures for reference materials^56^ with an emphasis on native granules that could be classified based on morphology and metrics. In addition, we tallied diatoms, non-pollen palynomorphs, and sponge spicules, without attempting species-level identification.

## Results

### Blanks as environmental reservoirs

#### Surface blanks

Coating types consists of yellowish brown waxy accretions, reddish brown oxides, white crust, crystallizations, dendritic mats, powdery black masses, and sinuous black concretions (Fig. 2; Extended Data Fig. 5). Elemental chemistry (Extended Data Figs. 6, 7) identified both organic and inorganic substances, iron potassium oxides, as well as organic siliceous mixtures. Different growth types can interdigitate, but blending to create larger clusters is rare, as shown by residue spatial patterns statistically identified as ‘dispersed’. Even though coating varieties are similar in both surface and buried blanks, two qualities are in turn different: areal extent and abundance. On surface rocks, the average coating area is 100 times larger by size, and 22 times greater by percentage of rock coated (0.39 ± 1.99cm^2^/0.89%) (Supplementary Table 6). In contrast, coatings from buried blanks (Supplementary Table 7) spread just over an average of 0.0037 ± 0.0075 cm^2^/0.040%.

**Figure 5:**
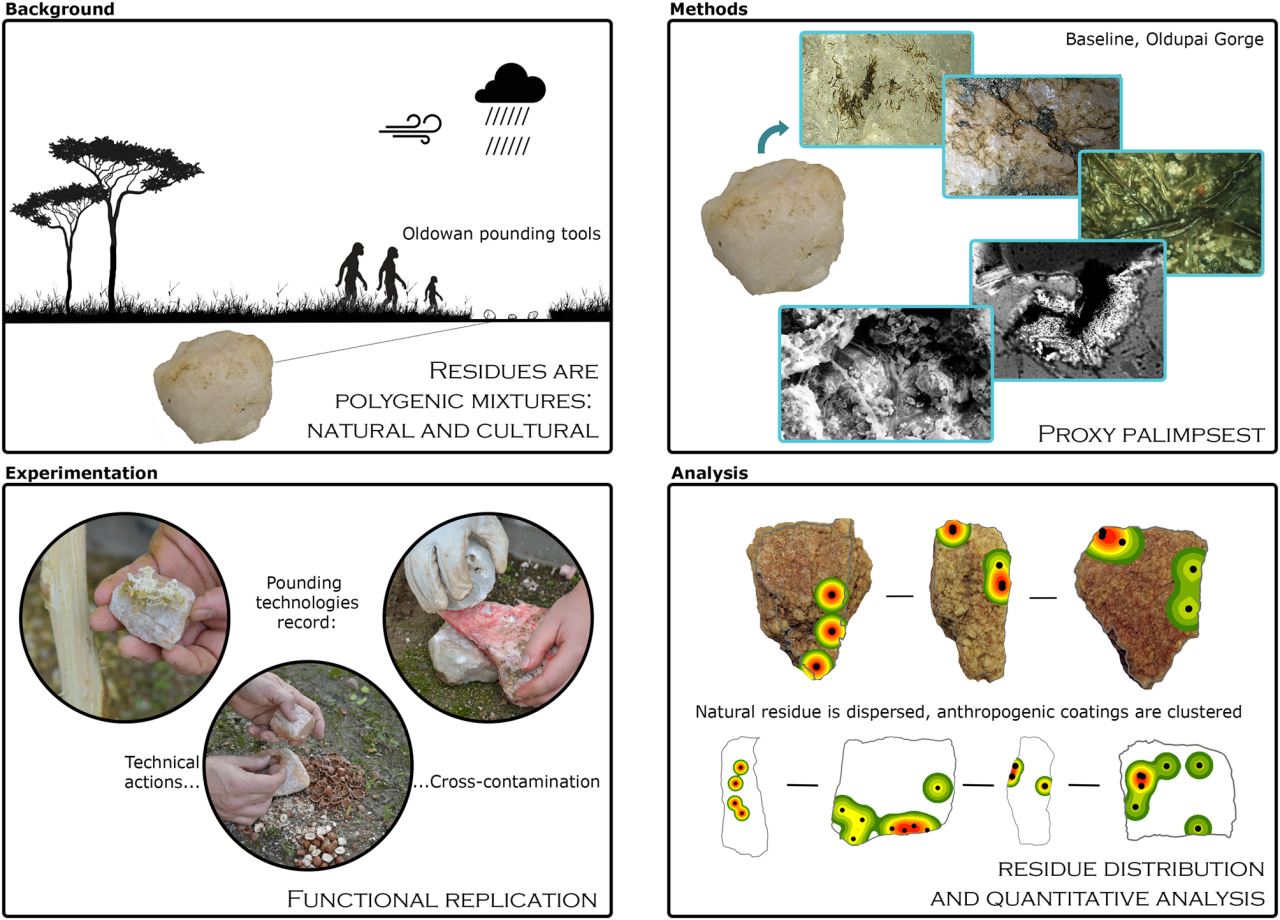
Graphic summary of findings.

**Figure 6:**
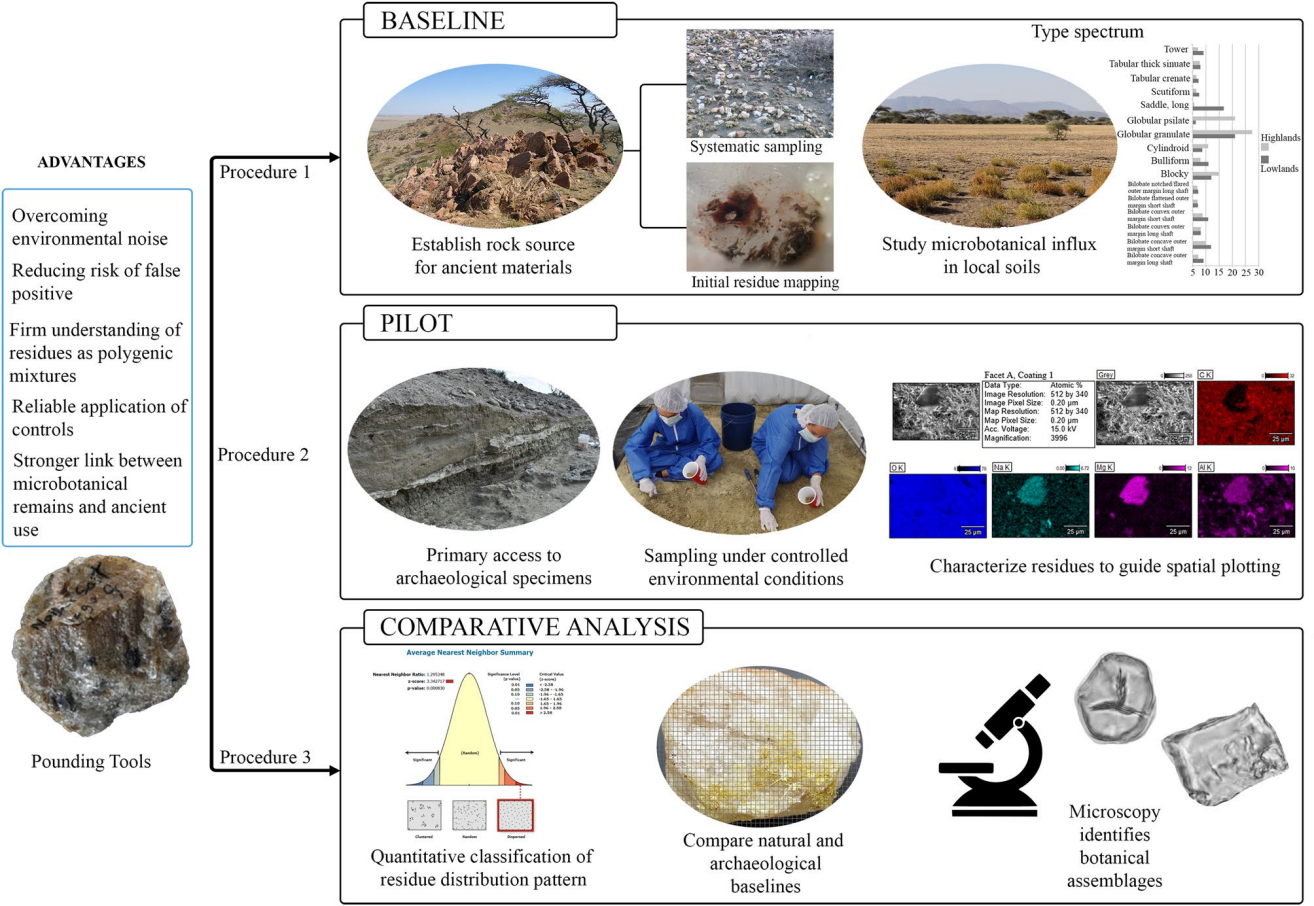
Protocol outline for the study of Oldowan pounding tools showing advantages, and the various steps during the construction of a baseline, pilot testing, and comparative analysis. All human images are of authors.

The habitability of aerial and subaerial rock surfaces for lichens created a microcosm in which dendritic thali are the foundation for successive accretion cycles, trapping spores, phytoliths, diatoms, and starch granules; all cemented by coalescence of microbial exudates, and geogenic and soil precipitates (Extended Data Fig. 8). This explains the large microbotanical assemblages recovered (Extended Data Fig. 9), in spite of soaking, shaking, and sonic cleaning (n = 536), inclusive of phytoliths (n = 278), starch granules (n = 174), diatoms (n = 55), palynomorphs (n = 26), and sponge spicules (n = 3) (Supplementary Table 8, 9, 10). Microbotanical populations have non-normal distributions. Morphotypes are 20 for phytoliths (5 types represent ¾ of the assemblage) and eight for starch granules (3 account for ¾). The number of studied blank facets averages six per stone, and up to 48 facets in total. The average area per facet is 41.74 ± 21.74 cm^2^. Of 48 facets, 32 accumulated residue, mostly ‘dispersed’, but sometimes ‘random’, even ‘clustered’ in one case (Supplementary Table 11). Statistical correlation is lacking for a series of variables: stone area/coating area, facet area/coating areal extent, and stone area/microbotanical remains (Supplementary Table 12).

#### Subsurface blanks

Elemental analysis highlights organic matrices engulfing sodium/potassium/aluminum silicates (Extended Data Figs. 5, 10, 11), matching the ion-rich nature of local soils^57^. Other crusts resemble the organic/silico-calcareous mixtures observed in surface coatings. Biogenic fibrous strands are rich in sulfur, phosphorus, and potassium (Extended Data Figs. 5, 10). Siliceous and organic coatings both trap biogenic clasts. Microbotanical populations are large (n = 560; phytoliths = 307; starches = 229; diatoms = 6; palynomorphs = 17, sponge spicules = 1) (Extended Data Fig. 12; Supplementary Table 8, 9, 10), exhibiting non-normal distributions. Phytolith morphologic diversity is at 18 types dominated by the same five common contributors seen on surface blanks. The starch granules exhibit seven morphotypes, dominated by one class. The mean number of facets per blank is six, and up to 48 in total. Facet area averages 23.15 ± 17.81 cm^2^. From 48 facets, only 13 had enough residue visible at low magnification for georeferencing: scatters are mostly ‘dispersed’ with one instance of ‘random’ deposit (Supplementary Table 11). We could not find statistical correlation for the following variables: stone area/coating area, facet area/coating extent, and stone area/microbotanical remains (Supplementary Table 12).

### Utilization creates residue clusters

Experimental tools yield the same coating types detected in natural blanks plus an additional six categories: spinous, cellulosic, vascular, fibrous, filamentous, and pale waxy crust (Fig. 3). Residues spread over an average of 1.61 ± 2.96 cm^2^/3.59% (Supplementary Table 13), noticing that anvils keep twice as much as spheroids. The patterning is demonstrably different to that detected in natural coatings, with an overwhelming dominance of ‘clustered’ remains, and a slight representation from ‘random’ and ‘dispersed’ scatters. Microbotanical particles contribute the largest dataset (n = 936), led by diatoms (n = 417) and palynomorphs (n = 350), followed by starch granules (n = 129) and phytoliths (n = 40). Phytolith morphotypes total 15 and starches seven; those already observed for natural clasts, plus an additional six types from experimental utilization and contaminants (Extended Data Figs. 13, 14), amounting to a subtotal of 107 granules (Supplementary Table 14). All blanks reached a combined facet number of 98, with average area of 50.91 ± 31.16 cm^2^ per facet. One subset of four spheroids totalled 50 facets. Of these, 25 supported ‘clustered’ coatings, while six are ‘random’ and three ‘dispersed’. Sixteen spheroid facets have no residue. The other subset comprised anvils, totalling 48 facets, with 32 showing ‘clustered’ residues, three ‘random’, and one ‘dispersed’. Twelve anvil facets were void of residue. Microremain populations are non-normally distributed, with correlation absent for stone area/coating area, facet area/coating area, and stone area/microbotany (Supplementary Table 12).

The environmental signal on experimental tools, inherited from Oldupai Gorge and made up of diatoms, palynomorphs, phytoliths, and starch granules, now sees added proxies from technical and experimental processing actions, as well as cross contamination, confounding the original fingerprint to create a statistically different assemblage from both non-anthropogenic rocks and freestanding soils (Supplementary Table 15). These new indicators include starch granules from published contaminants in starch research, such as maize and wheat^33^, along with the starches themselves employed for the experiment: spheroids, as active pounding components, retained starches from the cactus fruit, but we could not retrieve hazelnut starch from the spheroid that cracked open hazelnuts. Cross contamination from experimental and environmental starch granules is apparent in all spheroids, including those used to deal with animal tissue. Regarding anvils, we could not retrieve cactus starches from the stone piece dedicated to cactus processing, while the anvils involving potato and hazelnut did produce their respective starches and cross contaminants.

### Anthropogenic residue distribution

#### Active stones (spheroids)

Two experimental tasks required an active stone: nut cracking and smashing of succulents. Nut cracking cemented residue on one facet, where the resulting accumulation was large in size but ‘dispersed’ in distribution. Cladode smashing, however, coated all facets with ‘clustered’ accumulations, of which two facets accrued residue profusely (Fig. 3). The spheroid utilized for this task was, in fact, the only specimen in which the active facet, as per the Catalan Institute of Human Palaeoecology and Social Evolution’s records (Supplementary Fig. 5) had the largest residue scatter from a purely quantitative point of view, as per analysis by the University of Calgary team (Supplementary Fig. 6). In all other pieces, the classification of a ‘lead facet’ by Nearest Neighbor Ratio ended up misaligned with the actual active side recorded at the time of use, whose residue consistently scored a lesser areal distribution. We also processed animal tissue for comparison both flesh and bone: for meat pounding, the active stone sheltered residue on two facets, while bone cracking assembled very large ‘clustered’ coatings in all facets and up to three ‘lead’ sides.

#### Passive stones (anvils)

The anvil for nut cracking did not coat three of its facets, while creating two ‘clustered’ facets and a random one. The clustering responds to the two processing actions undertaken, shelling and grinding, each with a different side. Smashing cactus coated all anvil facets with large ‘clustered’ build-up (Fig. 3). Using one anvil to scrape potato generated large ‘clustered’ coatings in all facets. Lastly, the anvil employed for bone cracking clustered coatings in all sides (Supplementary Fig. 7).

#### Scraping stone

A polyhedral piece was utilized to scrape woody tissue off white mulberry (Moraceae: *Morus alba*), using one ridge during 15 min as the active side and scraping at 90 degrees transversally and bidirectionally. We subsequently used this piece as a model specimen to investigate the impact of cleaning protocols on residue spatial arrangement, and discriminate natural from functional residue (Fig. 4). Plotting occurred sequentially: (i) prior to any treatment, (ii) after soaking, (iii) upon orbital shaking in water, (iv) after one sonication cycle, and (v) after a second sonication. The stone facets studied were six, with area of 99.91 cm^2^. Residue dropped from 8.02 cm^2^ (prior to treatment: 8%) to 1.09 cm^2^ (after soaking 100 h), 0.4 cm^2^ (after orbital shaker), 0.18 cm^2^ (after sonication no. 1), and finally to 0.08 cm^2^ after a second sonication treatment. However, the ‘clustered’ accumulation on the utilized edge remained unchanged throughout the cleaning process, showing similar location and microbotanical characteristics (Supplementary Table 5). Quantification of residue distribution discriminated natural pre-existing residue from anthropogenic matter on the grounds of position, scatter pattern, and microremain type.

## Discussion

Phytogenic materials accumulating on natural rocks are many and diverse: They include phytoliths, starch granules, diatoms, sponge spicules, and non-pollen palynomorphs. Currently, it remains difficult to ascertain clear links between microbotanical remains extracted from artifacts and ancient use because the chain of evidence to discriminate function-related from natural residues pends on a weak link, and hence establishing whether residues detected on stone tools have to do with prehistoric use is challenging^57,58^. Our study is the first baseline of natural coatings from rock samples across the landscape of Oldupai Gorge, a World Heritage Site. We studied a large set of natural rocks rather than archaeological lithics to eliminate human intervention as cause for any residue pattern or microbotanical accumulation, other than those from replication and experimental utilization. Considering the ambitious and time-consuming endeavor of this initial task, a systematic comparison of residues with use-wear analysis on spheroids and anvils is beyond the scope of this article. Our only goal at this time is to outline protocols for Oldowan tools, as dictated by evidence from natural stone counterparts. This referential serves to remove the uncontrollable introduction of environmental signals when discriminating function/palaeodiet from ecological context. Failure to account for these will inevitably result in flawed data and misinterpretation of both natural controls and archaeological cases. Our dataset is also the largest assemblage of microbotanical remains explicitly generated to guide archaeologists in the interpretation of environmental noise in Early Stone Age pounding tools.

### Of lichen habitability, proxy palimpsests, and hardened accretions

For a modern, successful approach to plant residue analysis, it is crucial to know the origin and provenance of phytogenic residues on natural blanks, surficial and buried, as a comparative starting point. The chemistry of local soils facilitated attachment of biofilms to rock surfaces, contributing intra-crystalline residues and endolithic penetration of blanks. Several features in the studied assemblage evidenced micro-discontinuities and quartzite alteration suggesting open and changing systems that make residue accretion and biofilm modulation possible^59–61^: Oldupai’s quarzitic rocks have abundant plane fractures, voids, and microfissures (Extended Data Fig. 8); often infilled by silicates^62^. This is hardly surprising, considering their high surface area, hydrophobic/hydrophilic surfaces, ionic exchange properties, as well as the fast development of silica/aluminum glazes on rock surfaces^54,63,64^. Aligned with this data is our suggestion that bioclastics were blown onto the rock surface, mixing during rainfall events with amorphous silica, aluminum, and calcium, so that the drying of poorly ordered mineral phases created a hardened deposit on stones as dispersed residue. This layer partly comes apart when buried (smaller coatings overall were documented in subsurface rock samples), and when soaked, shaken, and sonicated.

Our work proves the circular nature and weakness in the old assumption that control rocks and sediments should harbor no microbotanical materials, or just contain minor noise relative to utilized tools^42^. This hypothesis would hold if both target and control came from the same population. To check this environmental transfer hypothesis, we tested both degree of association and population distribution in microbotanical particles. First, only ¼ of surface and buried stones respectively are void of phytoliths or starch granules, not all of them. More importantly, microbotanical remains from surface and buried rocks are not associated; thus, they are different from one another, coming from populations with equal distributions (Supplementary Table 16). The same quantitative outcome derives from comparing phytolith and starch populations from surface and subsurface rocks both at the assemblage level and by morphotype, and the comparison with phytolith and starch populations from freestanding soils (Supplementary Table 15, 16).

### A protocol to study plant residue from Oldowan pounding tools

The analysis of macro-wear from modern hammers and anvils among non-human primates to infer Oldowan tool function has become increasingly popular^20,21,23,25^. Inspired by these efforts, various researchers have focused on qualitative and (semi) quantitative analysis of wear proxies^47^, such as stone polish, damage location, and surface roughness. While the potential of centimetre-scale use-wear modifications on percussion tools is an evident first step, its applicability to study Oldowan plant use is limited, as it cannot isolate the residues needed to  secure direct evidence and identify ancient extractive behavior. Therefore, there is an emergent need to establish workflows optimized for the recovery of residue from stone tools. However, these methods must overcome the inevitable noise from false positives, as researchers often link incidental coatings to usage without heavy proof, not recognizing that residues are polygenic mixtures^57,58^. Moreover, the exact location of these residues through geo-referencing needs visualization and amenability to quantitative analysis.

We geo-referenced residue on stone at the mm scale^47^. This was the most accurate way to characterize location, nature, and size, allowing quantitative analysis to classify distribution objectively. We demonstrated that total blank volume and facet area had no impact on the abundance of either coatings or phytogenic populations, all of which are distributed non-normally. Surface and buried rocks support dispersed residue scatters. One important difference between natural coatings and use-related residue is its clustered geography, not only immediately after use, but also after removing residues aggressively by soaking, shaking, and ultrasonication^65^. Another finding is that the area occupied by residues on surface materials is much larger than that on buried rocks, which probably shows the impact of burial on coatings. In short, the location of anthropogenic residue on used blanks, its spatial concentration, and their microbotany are a mixture of technical gestures and the various materials that existed within the activity area, as shown by recurrent cross contamination during experimentation. The coatings from experimental tools were also ubiquitous; with most facets covered by residues, whether they were active sides or passive ones^29,66,67^, even when accounting for the use of plants with variable water contents. In fact, anthropogenic residues do not necessarily associate with functional edges or use-wear (Extended Data Figs. 6, 7)^32,65,66^, or at least not those resulting from the pounding of fresh materials.

In closing, we provide a graphic summary of our findings (Fig. 5) and a protocol (Fig. 6), with recommendations for best practice, noting the following workflow: first, establish a baseline by collecting raw materials from the study area that mirror those present in the target archaeological collections. Second, clean up natural specimens under controlled, reproducible conditions, and plot residues accurately after each cleaning step. Third, generate an inventory of natural coatings, along with their associated microbotanical populations. Fourth, georeference residue patterns on both natural and archaeological specimens. Fifth, compare natural and archaeological scatters to estimate, if possible, natural inheritance versus technical gestures and use. This methodological approach has the advantage of overcoming environmental noise as a confuser, reducing the risk of false positive, delivering a firm understanding of residues as polygenic mixtures, a reliable use of controls, and most importantly, a stronger link between microbotanical remains and ancient stone tool use.

## Supplementary Information

Supplementary Video 1.

Supplementary Video 2.

Supplementary Video 3.

Supplementary Video 4.

Supplementary Video 5.

Supplementary Video 6.

Supplementary Information.

Supplementary Legends.
