# Supplementary material for: Microbotanical residues for the study of early hominin tools
Source: Sci Rep. 2022 Feb 22;12:2951. doi: 10.1038/s41598-022-06959-1 (PMC8863820; doi:10.1038/s41598-022-06959-1)
Supplement: Supplementary file 7 — Supplementary Information. [file 41598_2022_6959_MOESM7_ESM.pdf]

Extended Data Figure 1. Solid State NMR spectroscopy measurements of samples no. 131, 191, and 260 (See citation [45] for provenance details). All the spectra have three bands at around 5, 30, and 60 ppm, corresponding to three different coordinated Al species - AlO_6, AlO_5, and AlO_4, respectively. The relative intensities of the Al signals are different across the three samples. In addition, the ^{27}Al signals are broader in MPI-260 than the other two samples, due probably to that the MPI-260 sample is more amorphous. The other peaks in the spectra are spinning side bands. The ^{29}Si MAS spectrum of MPI-191 has three bands at around -94, -103, and -112 ppm, corresponding to three different coordinated Si species – Q^2, Q^3, and Q^4, respectively. The symbol Q^n denotes Si connected to n SiO_4 units – $Si(SiO_4)_n(X)_{4-n}$, where X = OH or AlO_4. The ^{29}Si CP/MAS spectrum of MPI-191 also has the same three bands, but the intensity of Q^4 is much weaker than that in the MAS spectrum. This is reasonable since the CP signal is transferred from ^1H to ^{29}Si via dipolar coupling and there is no OH group directly bonded to Si in Q^4 unit. ^{29}Si MAS spectra of the MPI samples are very similar (Figure 2) and are similar to those reported for amorphous silica.[1] The ^{29}Si CP/MAS spectra of the three samples have the same bands, but with different relative intensities.

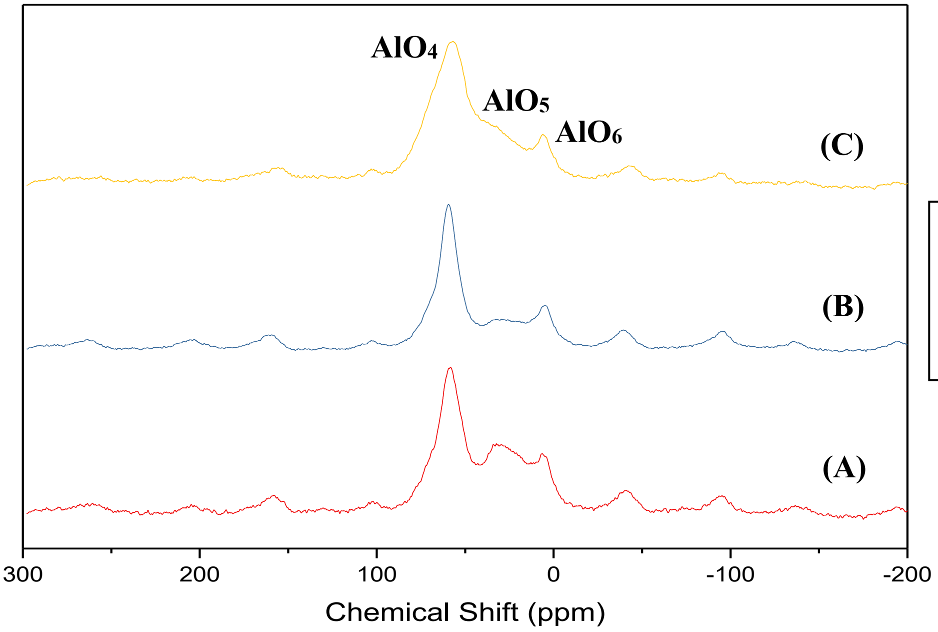

^{27}Al MAS NMR spectra of (A) MPI-131, (B) MPI-191, and (C) MPI-260

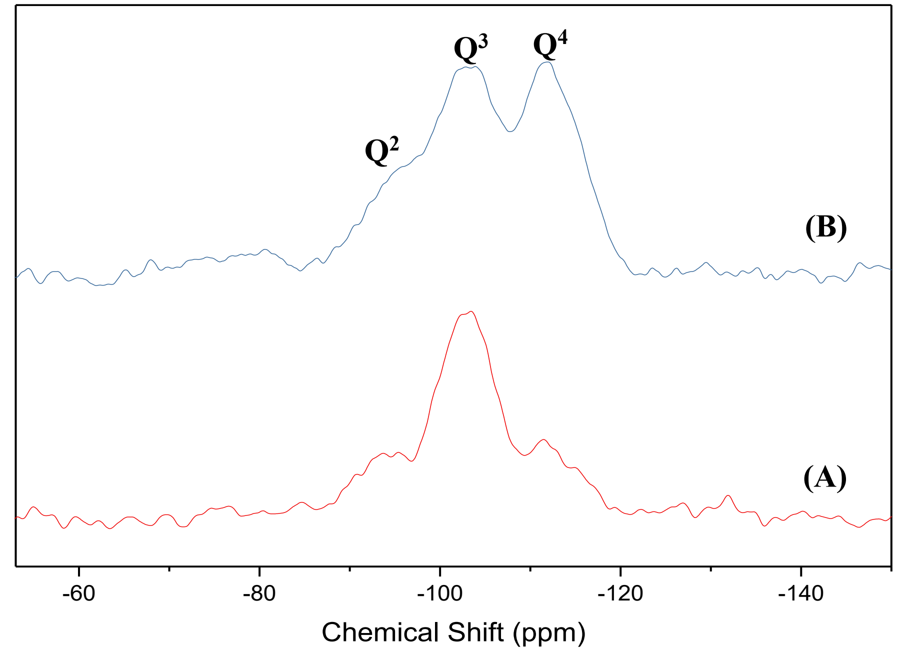

^{29}Si (A) CP/MAS and (B) MAS NMR spectra of MPI-191.

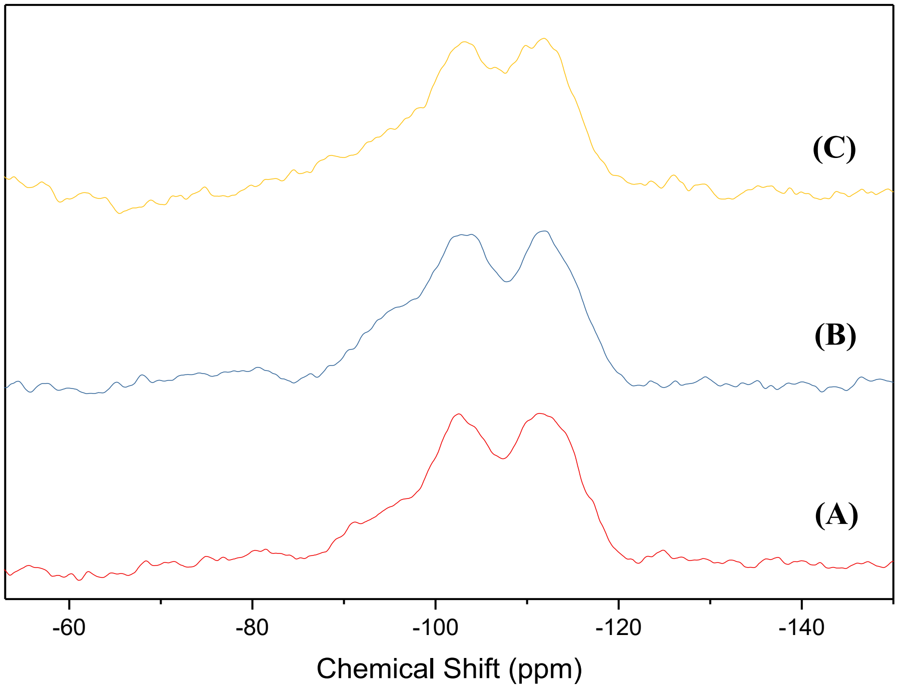

^{29}Si MAS NMR spectra of (A) MPI-131, (B) MPI-191, and (C) MPI-260.

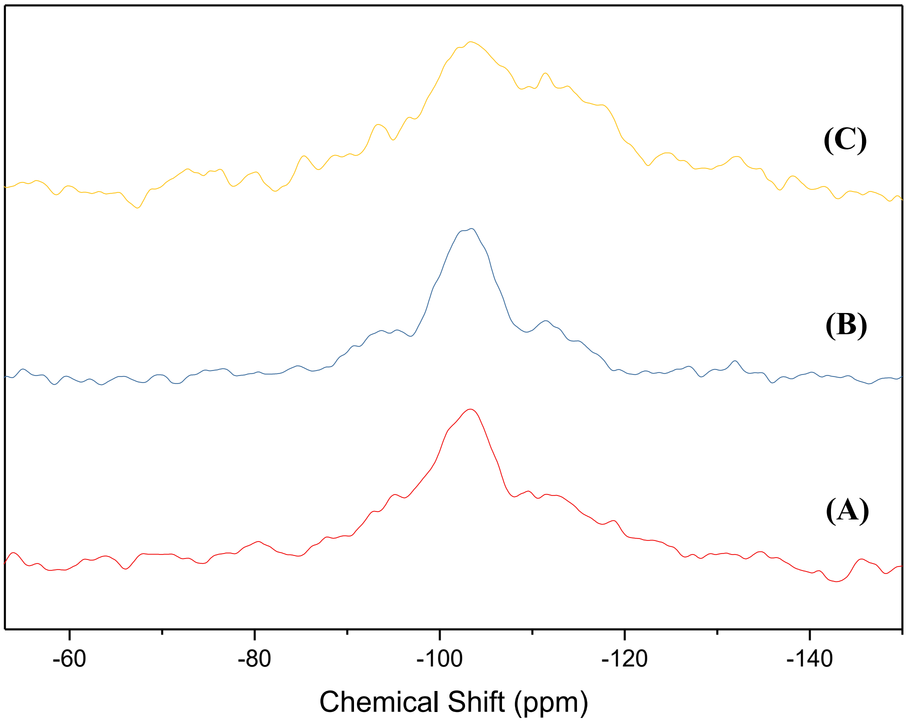

^{29}Si CP/MAS NMR spectra of (A) MPI-131, (B) MPI-191, and (C) MPI-260

Extended Data Figure 2. Powder X-Ray Diffraction of samples no. 131, 191, 260 (See citation [45] for provenance details). The samples are expected to have SiO_2 and silicate phases. The amorphous phase is likely amorphous silica, also suggested by the ^{29}Si solid state NMR results. XRD patterns of possible crystalline SiO_2 and silicate phases (data from the RRUFF database) are: 1) peak at 8.82°, corresponding to a d space of 10.02 Å, in MPI-191 for muscovite, 2) peak at 26.6° in MPI-191 for quartz phase, and 3) peak at 27.6° in MPI-131 and MPI-191 for orthoclase or microcline phase.

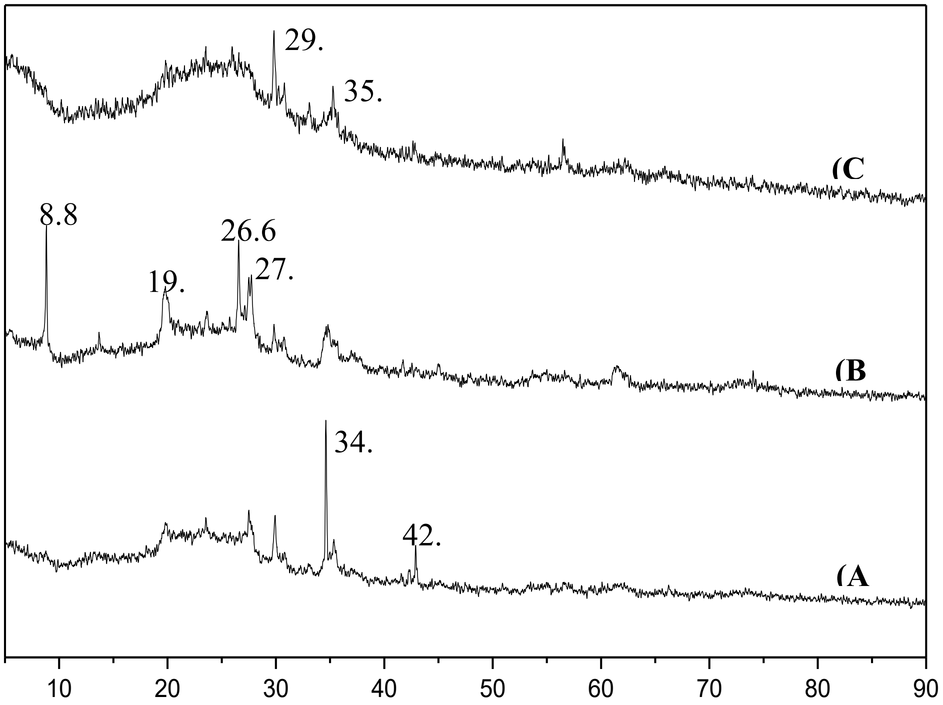

Powder XRD patterns of (A) MPI-131, (B) MPI-191, and (C) MPI-260. The major composition of MPI-260 is amorphous silica phase, as suggested by the broad feature between 20 to 30°. The diffraction patterns of MPI-131 and MPI-191 contain the main feature of the diffraction pattern of MPI-260, except different peak intensities, especially for peaks at 2θ 19.8, 27.7, and 29.8°. In addition, MPI-131 also have two peaks at 8.8 and 26.6° and MPI-191 have two peaks at 34.6 and 42.9°.

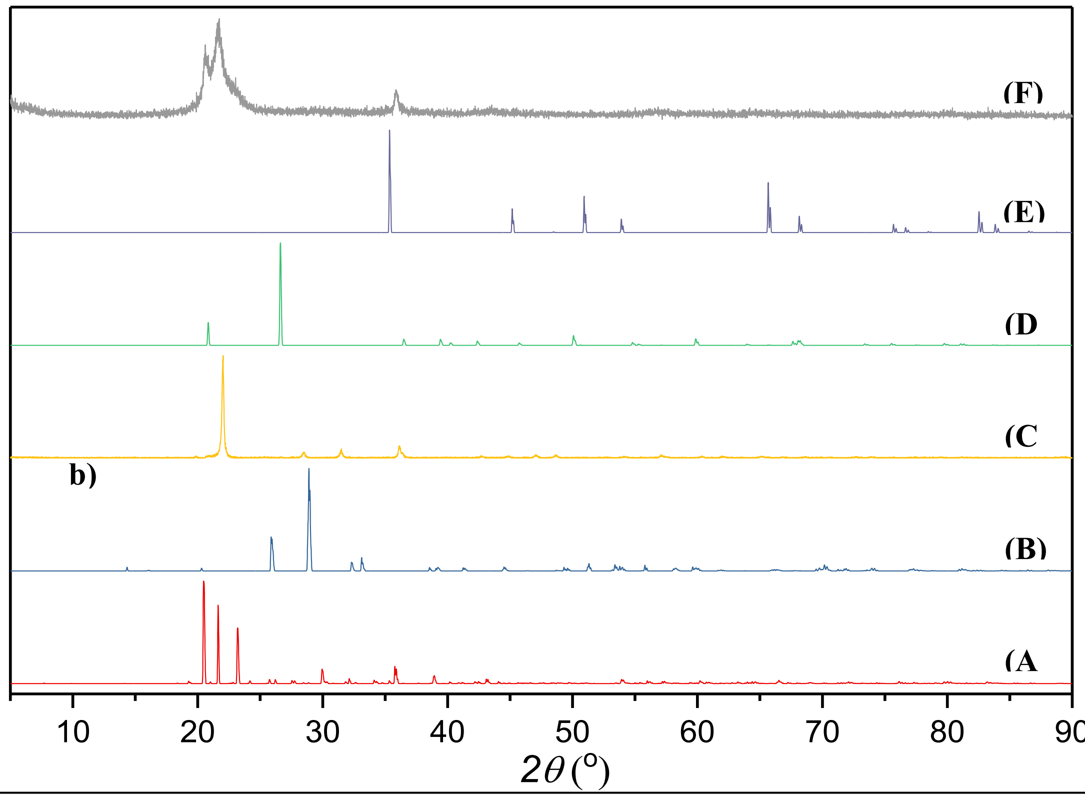

Powder XRD patterns of (A) Tridymite (R090042), (B) Coesite (R070565), (C) Cristobalite (R060648), (D) Quartz (R110108), (E) Stishovite (R070183), and (F) Opal (R060650). The XRD patterns were re-plotted with data from RRUFF database.

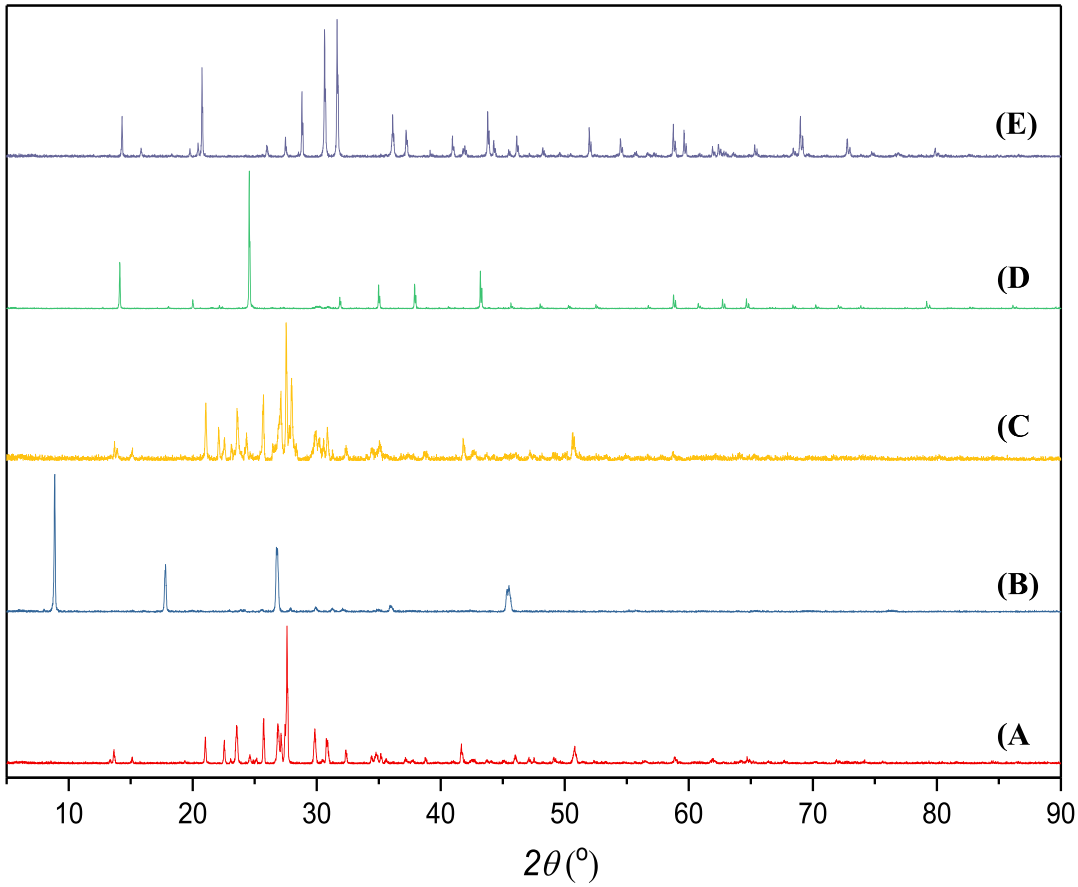

Powder XRD patterns of (A) Orthoclase (R070001), (B) Muscovite (R061120), (C) Microcline (R050193), (D) Sodalite (R060355), and (E) Jadeite (R050220). The XRD patterns were re-plotted with data from RRUFF database.

Extended Data Figure 3. Raman spectra of samples no. 131, 191, and 260 (See citation [45] for provenance details). SiO_2 exists in different types of polymorphs which include quartz, coesite, cristobalite, stishovite, tridymite, and opal (hydrated form of SiO_2). The characteristic Raman spectra were taken by the RRUFF Mineralogical Database. Silicates are an anion of silicon and oxygen and include the tetrahedral orthosilicates $(SiO_4)^{-4}$. The Raman spectra of selected silicates, including minerals such as sodalite, microcline, muscovite, and orthoclase. The Raman spectra do not appear to contain any of the signature Raman bands associated with crystalline SiO_2 polymorphs. However, a few individual particles showed well-defined Raman bands at approximately 471 cm^{-1} and 512 cm^{-1} that are very similar to that observed in the silicates microcline and orthoclase. The MPI-260 sample shows a broad band in the 450-460 cm^{-1} region that may suggest a more amorphous type SiO_2. However, the specific MPI-260 particles that show this band also have some sharp bands at 982 cm^{-1}, 1004 cm^{-1}, and 1030 cm^{-1}. Sulfate anions have Raman bands centered at approximately 980-990 cm^{-1}, 450-470 cm^{-1}, and 641-650 cm^{-1} which are associated with the $\nu_1(A1)$, $\nu_2(A1)$, and $\nu_4(A1)$ sulfate stretching modes, respectively.[5] The cation associated with the sulfate will influence the center of the vibrational band. (See spectra on next page).

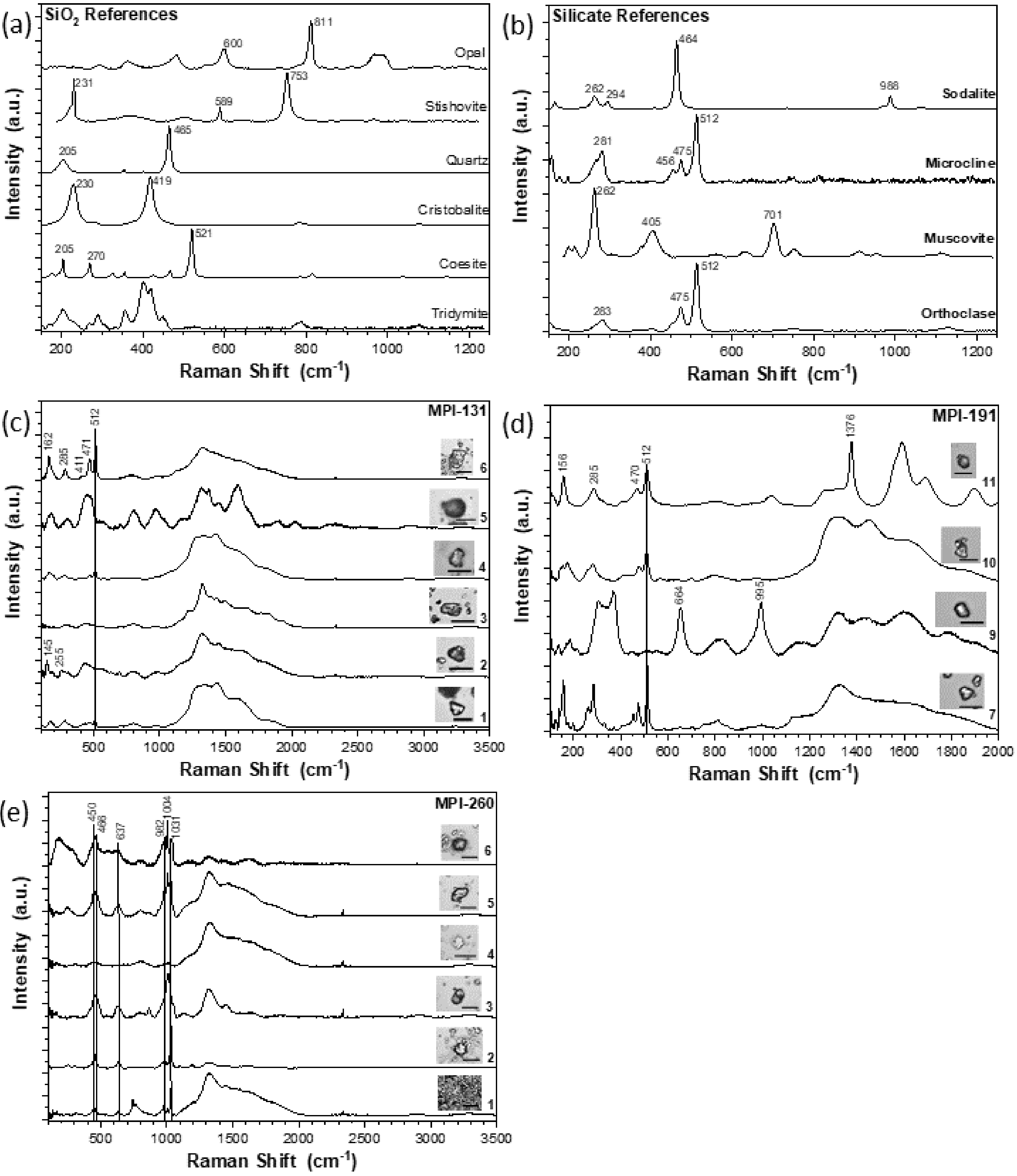
(a)
SiO2 References
Opal
Stishovite
Quartz
Cristobalite
Coesite
Tridymite
811
600
231
753
589
465
205
230
419
521
270
Intensity (a.u.)
Raman Shift (cm-1)
(b)
Silicate References
Sodalite
Microcline
Muscovite
Orthoclase
464
262
294
988
512
281
456
475
405
701
283
Intensity (a.u.)
Raman Shift (cm-1)
(c)
MPI-131
162
285
411
471
512
145
255
Intensity (a.u.)
Raman Shift (cm-1)
(d)
MPI-191
156
285
470
512
1376
664
995
Intensity (a.u.)
Raman Shift (cm-1)
(e)
MPI-260
450
466
637
982
1004
1031
Intensity (a.u.)
Raman Shift (cm-1)

Extended Data Figure 4. Residue heat maps (Nearest Neighbor Index) and kernel density estimation from selected specimens. Top panel: Surface specimen north 47 (See Figure 1, center right for provenance). Bottom panel: Subsurface specimen no. 5 (See Figure 1, bottom panel for provenance). Statistical analysis of georeferenced residue at the mm scale displays disperse scatters in which red holds the highest density and dark green the lowest.

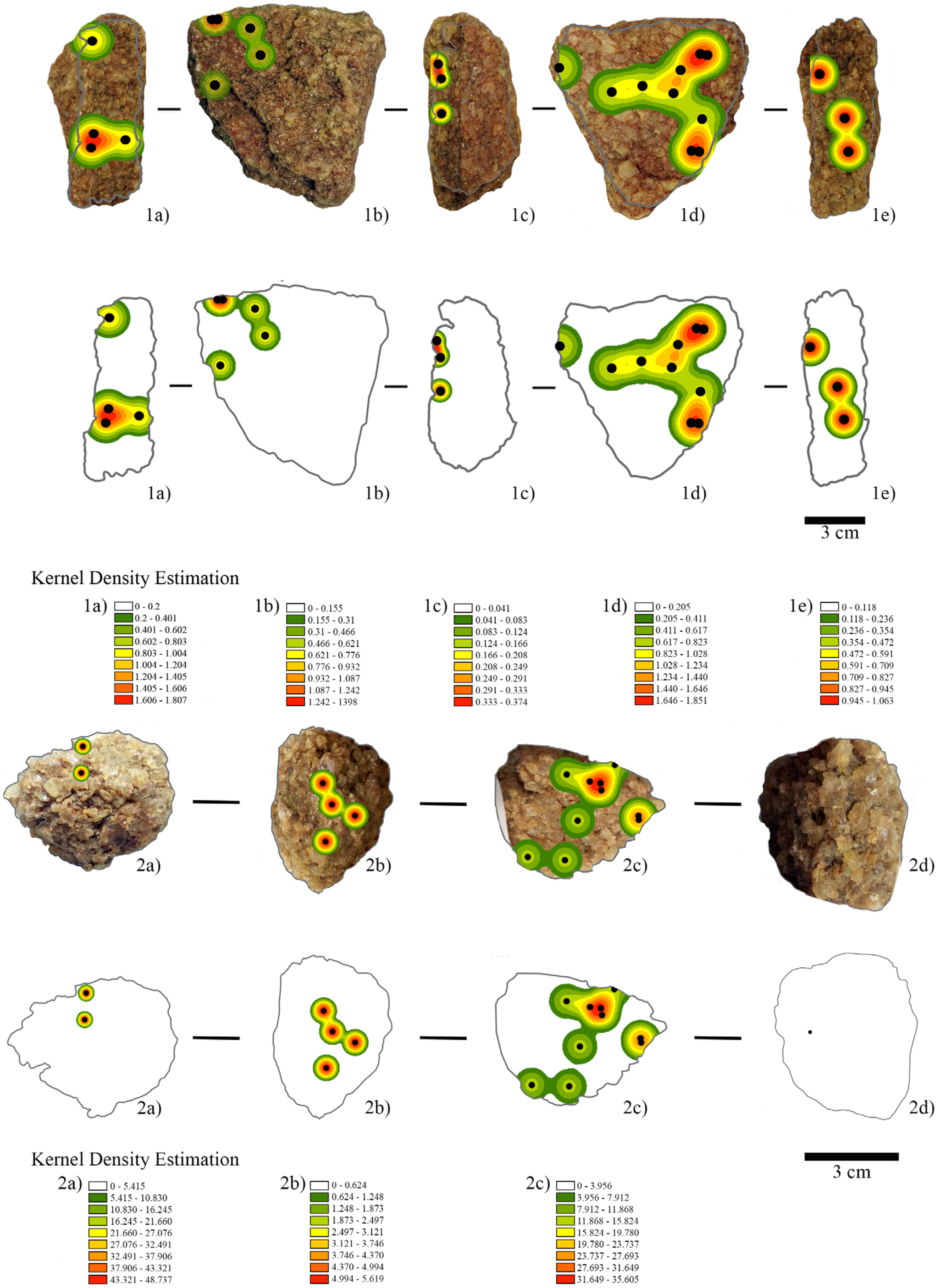

Extended Data Figure 5. Baseline of natural and experimental rock coatings from Oldupai Gorge. All specimens photographed after cleaning cycle no. 4.

SURFACE STONES

No. 17

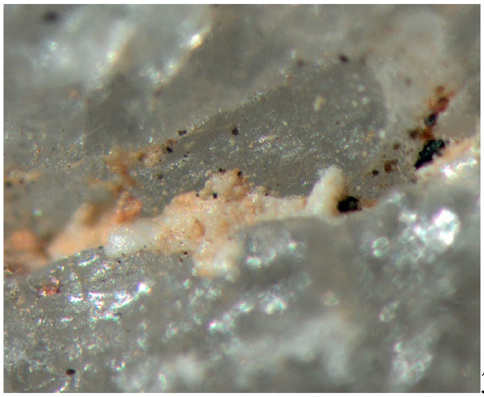

32x

Yellowish brown waxy accretion

No. 17

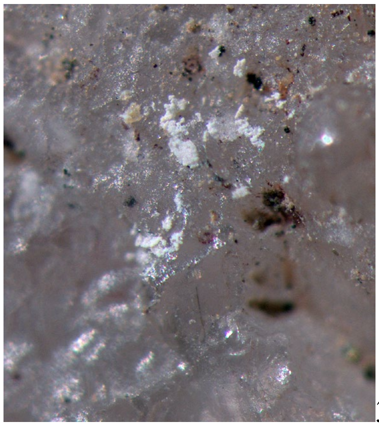

32x

White crust

No. 17

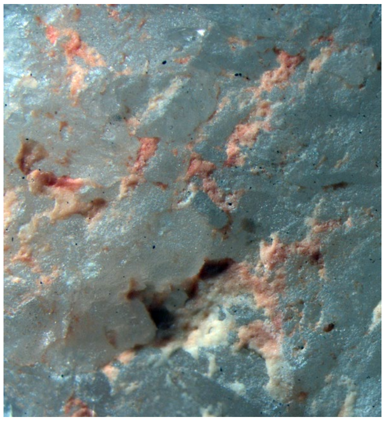

10x

White crust, staining

No. 17

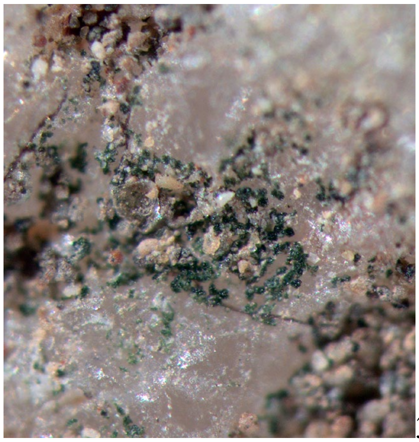

32x

Lithobiont

No. 17

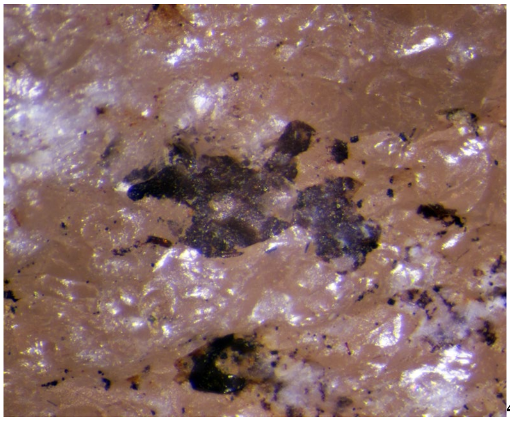

4x

Dark brown oxides

No. 17

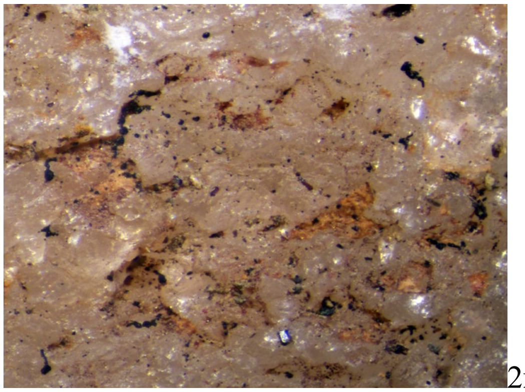

2x

Dark brown mass/crust coalescence

No 31.

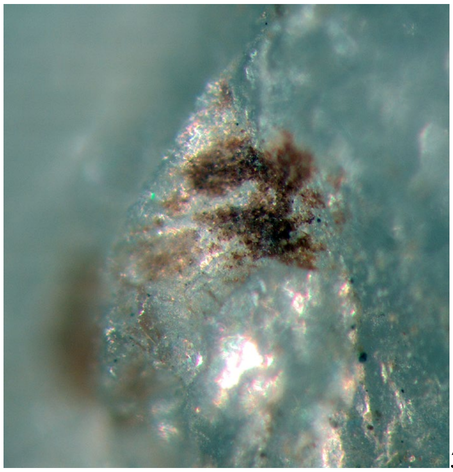
50x

Dark brown masses

No 31.

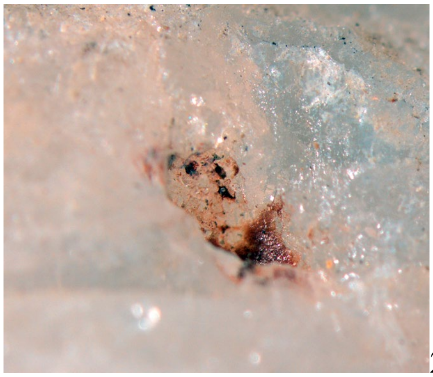
20x

Brown coating

No 31.

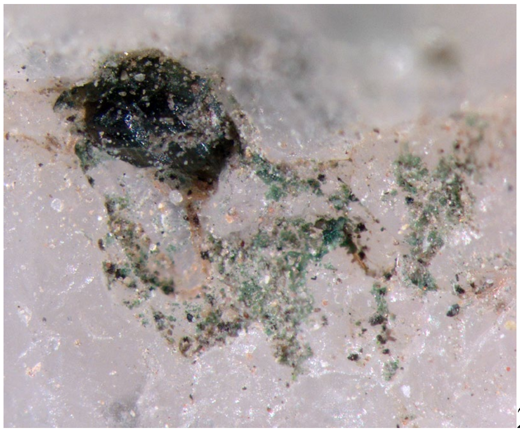
25x

Lithobiont

No 31.

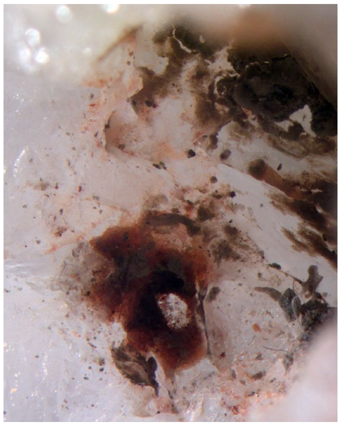

15x

Waxy dark brown film

No 31.

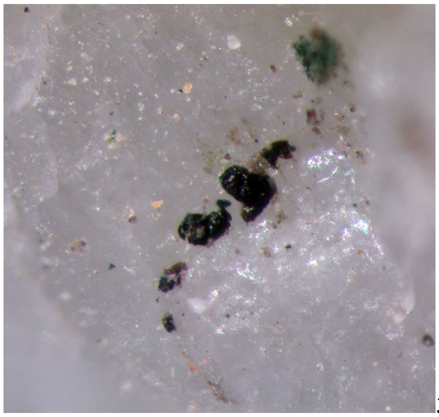

50x

Sinuous black concretions

No. 31

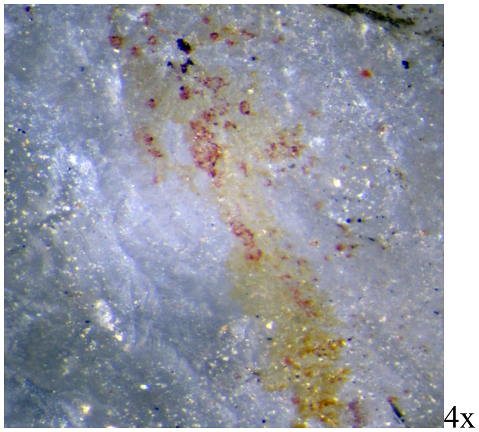

4x

Yellowish film and oxides coalescing

No. 31

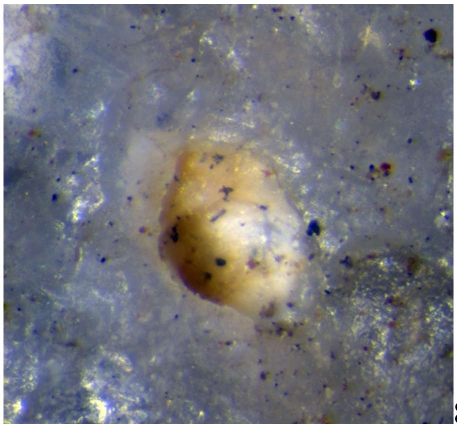

8x

Yellowish waxy accretion

No. 39

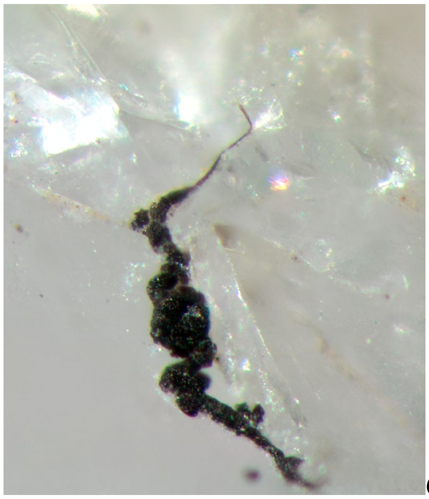

63x

Sinuous black concretions

No. 39

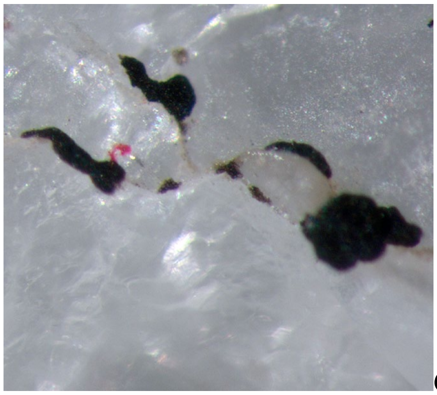

63x

Sinuous black concretions

No. 39

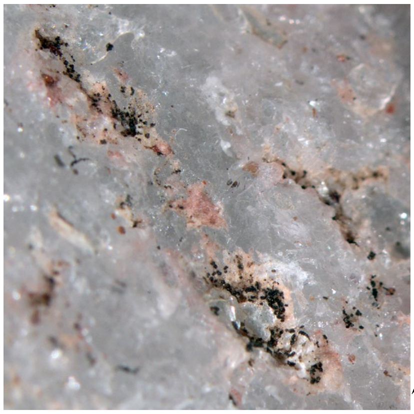
7x

Crust and black powdery coating

No. 39

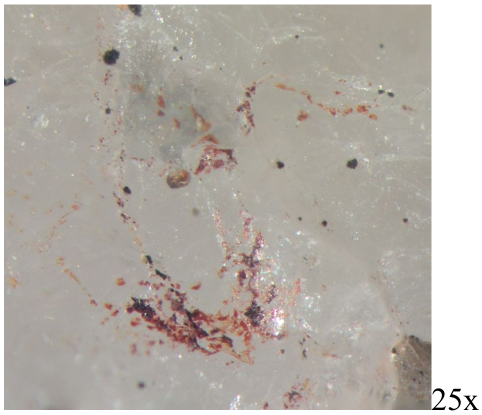
25x

Dark red glaze

No. 39

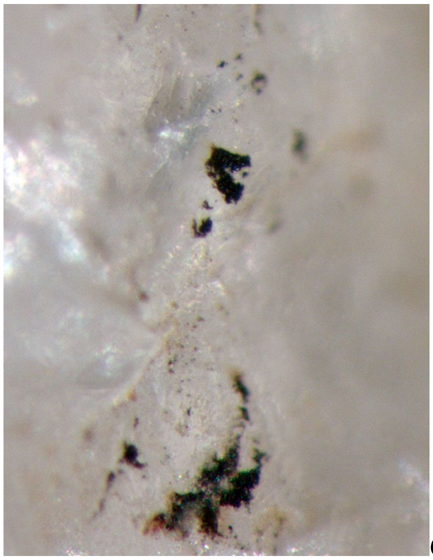
63x

Black concretion

No. 39

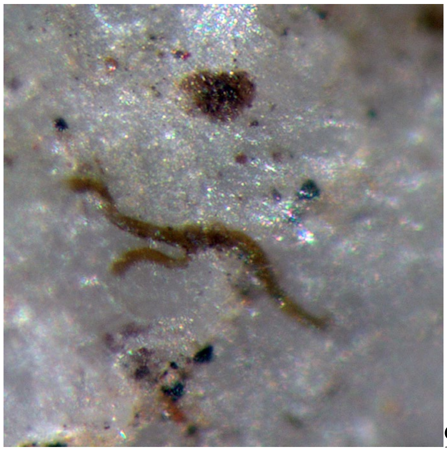

90x

Lithobiont

No. 39

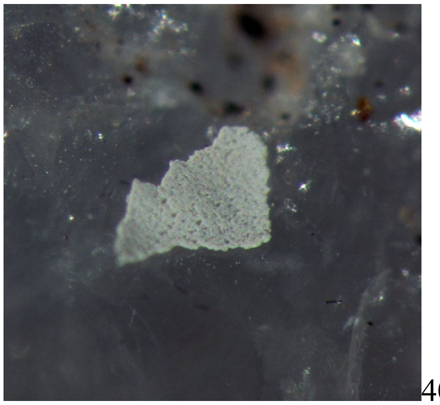

40x

Crust

No. 39

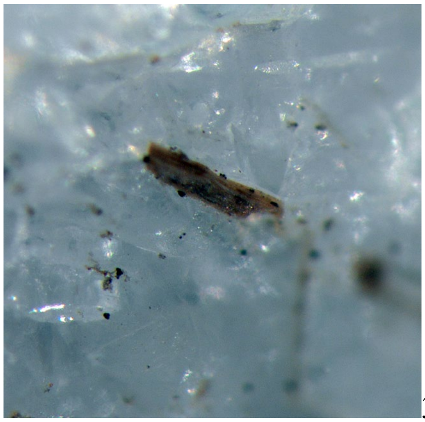

32x

Biogenic clast

No. 42

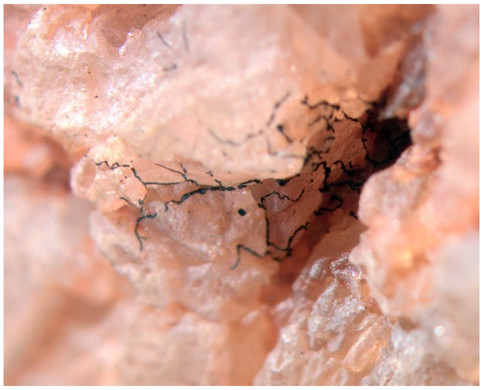

10x

Lithobiont

No. 47

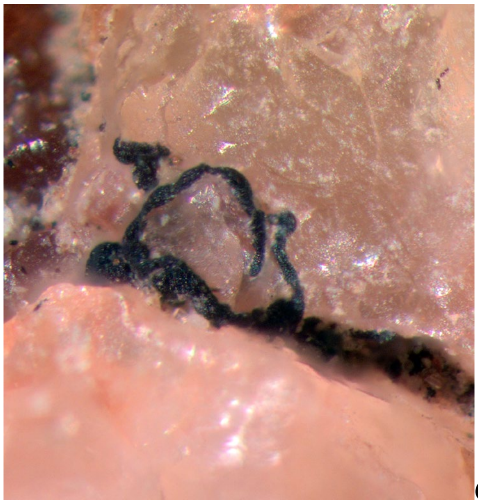

63x

Sinuous black concretions

No. 47

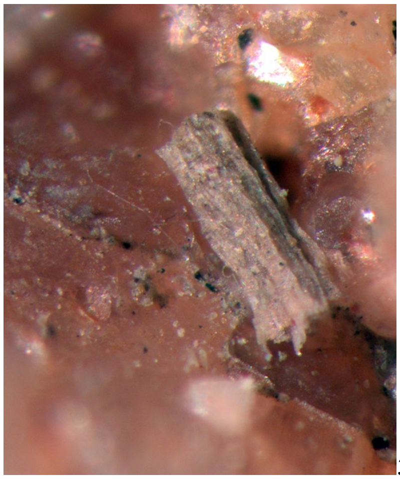

50x

Biogenic clast

SUBSURFACE STONES

No. 5

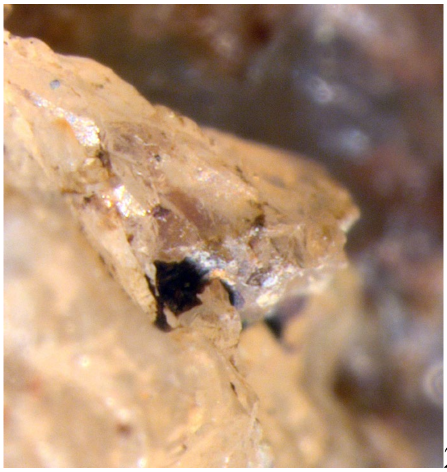

20x

Black coating

No. 5

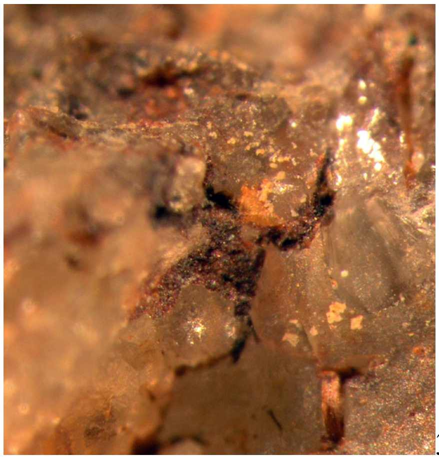

32x

Dark brown oxides

No. 5

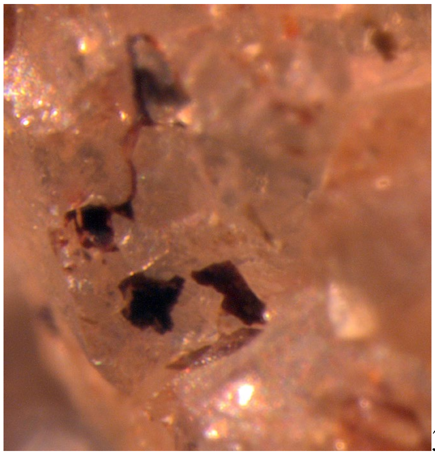

32x

Waxy dark brown film

No. 5

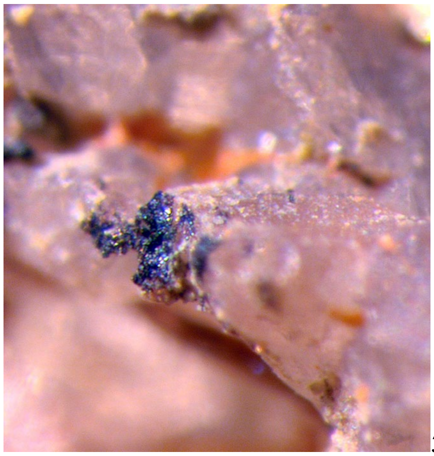

50x

Black coating

No. 7

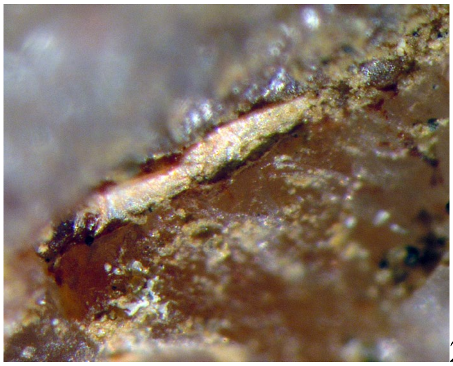

25x

Biogenic clast

No. 8

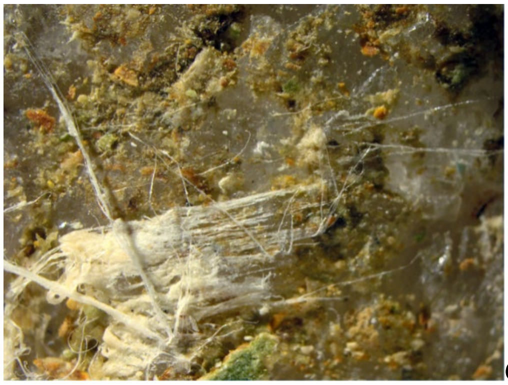

63x

Aligned fibrous strands

No. 8

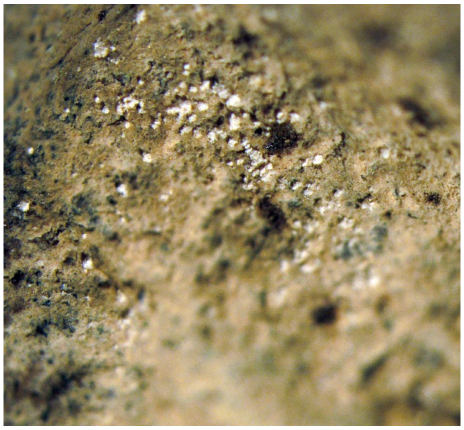
10x

Salt precipitates

No. 8

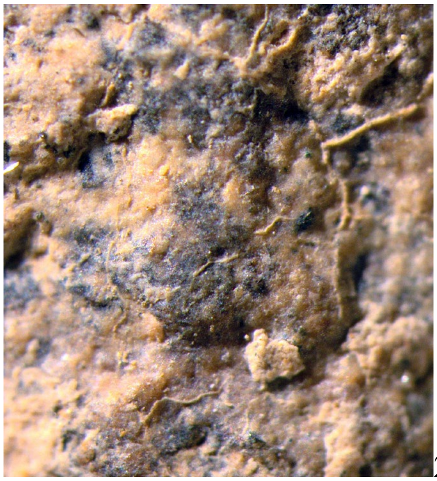
20x

Glaze and tubular secretions

No. 29

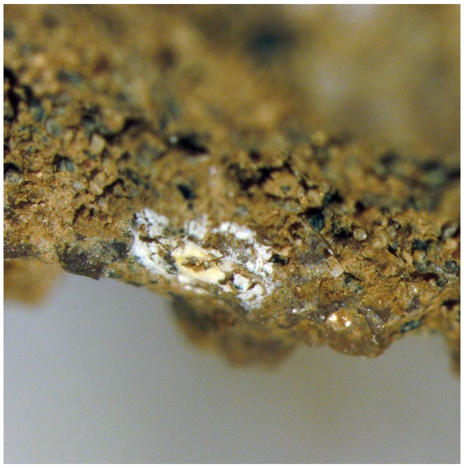
16x

White crust

No. 29

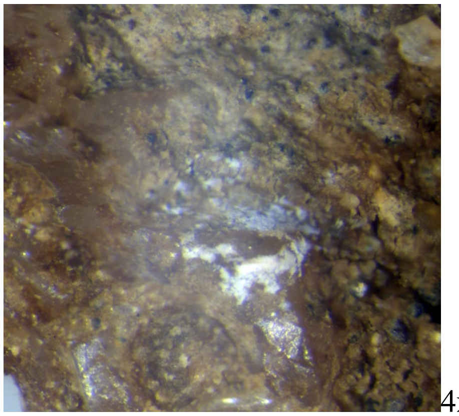

4x

White crust

EXPERIMENTAL TOOLS

1-1

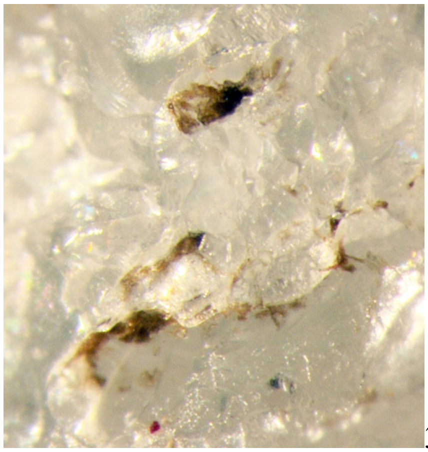

38x

Brown masses

1-1

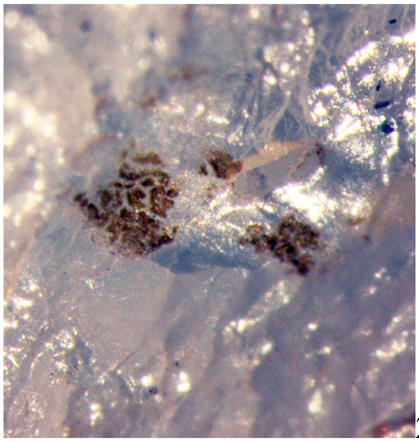

20x

Waxy dark brown film

1-1

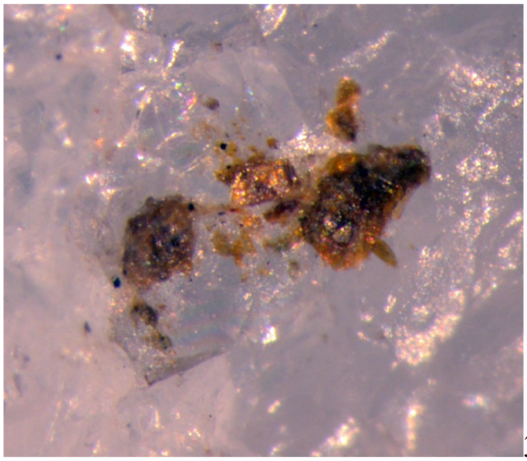

35x

Brown masses

1-2

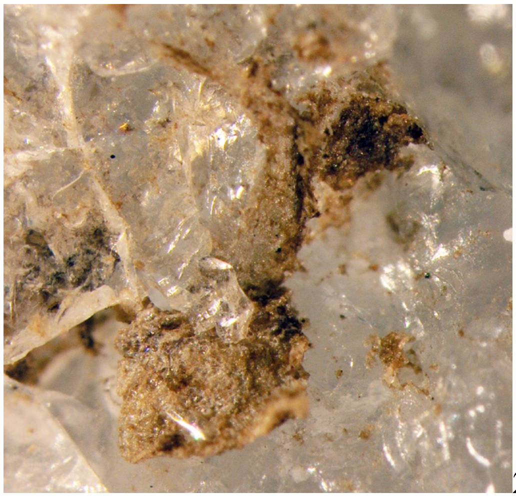

20x

Brown masses, epidermal tissue

1-2

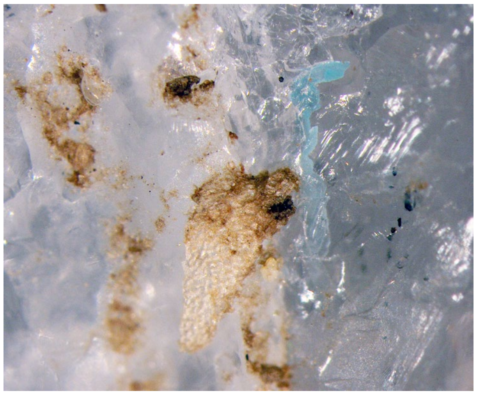

15x

Brown masses, epidermal tissue

1-2

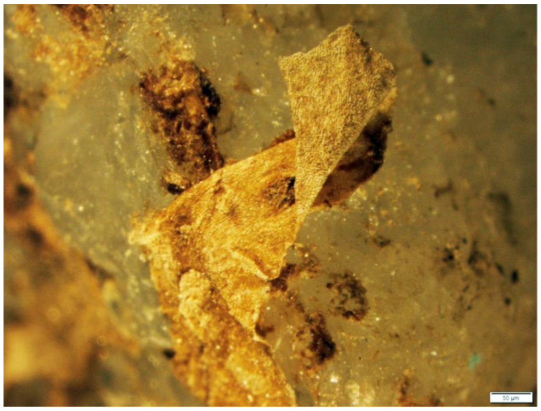

Brown masses, epidermal tissue

2-2

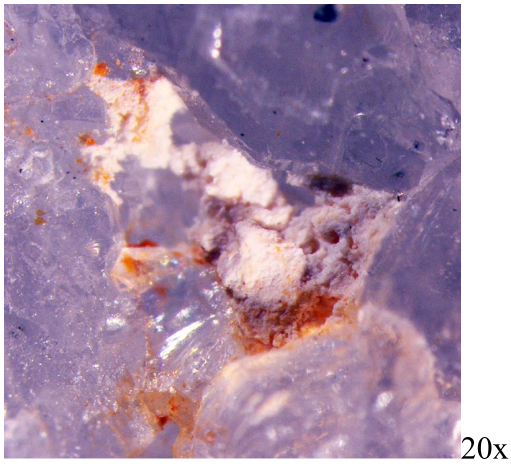

20x

Oxide and crust coalescence

2-2

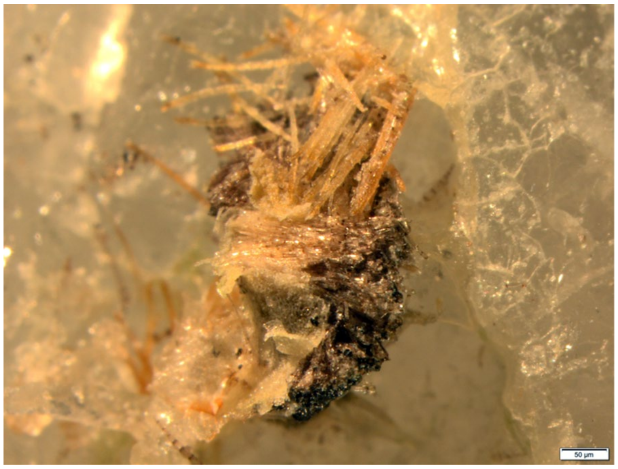

Spinous tissue

9-1

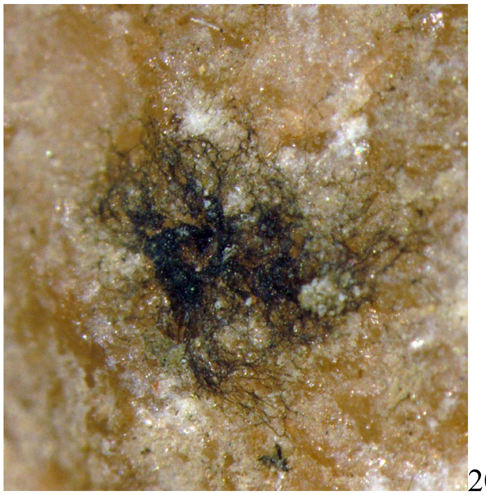

20x

Lithobiont mat

12-1

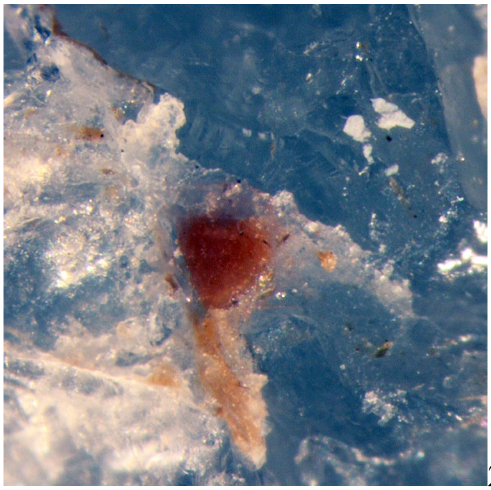

25x

Waxy mass

12-1

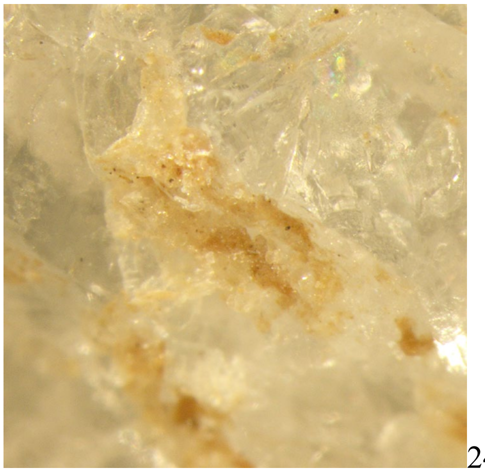

24x

Bone tissue

12-1

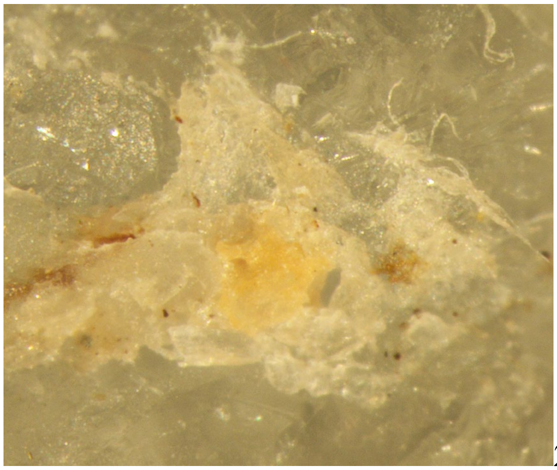
25x

Bone tissue

12-1

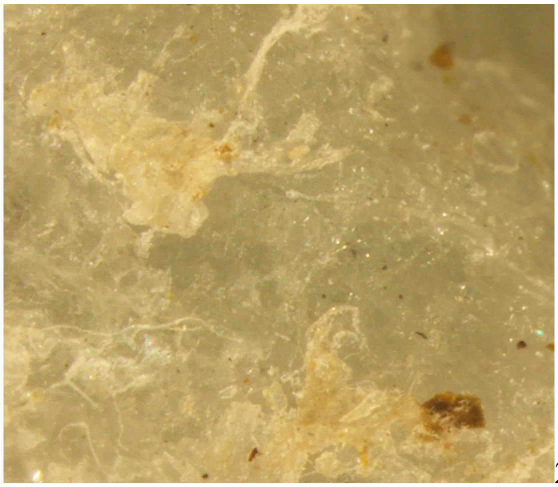
24x

Bone tissue

Extended Data Figure 6. Surface stone no. 42, Facet A, Coating 4

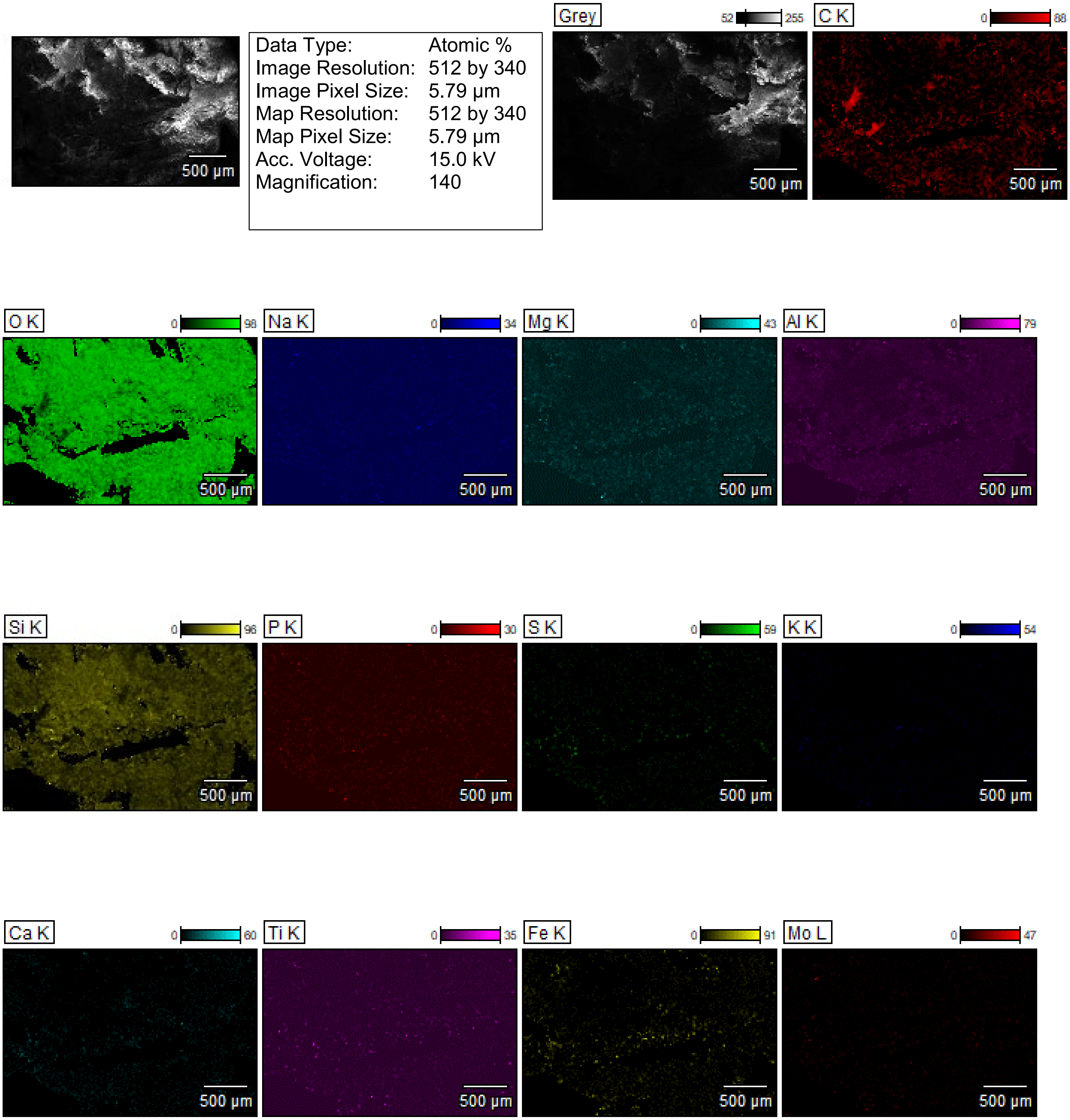

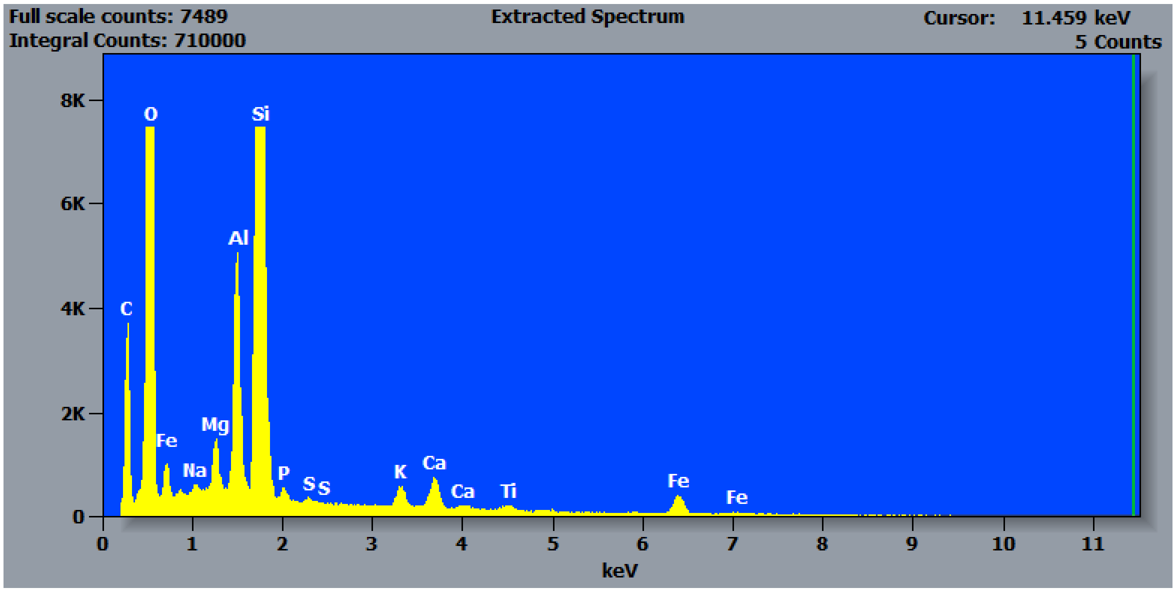

Surface 42 (4)						
**Element**	**Net Counts**	**Weight %**	**Weight % err**	**Atom %**	**Norm. Wt. %**	**Chemical Formula**
C K	20768	9.38	0.08	14.60	9.38	C
O K	182173	52.45	0.27	61.25	52.45	O
Na K	1064	0.16	0.03	0.13	0.16	Na
Mg K	8020	0.91	0.02	0.70	0.91	Mg
Al K	37107	3.66	0.04	2.53	3.66	Al
Si K	275249	27.73	0.11	18.45	27.73	Si
P K	3475	0.41	0.02	0.25	0.41	P
S K	672	0.08	0.01	0.04	0.08	S
K K	4661	0.75	0.02	0.36	0.75	K
Ca K	7216	1.29	0.02	0.60	1.29	Ca
Ti K	1387	0.38	0.05	0.15	0.38	Ti
Fe K	5113	2.81	0.12	0.94	2.81	Fe
**% Total**	-	**100**	-	**100**	**100**	-

Surface 42, Facet A, Coating 6.

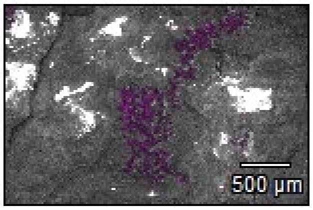

Data Type:	Atomic %
Image Resolution:	512 by 340
Image Pixel Size:	6.23 µm
Map Resolution:	512 by 340
Map Pixel Size:	6.23 µm
Acc. Voltage:	15.0 kV
Magnification:	130

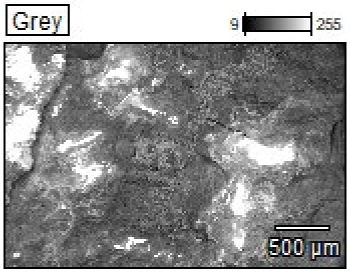

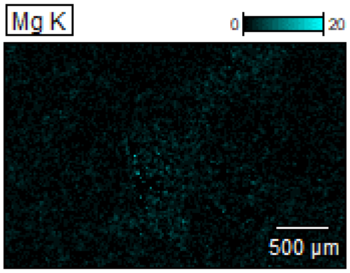

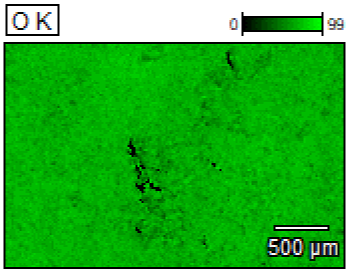

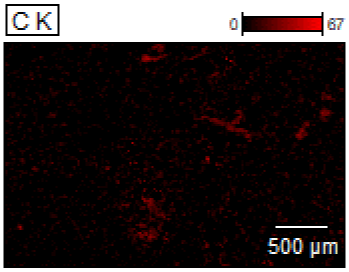

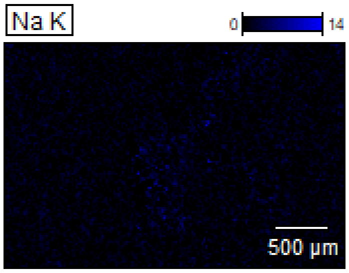

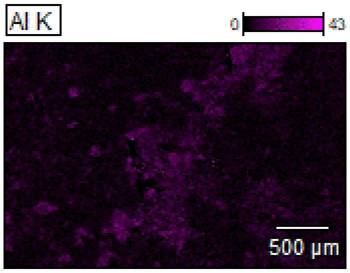

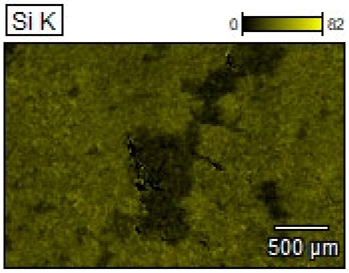

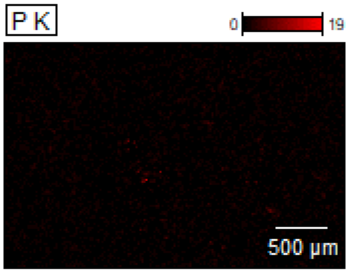

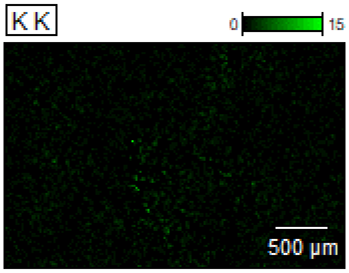

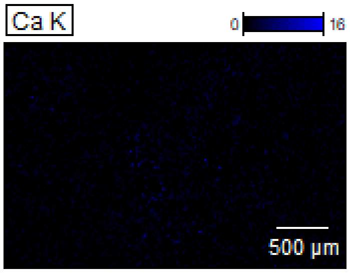

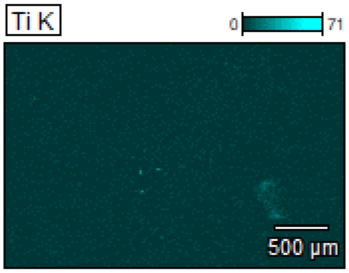

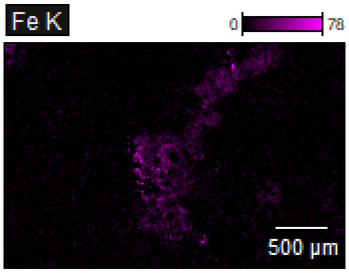

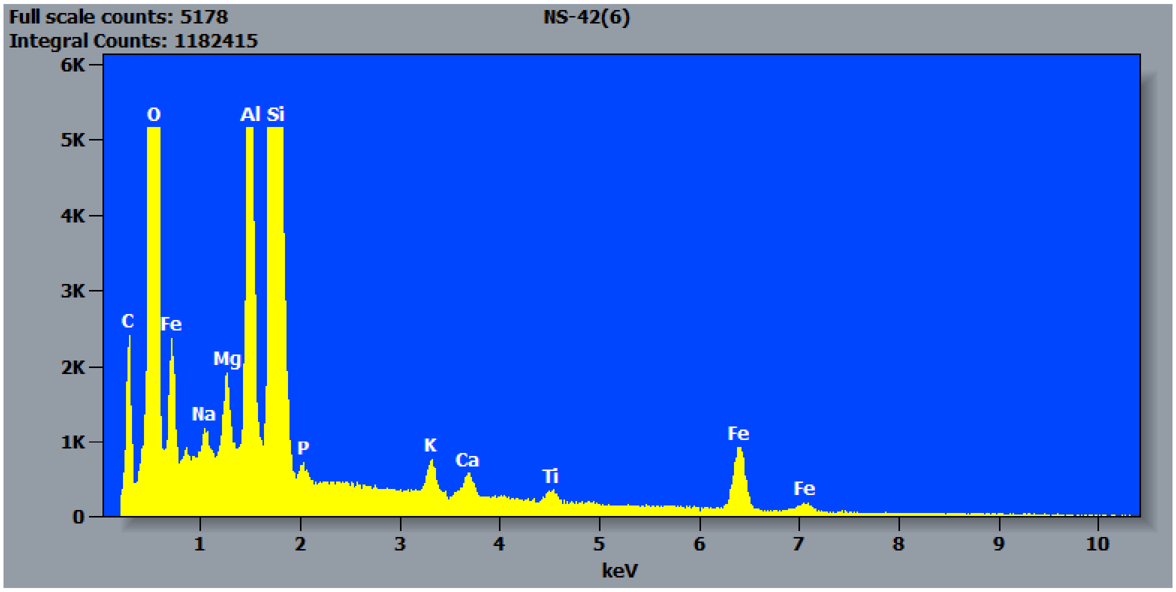

Surface 42 (6)						
**Element**	**Net Counts**	**Weight %**	**Weight % err**	**Atom %**	**Norm. Wt. %**	**Chemical Formula**
C K	12138	3.54	0.05	5.73	3.54	C
O K	360392	55.88	0.27	67.89	55.88	O
Na K	2181	0.21	0.02	0.18	0.21	Na
Mg K	8449	0.62	0.02	0.49	0.62	Mg
Al K	58927	3.74	0.03	2.69	3.74	Al
Si K	458837	29.76	0.11	20.60	29.76	Si
P K	3911	0.30	0.02	0.19	0.30	P
K K	5007	0.52	0.01	0.26	0.52	K
Ca K	3712	0.43	0.01	0.21	0.43	Ca
Ti K	2190	0.38	0.02	0.15	0.38	Ti
Fe K	13055	4.63	0.10	1.61	4.63	Fe
**% Total**	-	**100**	-	**100**	**100**	-

Extended Data Figure 7.
Surface stone no. 31 Facet B, coating 1

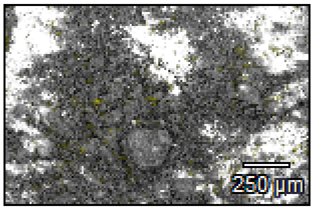

Data Type:	Atomic %
Image Resolution:	512 by 340
Image Pixel Size:	3.38 µm
Map Resolution:	512 by 340
Map Pixel Size:	3.38 µm
Acc. Voltage:	15.0 kV
Magnification:	240

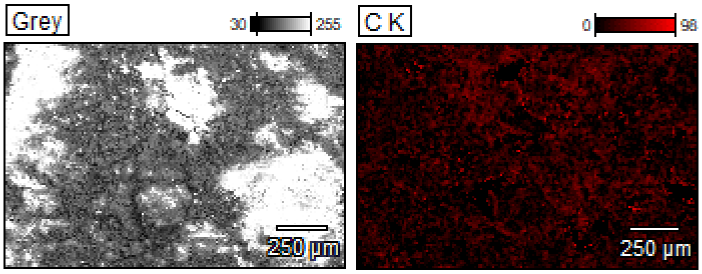

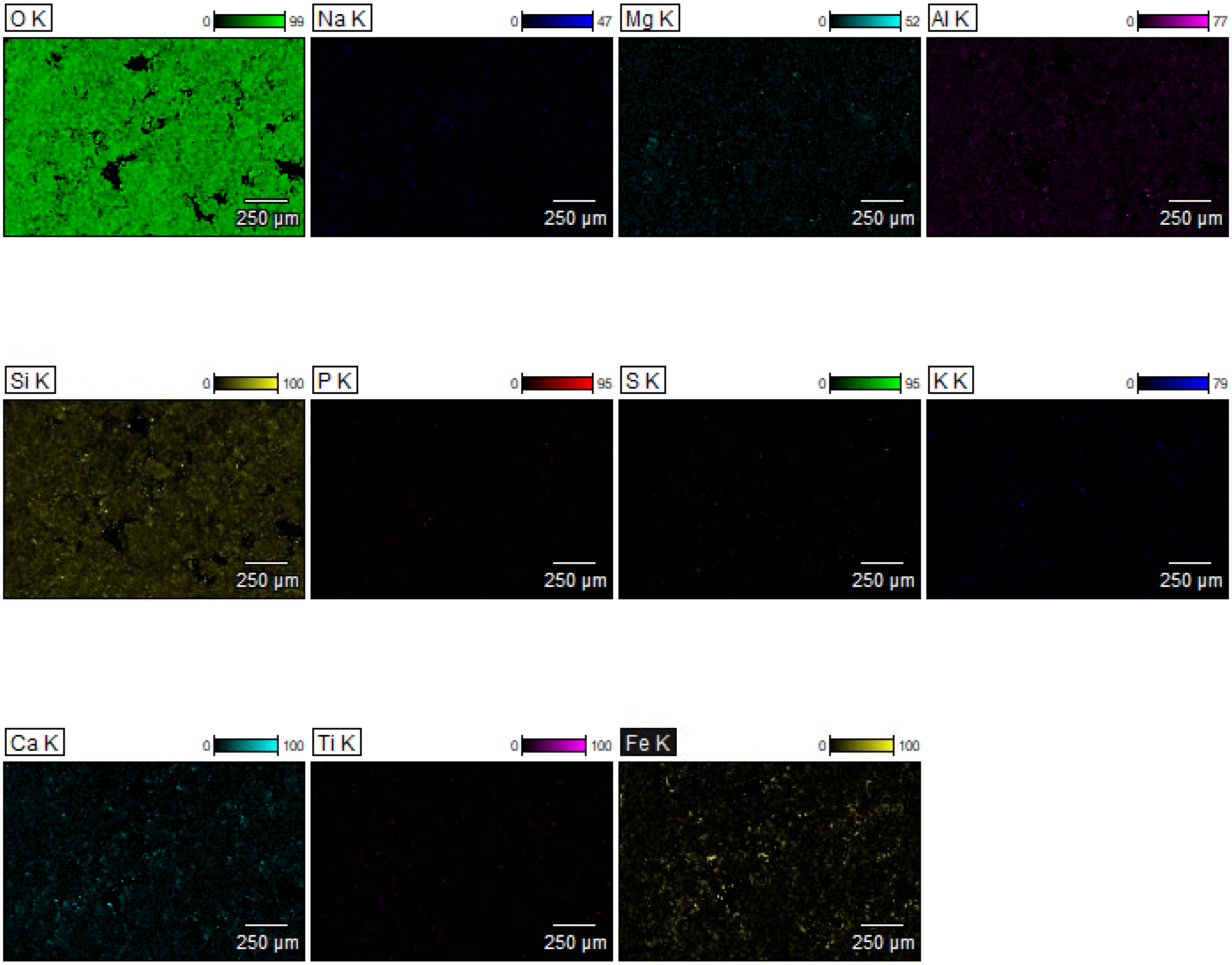

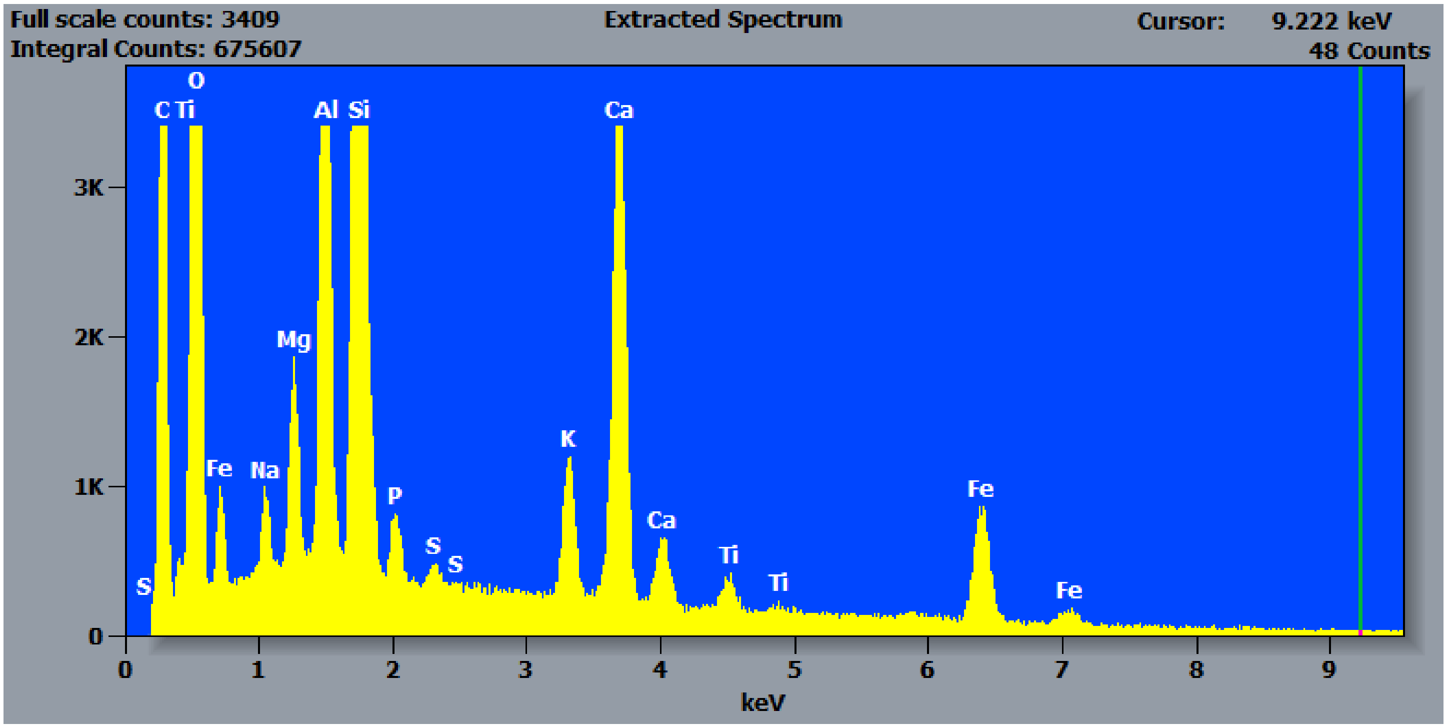

Surface E 31 B (1)						
**Element**	**Net Counts**	**Weight %**	**Weight % err**	**Atom %**	**Norm. Wt. %**	**Chemical Formula**
C K	40876	13.63	0.10	21.56	13.63	C
O K	135913	46.77	0.31	55.54	46.77	O
Na K	4155	0.63	0.03	0.52	0.63	Na
Mg K	10929	1.25	0.03	0.98	1.25	Mg
Al K	49903	4.91	0.04	3.46	4.91	Al
Si K	157678	15.71	0.08	10.62	15.71	Si
P K	5571	0.59	0.03	0.36	0.59	P
S K	1878	0.19	0.03	0.11	0.19	S
K K	10969	1.61	0.04	0.78	1.61	K
Ca K	45541	7.60	0.07	3.60	7.60	Ca
Ti K	3194	0.82	0.05	0.32	0.82	Ti
Fe K	12066	6.27	0.15	2.13	6.27	Fe
**% Total**	**-**	**100**	**-**	**100**	**100**	**-**

Surface East 31 Facet B, Coating 2

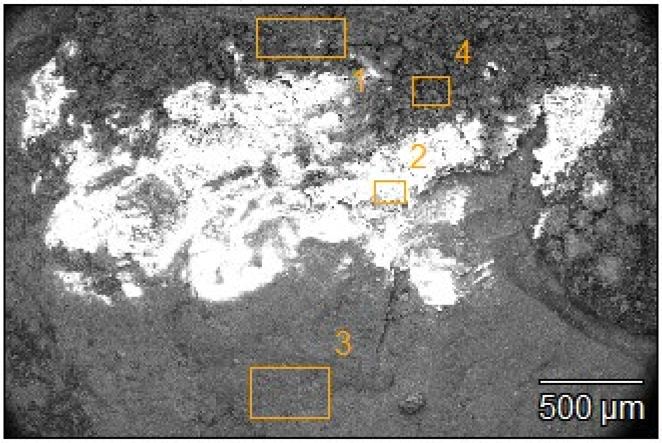

Image Name:	NS-E31-B(2)
Image Resolution:	512 by 340
Image Pixel Size:	6.23 µm
Acc. Voltage:	15.0 kV
Magnification:	130

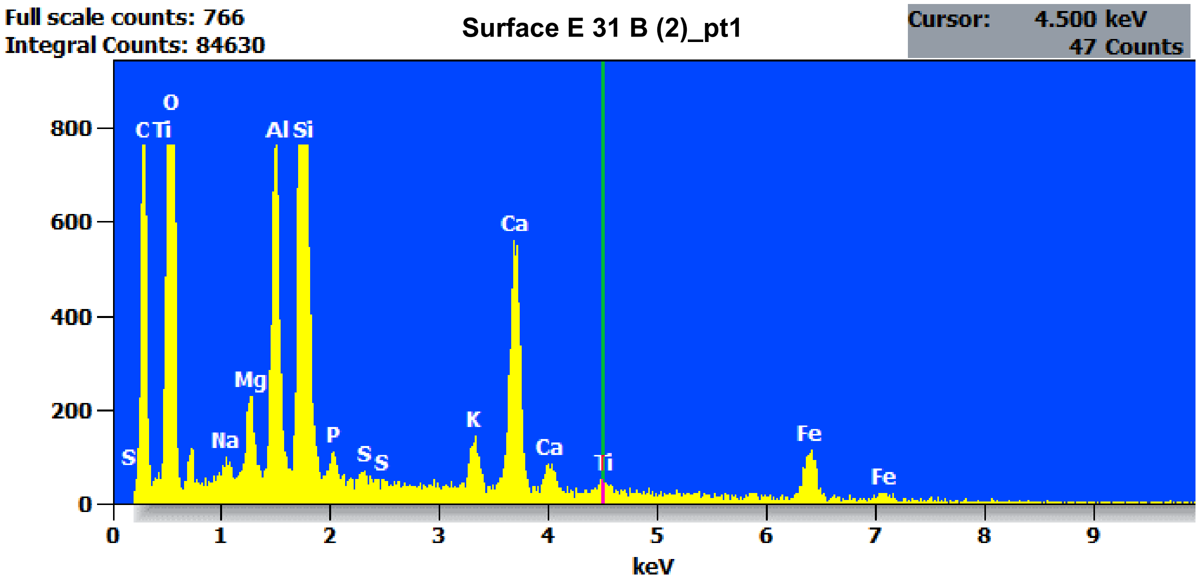

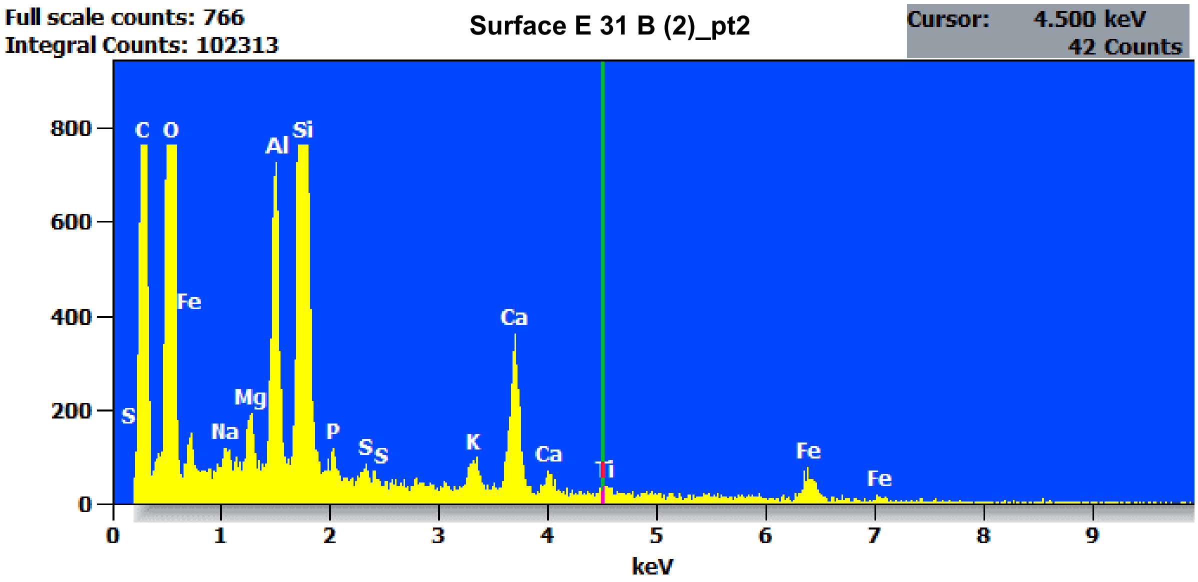

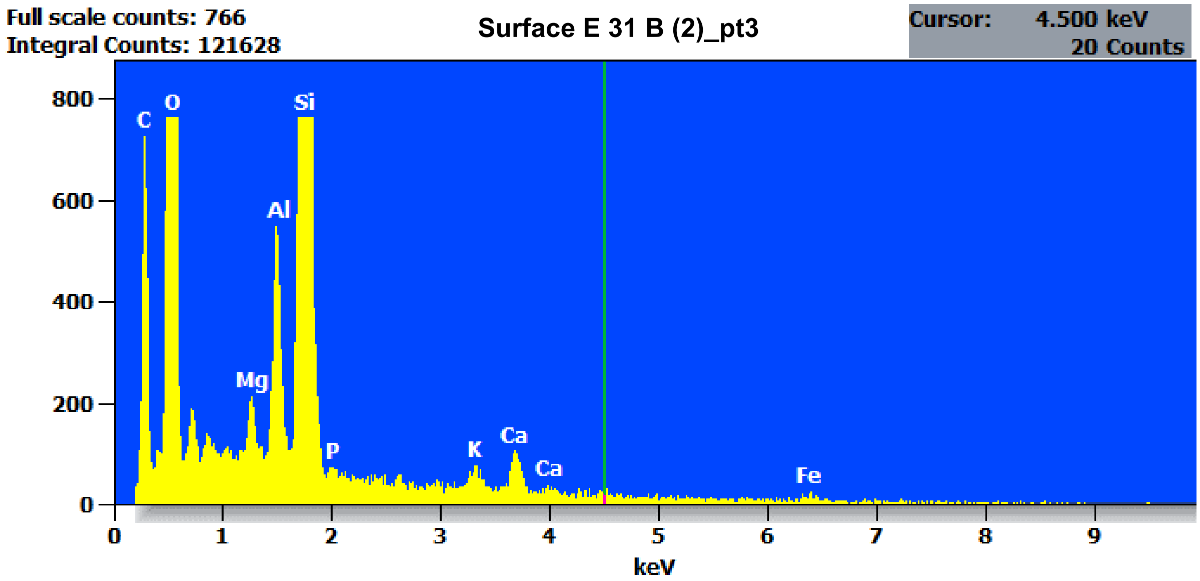
Full scale counts: 766
Integral Counts: 121628
Surface E 31 B (2)_pt3
Cursor: 4.500 keV
20 Counts
800
600
400
200
0
C
O
Si
Al
Mg
P
K
Ca
Ca
Fe
0
1
2
3
4
5
6
7
8
9
keV

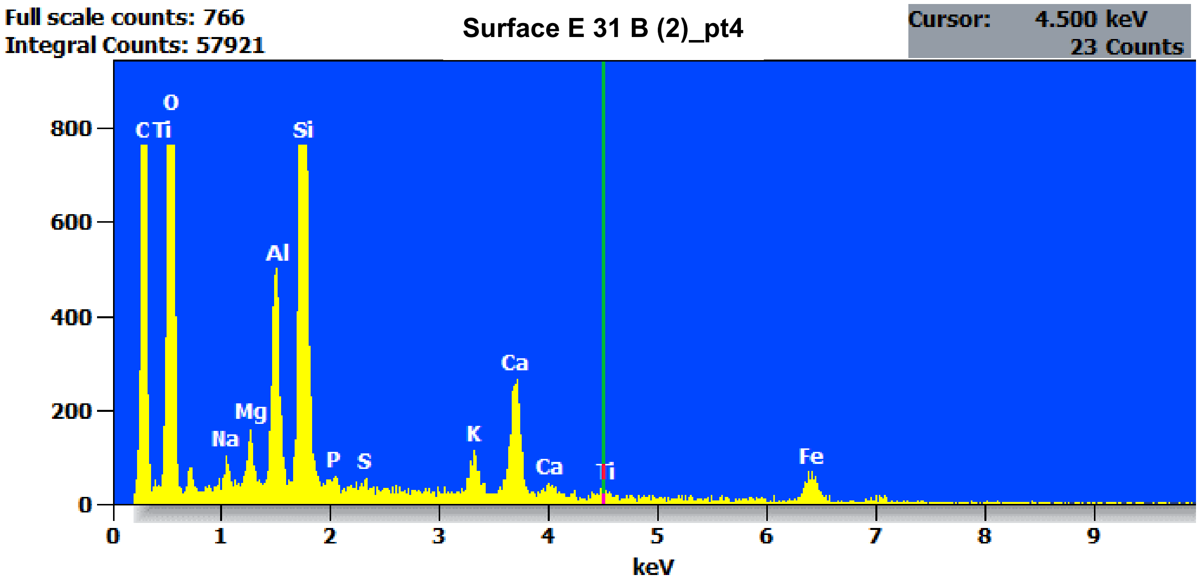
Full scale counts: 766
Integral Counts: 57921
Surface E 31 B (2)_pt4
Cursor: 4.500 keV
23 Counts
800
600
400
200
0
O
C Ti
Si
Al
Mg
Na
P
S
K
Ca
Ca
Ti
Fe
0
1
2
3
4
5
6
7
8
9
keV

Net Counts

	C	O	Na	Mg	Al	Si	P	S	K	Ca	Ti	Fe
Surface E 31 B (2)_pt1	5583	16811	331	1372	6061	19307	634	183	1247	6263	424	1458
Surface E 31 B (2)_pt2	15513	25732	372	837	5173	21351	581	346	898	3677	245	759
Surface E 31 B (2)_pt3	3828	37194		810	3648	46819	258	-	564	948	-	181
Surface E 31 B (2)_pt4	7221	11904	313	624	3858	11460	286	202	983	2842	183	891

Weight %

	C	O	Na	Mg	Al	Si	P	S	K	Ca	Ti	Fe
Surface E 31 B (2)_pt1	14.34	46.81	0.40	1.24	4.72	15.21	0.53	0.15	1.45	8.28	0.86	6.01
Surface E 31 B (2)_pt2	24.70	51.43	0.33	0.56	2.96	12.28	0.35	0.21	0.79	3.66	0.37	2.37
Surface E 31 B (2)_pt3	9.53	58.60	-	0.54	2.09	26.99	0.18	-	0.52	0.99	-	0.58
Surface E 31 B (2)_pt4	22.58	46.56	0.52	0.78	4.14	12.42	0.32	0.22	1.59	5.22	0.52	5.12

Weight % Error (+/- 1 Sigma)

	C	O	Na	Mg	Al	Si	P	S	K	Ca	Ti	Fe
Surface E 31 B (2)_pt1	±0.20	±0.45	±0.05	±0.05	±0.06	±0.15	±0.04	±0.04	±0.06	±0.19	±0.15	±0.42
Surface E 31 B (2)_pt2	±0.22	±0.45	±0.04	±0.04	±0.08	±0.12	±0.03	±0.03	±0.08	±0.12	±0.05	±0.28
Surface E 31 B (2)_pt3	±0.15	±0.41	-	±0.06	±0.05	±0.16	±0.04	-	±0.08	±0.05	-	±0.11
Surface E 31 B (2)_pt4	±0.27	±0.55	±0.06	±0.09	±0.07	±0.11	±0.05	±0.04	±0.07	±0.19	±0.09	±0.48

Normalized Wt. %

	C	O	Na	Mg	Al	Si	P	S	K	Ca	Ti	Fe
Surface E 31 B (2)_pt1	14.34	46.81	0.40	1.24	4.72	15.21	0.53	0.15	1.45	8.28	0.86	6.01
Surface E 31 B (2)_pt2	24.70	51.43	0.33	0.56	2.96	12.28	0.35	0.21	0.79	3.66	0.37	2.37
Surface E 31 B (2)_pt3	9.53	58.60	-	0.54	2.09	26.99	0.18		0.52	0.99	-	0.58
Surface E 31 B (2)_pt4	22.58	46.56	0.52	0.78	4.14	12.42	0.32	0.22	1.59	5.22	0.52	5.12

Atom %

	C	O	Na	Mg	Al	Si	P	S	K	Ca	Ti	Fe
Surface E 31 B (2)_pt1	22.55	55.25	0.33	0.97	3.31	10.22	0.32	0.09	0.70	3.90	0.34	2.03
Surface E 31 B (2)_pt2	34.08	53.27	0.24	0.38	1.82	7.24	0.19	0.11	0.33	1.51	0.13	0.70
Surface E 31 B (2)_pt3	14.24	65.75	-	0.40	1.39	17.25	0.10	-	0.24	0.44	-	0.19
Surface E 31 B (2)_pt4	32.81	50.77	0.39	0.56	2.68	7.71	0.18	0.12	0.71	2.27	0.19	1.60

Formula

	C	O	Na	Mg	Al	Si	P	S	K	Ca	Ti	Fe
Surface E 31 B (2)_pt1	C	O	Na	Mg	Al	Si	P	S	K	Ca	Ti	Fe
Surface E 31 B (2)_pt2	C	O	Na	Mg	Al	Si	P	S	K	Ca	Ti	Fe
Surface E 31 B (2)_pt3	C	O	-	Mg	Al	Si	P	-	K	Ca	-	Fe
Surface E 31 B (2)_pt4	C	O	Na	Mg	Al	Si	P	S	K	Ca	Ti	Fe

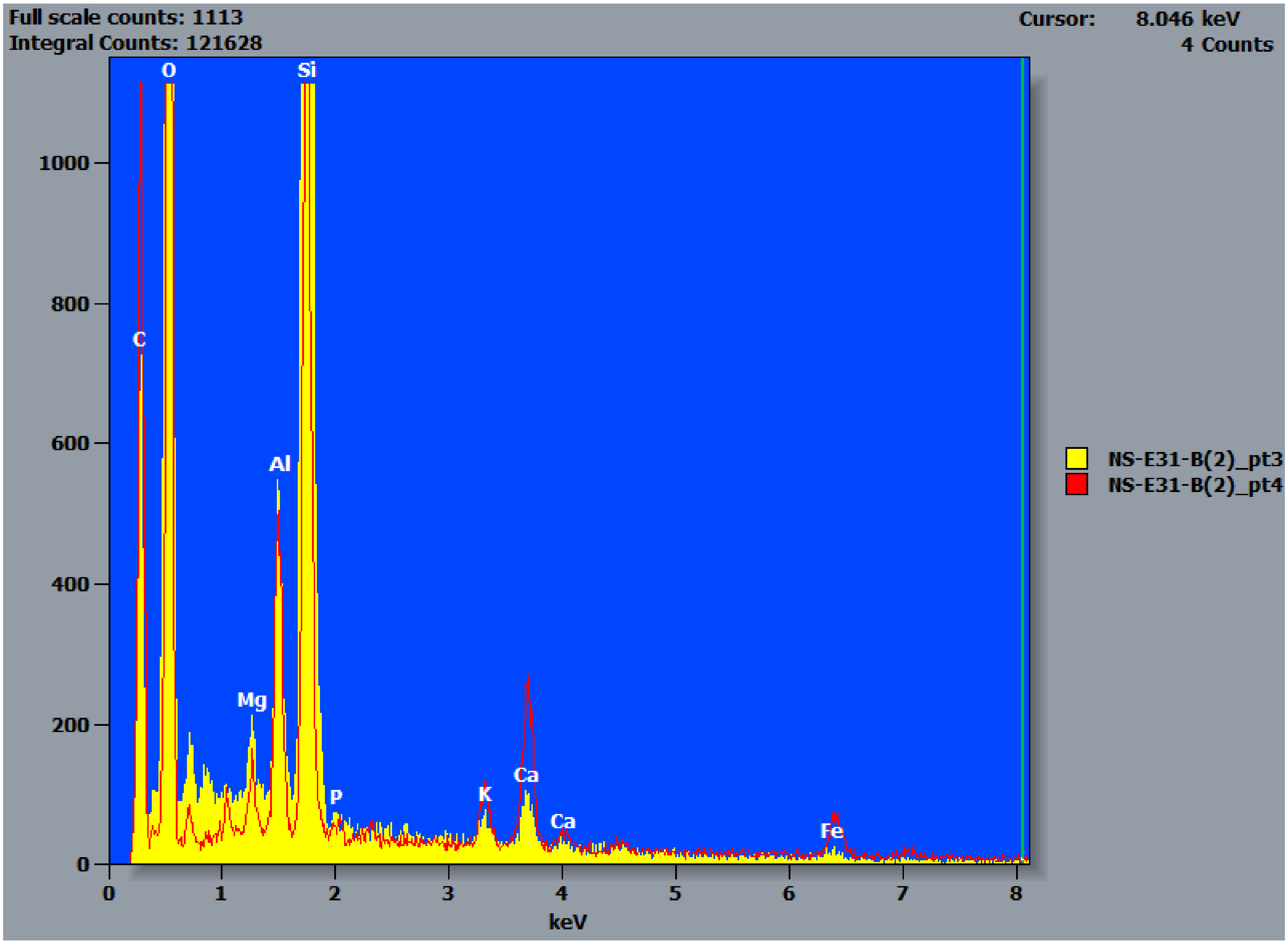

Full scale counts: 1113
Integral Counts: 121628
Cursor: 8.046 keV
4 Counts
C
O
Si
Al
Mg
P
K
Ca
Ca
Fe
1000
800
600
400
200
0
0
1
2
3
4
5
6
7
8
keV
NS-E31-B(2)_pt3
NS-E31-B(2)_pt4

Surface East 31 Facet B, Coating 3

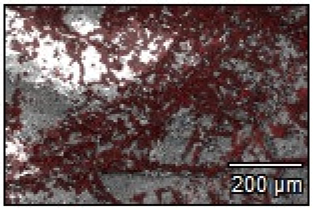

Data Type:	Atomic %
Image Resolution:	512 by 340
Image Pixel Size:	1.62 µm
Map Resolution:	512 by 340
Map Pixel Size:	1.62 µm
Acc. Voltage:	15.0 kV
Magnification:	500

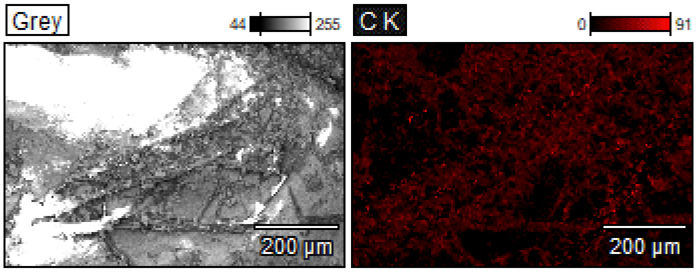

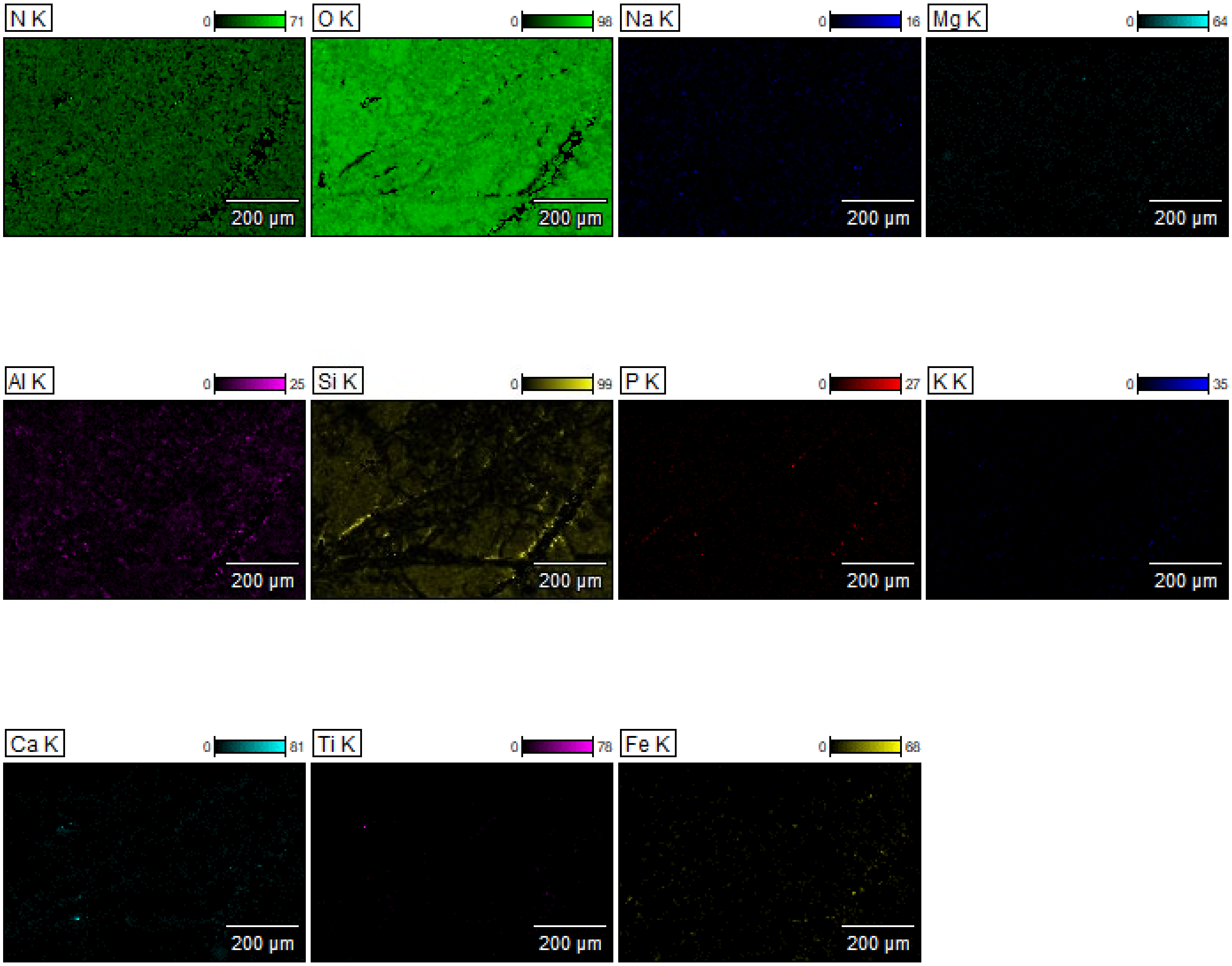

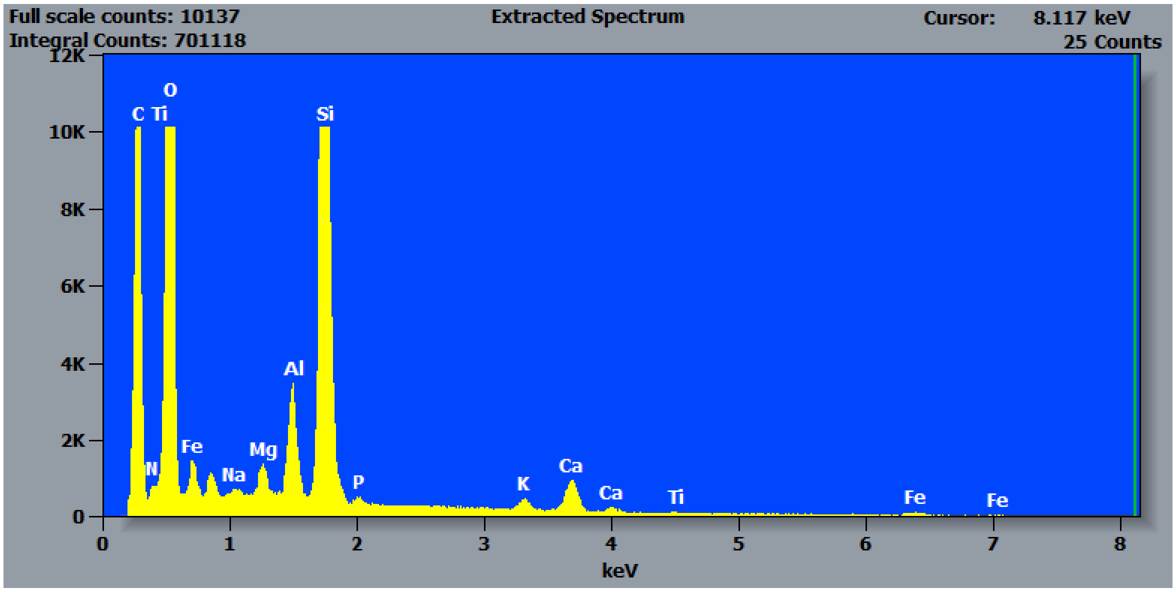

Surface E 31 B (3)						
**Element**	**Net Counts**	**Weight %**	**Weight % err**	**Atom %**	**Norm. Wt. %**	**Chemical Formula**
C K	86588	21.88	0.11	29.81	21.88	C
N K	0	0.00	-	0.00	0.00	N
O K	218963	57.14	0.29	58.45	57.14	O
Na K	0	0.00	-	0.00	0.00	Na
Mg K	5561	0.54	0.02	0.37	0.54	Mg
Al K	24746	2.09	0.03	1.27	2.09	Al
Si K	182103	15.37	0.07	8.96	15.37	Si
P K	2051	0.19	0.01	0.10	0.19	P
K K	3197	0.42	0.01	0.18	0.42	K
Ca K	10345	1.54	0.02	0.63	1.54	Ca
Ti K	530	0.12	0.02	0.04	0.12	Ti
Fe K	1536	0.72	0.07	0.21	0.72	Fe
**% Total**	**-**	**100**	**-**	**100**	**100**	**-**

Surface East 31 Facet B, Coating 4

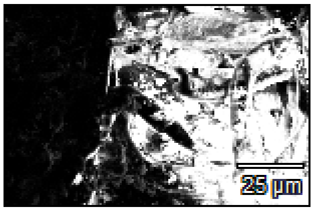

Data Type:	Atomic %
Image Resolution:	512 by 340
Image Pixel Size:	0.23 µm
Map Resolution:	512 by 340
Map Pixel Size:	0.23 µm
Acc. Voltage:	15.0 kV
Magnification:	3497

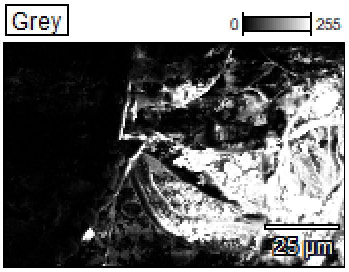

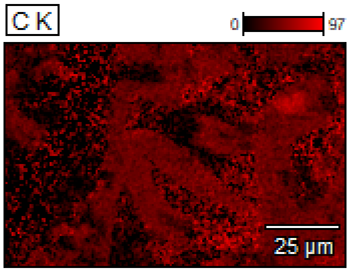

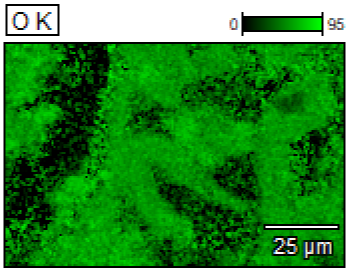

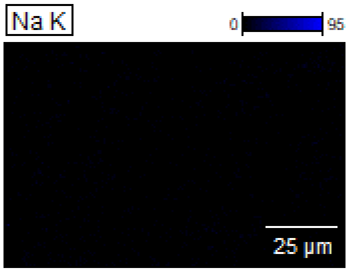

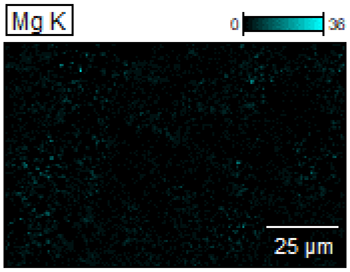

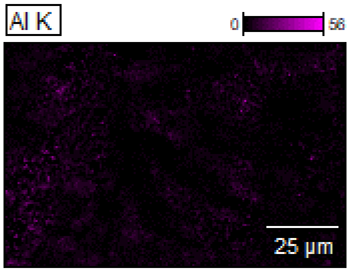

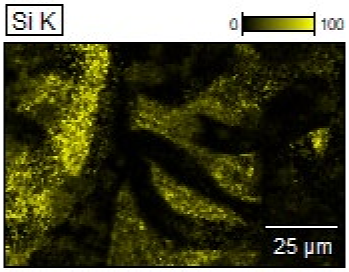

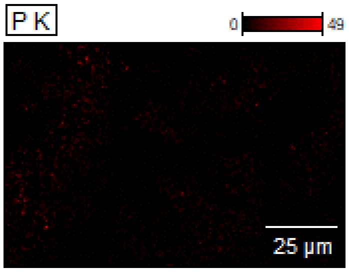

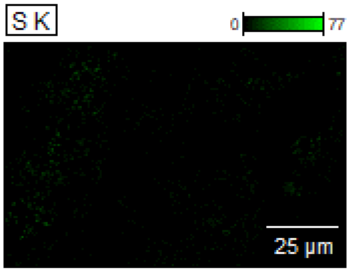

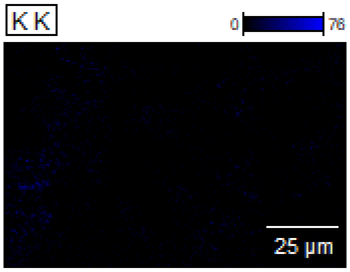

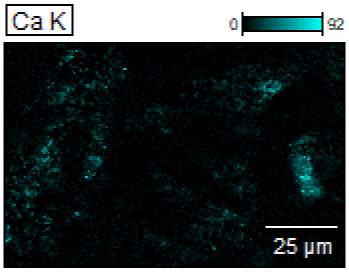

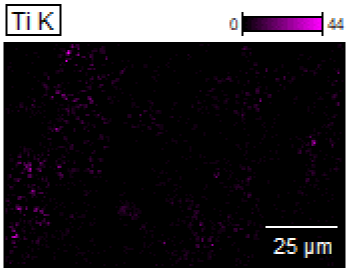

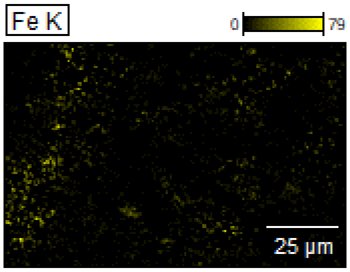

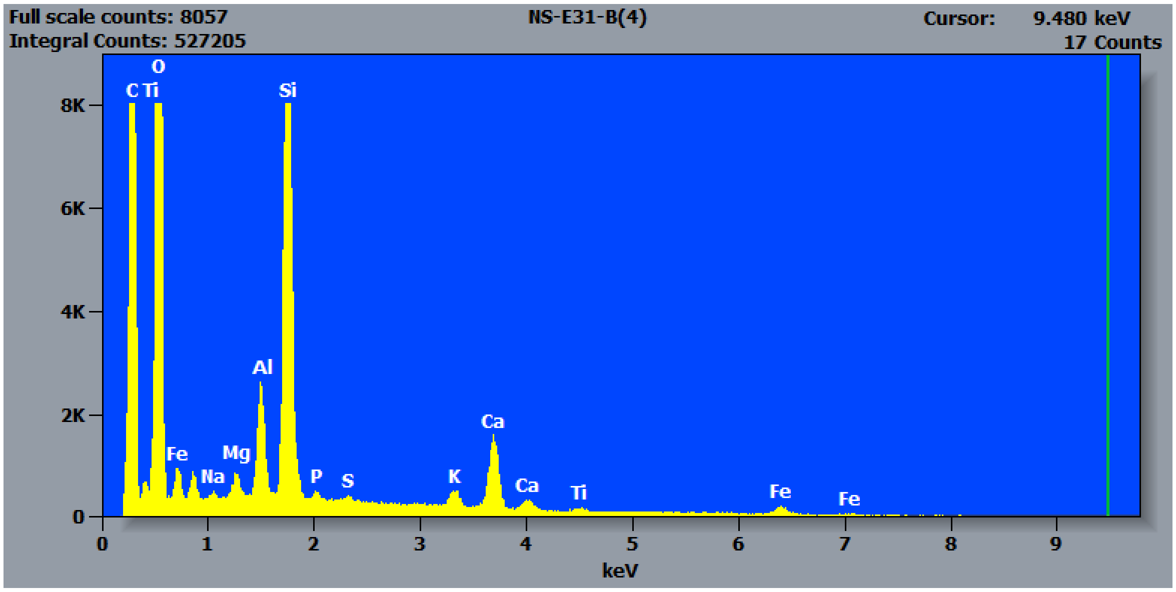

Surface E 31 B(4)						
**Element**	**Net Counts**	**Weight %**	**Weight % err**	**Atom %**	**Norm. Wt. %**	**Chemical Formula**
C K	108619	31.69	0.16	42.00	31.69	C
O K	107387	47.71	0.28	47.47	47.71	O
Na K	0	0.00	-	0.00	0.00	Na
Mg K	3160	0.42	0.03	0.27	0.42	Mg
Al K	18717	2.14	0.03	1.26	2.14	Al
Si K	101714	11.66	0.06	6.61	11.66	Si
P K	1830	0.22	0.03	0.11	0.22	P
S K	1361	0.16	0.01	0.08	0.16	S
K K	3794	0.68	0.02	0.27	0.68	K
Ca K	17200	3.48	0.06	1.38	3.48	Ca
Ti K	1031	0.32	0.05	0.11	0.32	Ti
Fe K	2408	1.54	0.12	0.44	1.54	Fe
**% Total**	**-**	**100**	**-**	**100**	**100**	**-**

Extended Data Figure 8. Selected photographs of natural residue on stone blanks.

Surface stone, no. 31: Microlaminated, interstitial precipitates trapping biogenic clasts.

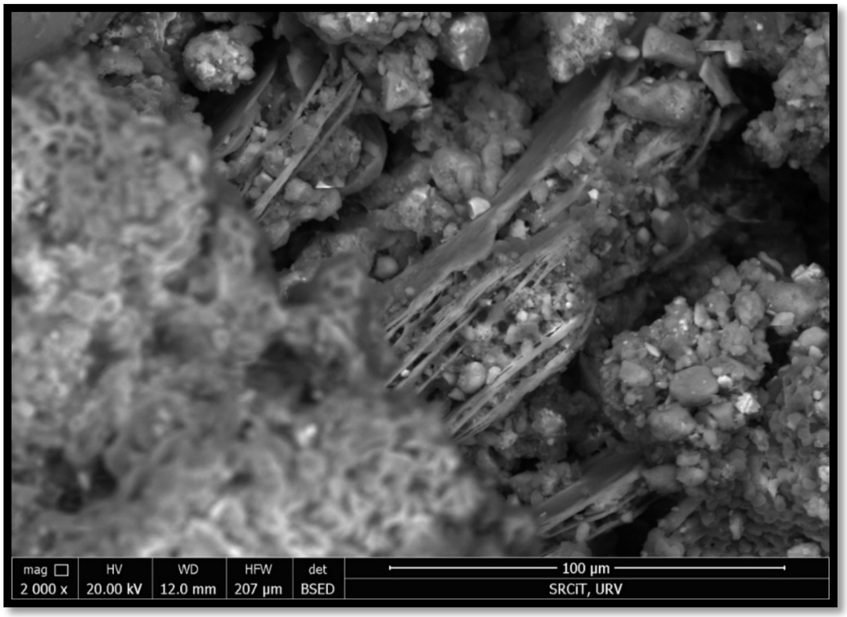

Surface stone, no. 31: Lichen infrastructure supporting spores and phytoliths.

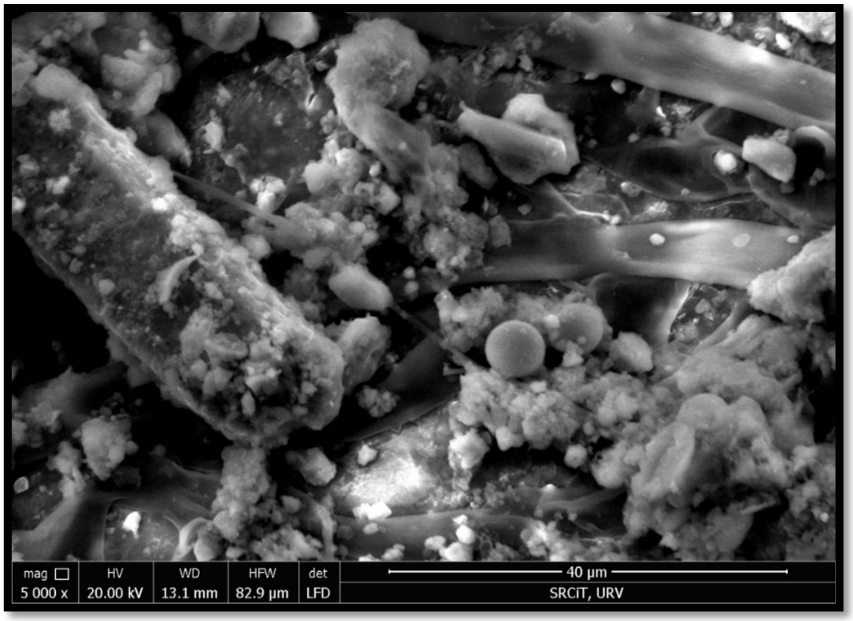

Surface stone, no. 31: Microbial aggregates/lithobiont

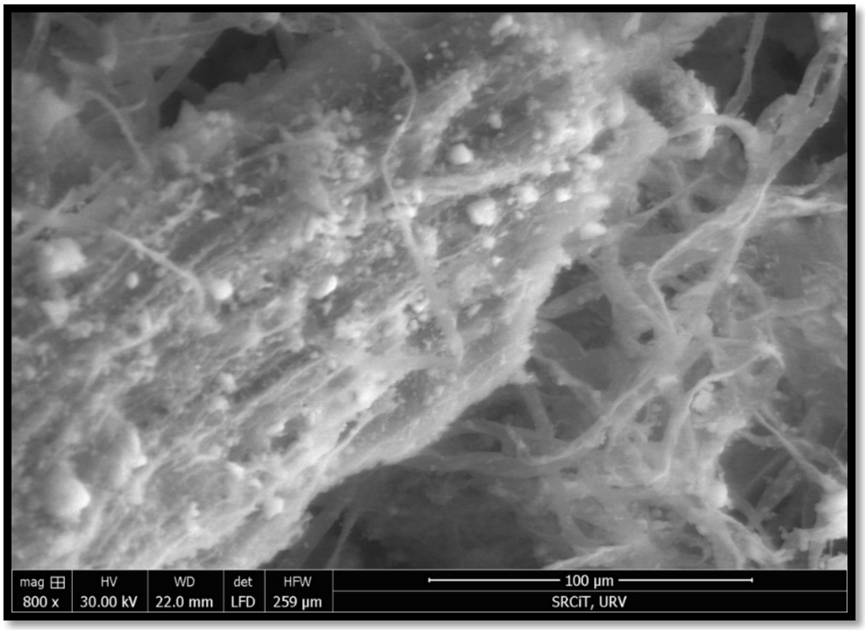

Surface stone no. 17: Lichen microcosm: spores and diatoms.

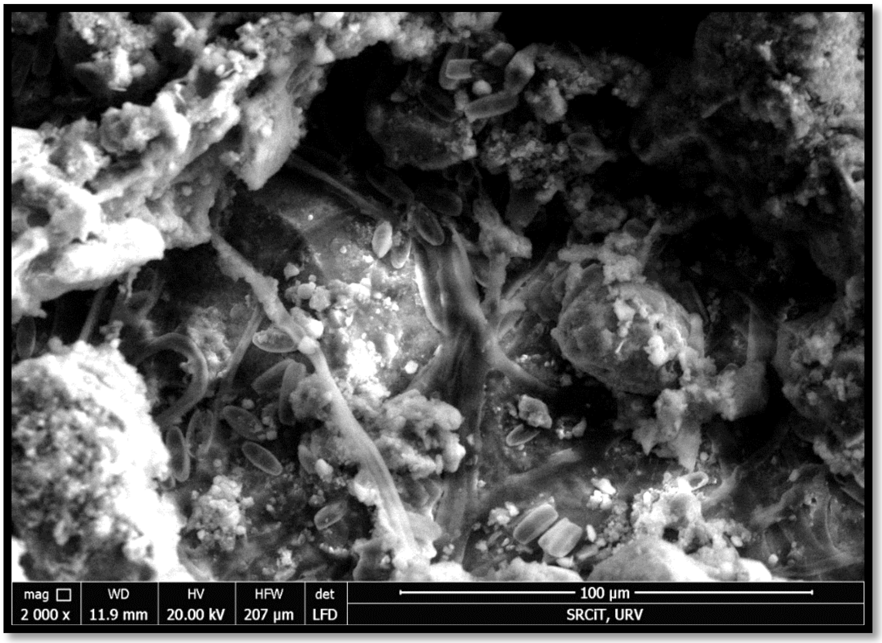

Surface stone no. 17: Phytolith, grass bilobate, commom morphotype at Oldupai Gorge.

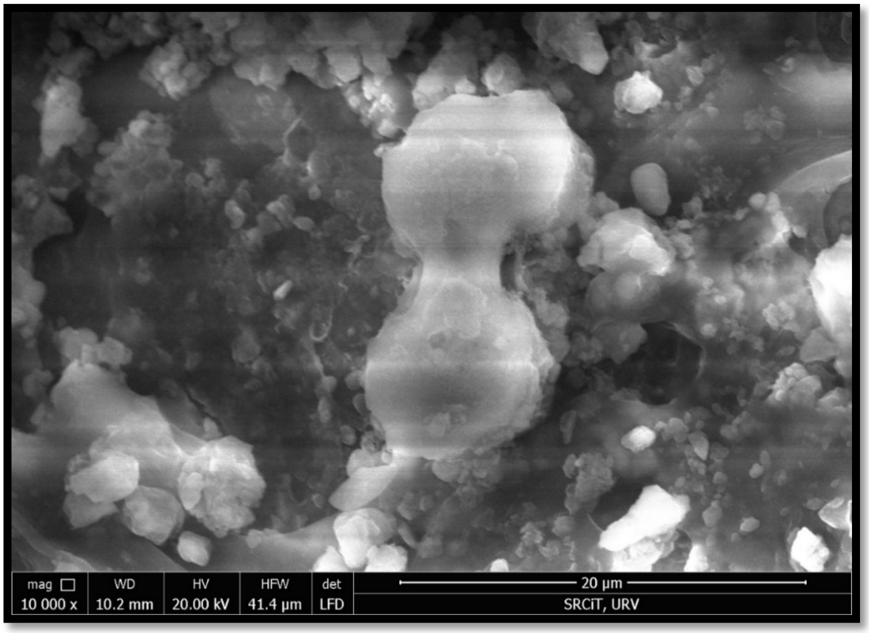

Surface stone, no. 31: Cemented matrix inclusive of geological and biogenic clasts, including epidermal tissue.

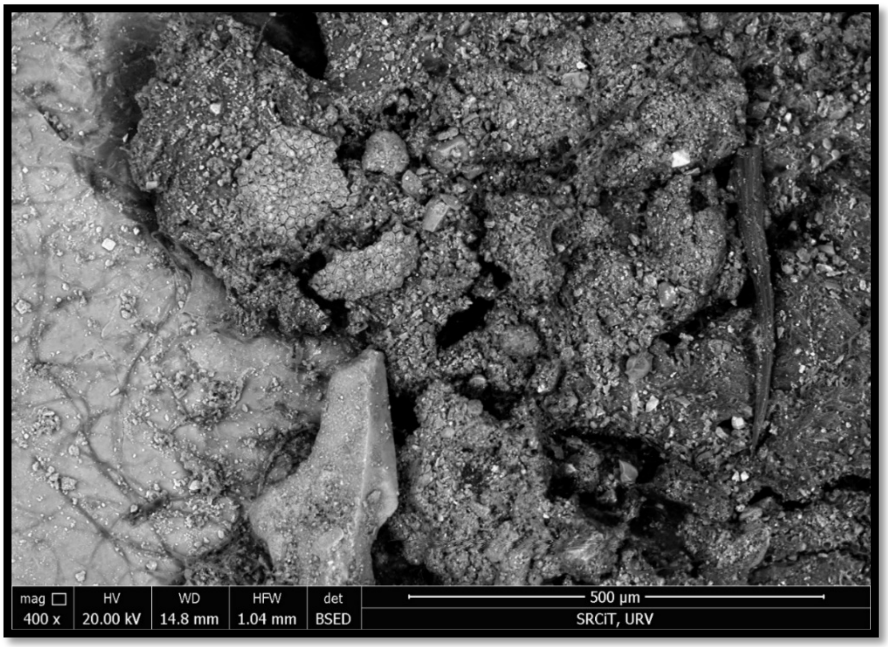

Surface stone E31: Phytolith, tabular shape common at Oldupai Gorge.

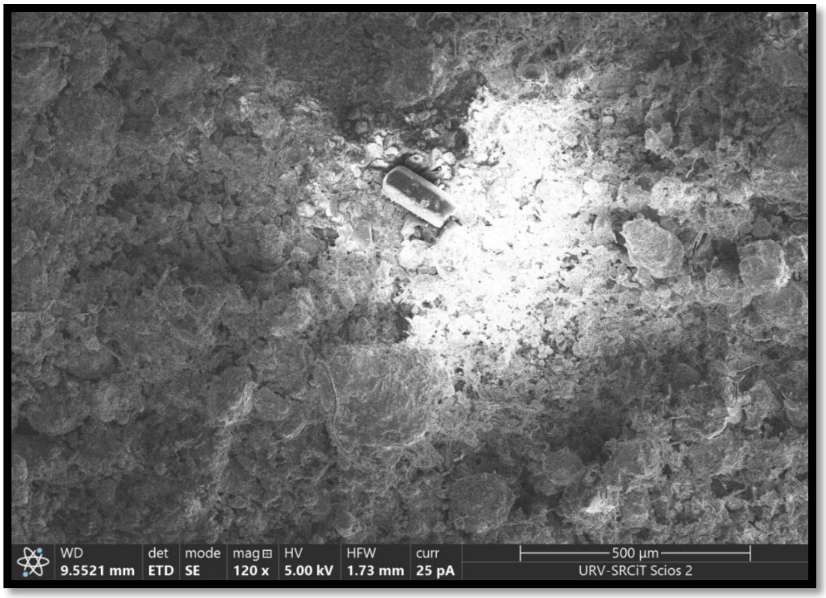

Surface stone E31: Fissures and voids infilled with sediment concretions and organic particles.

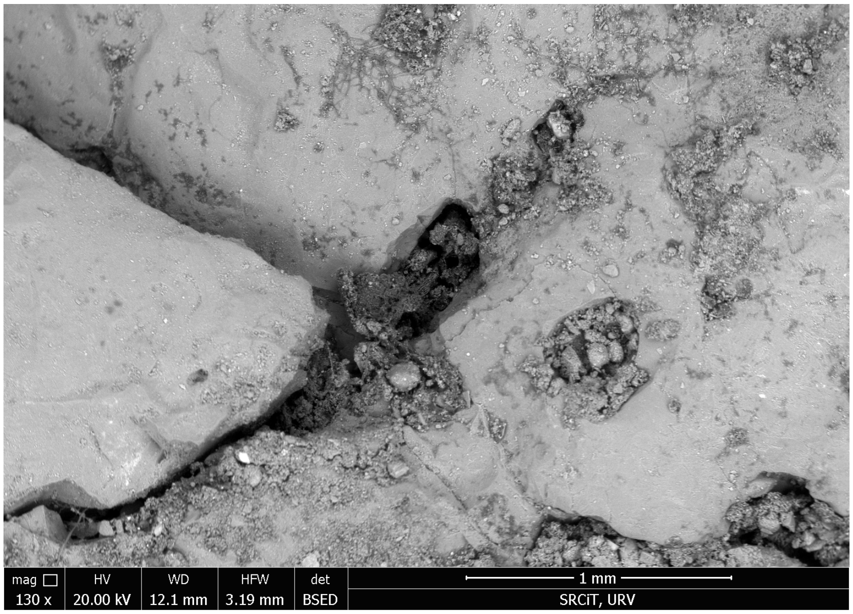

Extended Data Figure 9. Selected images of phytoliths, starch granules, non-pollen palynomorphs, and spicules from surface stones.

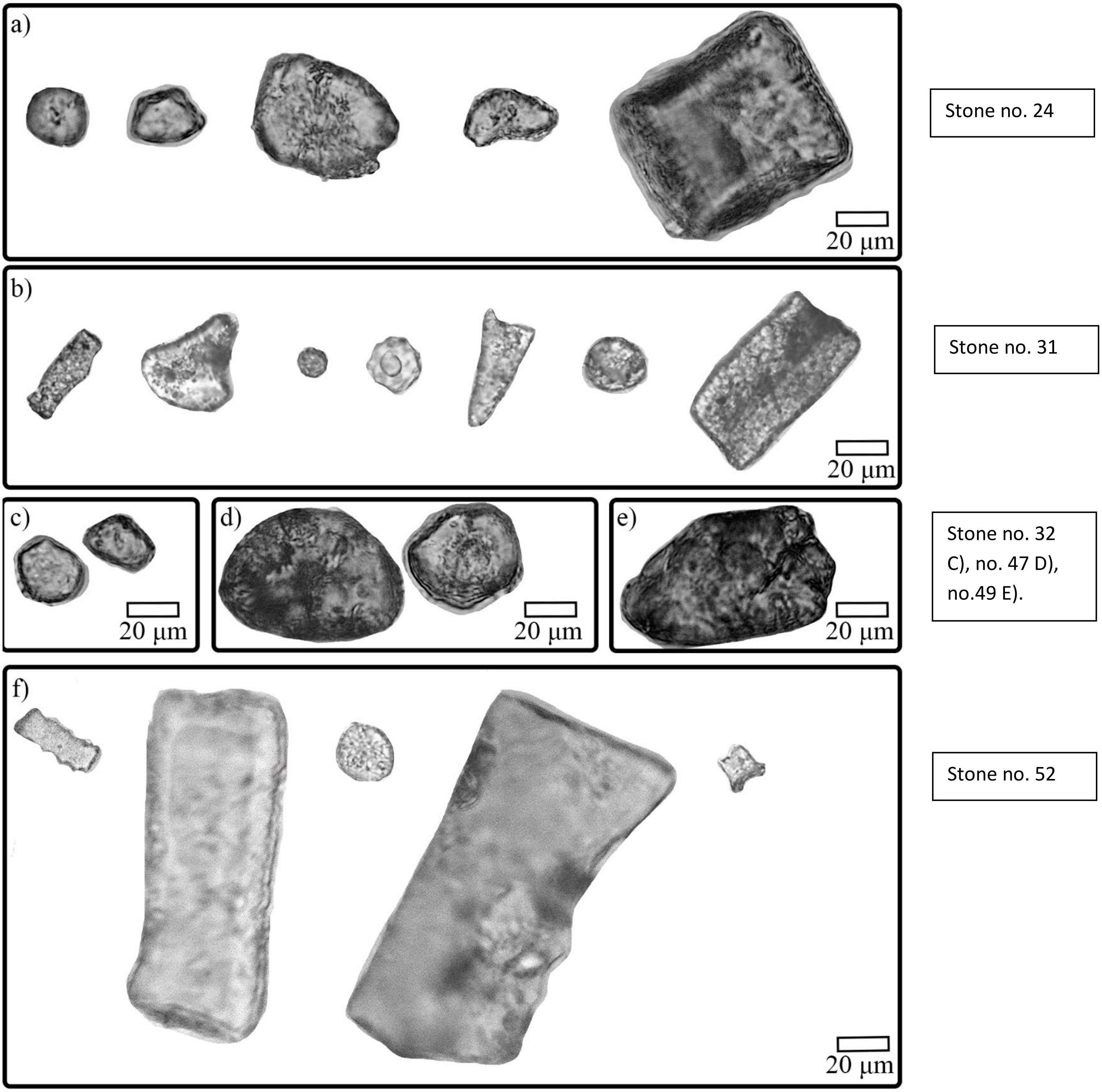

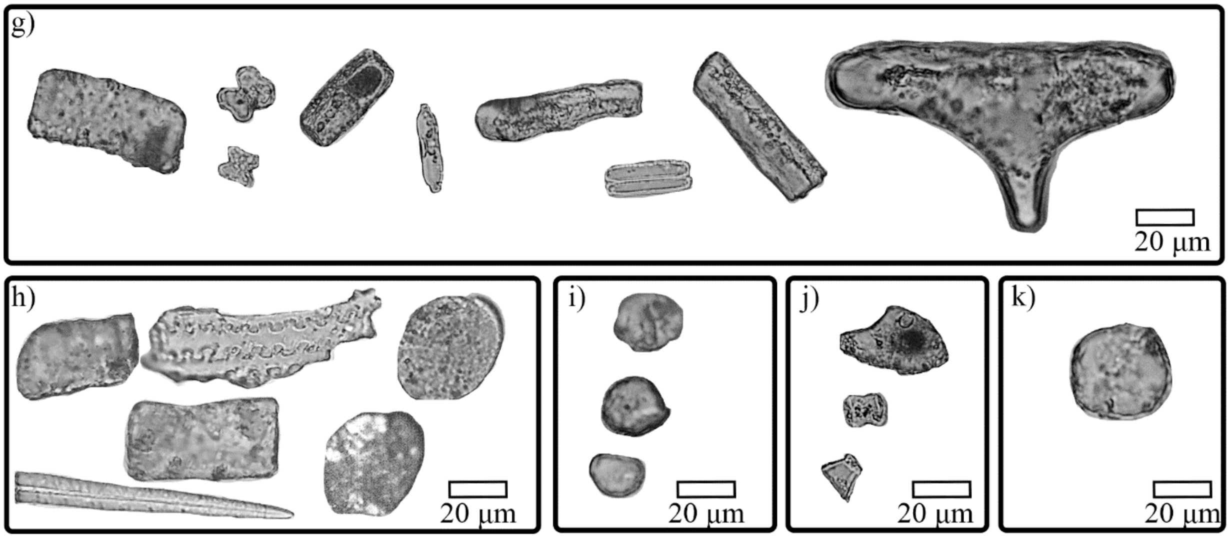

Stone no. 60

Stone no. 70

Stone no. 2

Stone no. 7

Stone no. 67

Extended Data Figure 10. Subsurface stone no. 8,

Facet A, Coating 1

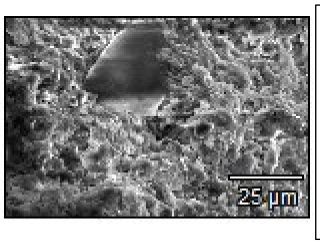

Data Type:	Atomic %
Image Resolution:	512 by 340
Image Pixel Size:	0.20 μm
Map Resolution:	512 by 340
Map Pixel Size:	0.20 μm
Acc. Voltage:	15.0 kV
Magnification:	3996

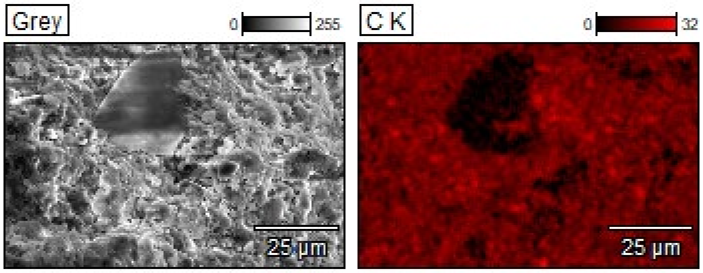

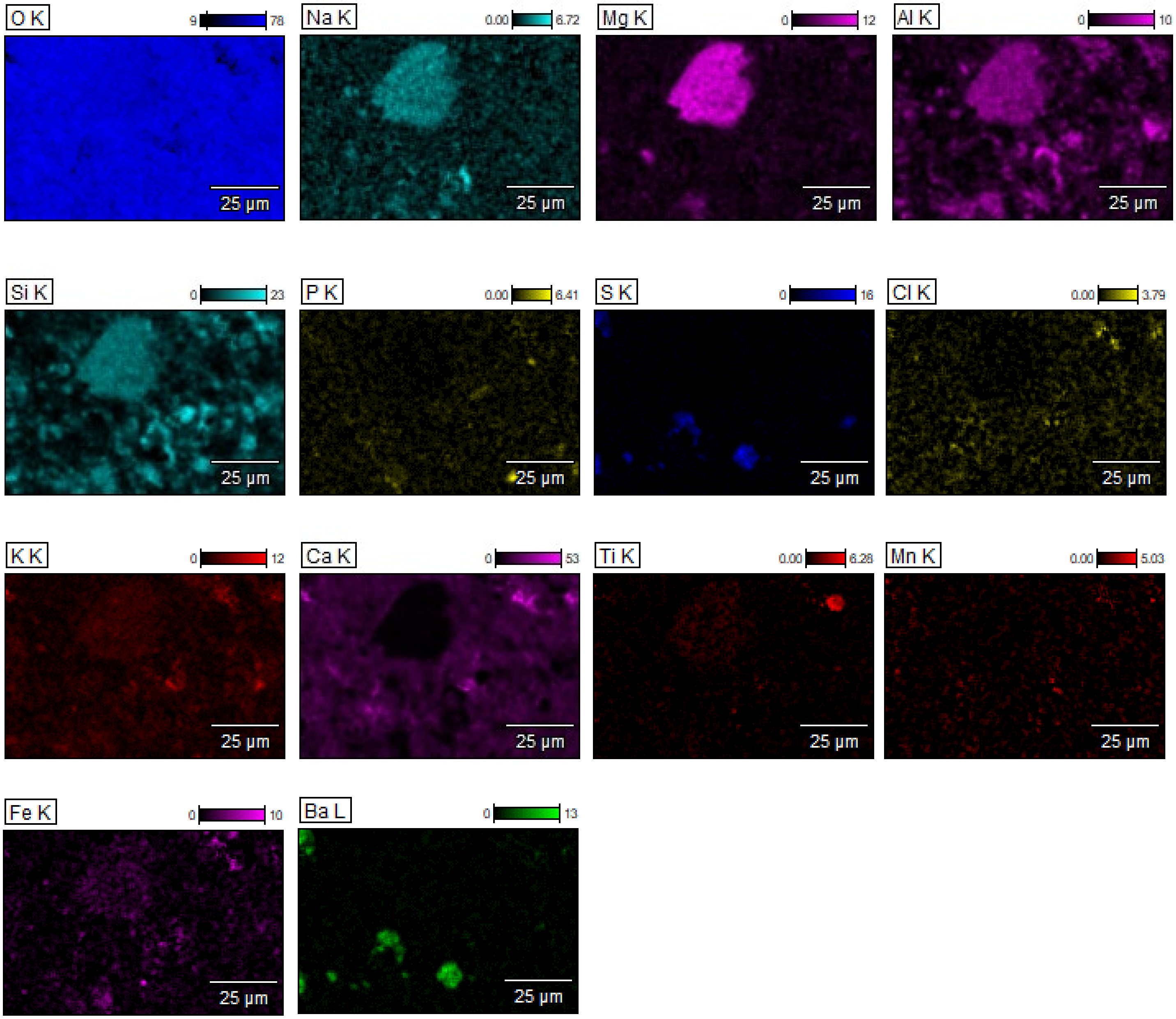

Subsurface 8 (1)						
**Element**	**Net Counts**	**Weight %**	**Weight % err**	**Atom %**	**Norm. Wt. %**	**Chemical Formula**
C K	159203	10.37	0.05	16.42	10.37	C
O K	497903	54.20	0.28	64.44	54.20	O
Na K	14496	0.66	0.01	0.55	0.66	Na
Mg K	58739	2.02	0.02	1.58	2.02	Mg
Al K	80493	2.40	0.02	1.69	2.40	Al
Si K	219700	6.44	0.03	4.36	6.44	Si
P K	14714	0.43	0.01	0.26	0.43	P
S K	19345	0.56	0.01	0.33	0.56	S
Cl K	10608	0.37	0.01	0.2	0.37	Cl
K K	62641	2.65	0.02	1.29	2.65	K
Ca K	338502	16.78	0.06	7.96	16.78	Ca
Ti K	6165	0.48	0.04	0.19	0.48	Ti
Mn K	1656	0.23	0.02	0.08	0.23	Mn
Fe K	9535	1.50	0.06	0.51	1.50	Fe
Ba L	7537	0.92	0.09	0.13	0.92	Ba
**% Total**	-	**100**	-	**100**	**100**	-

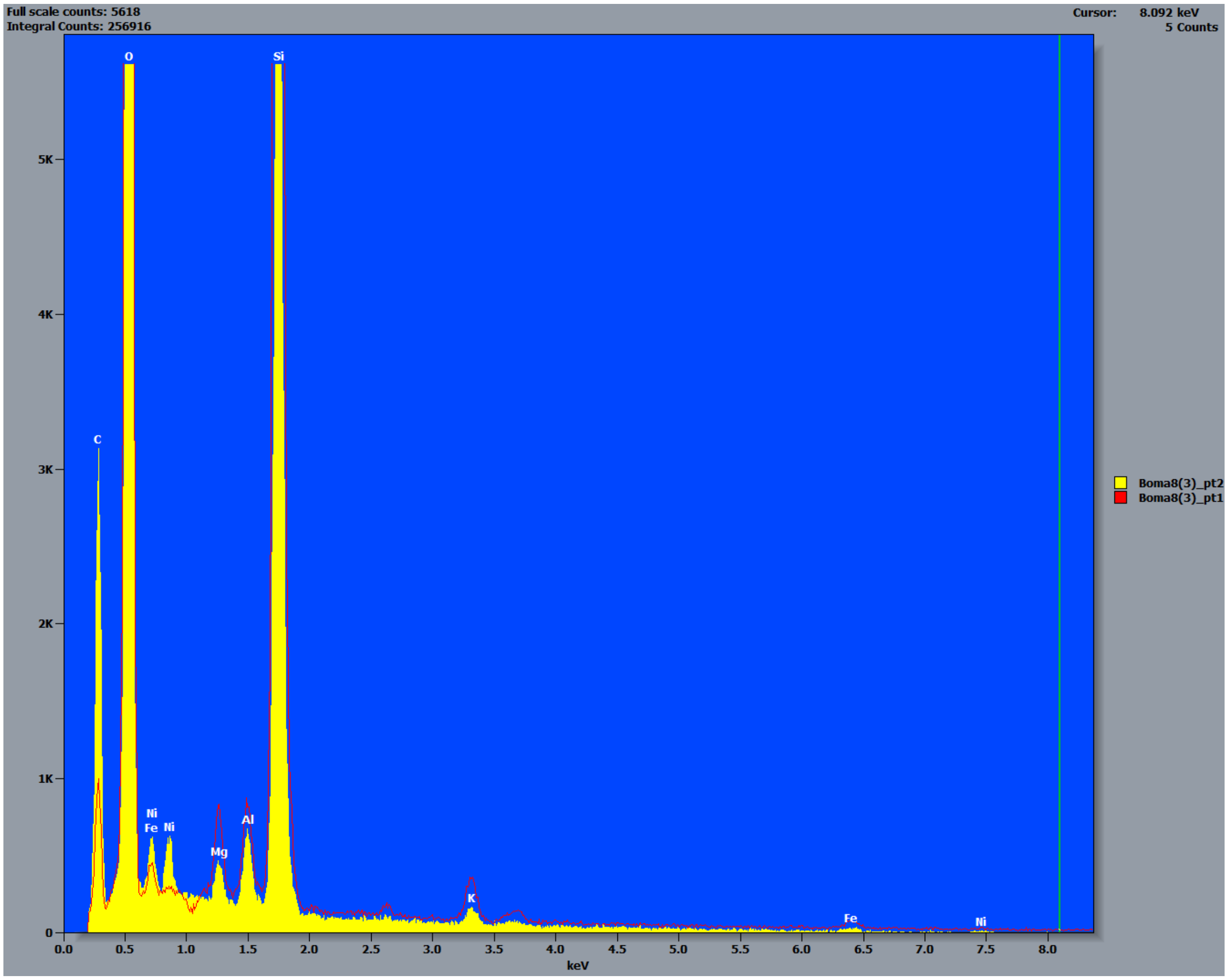

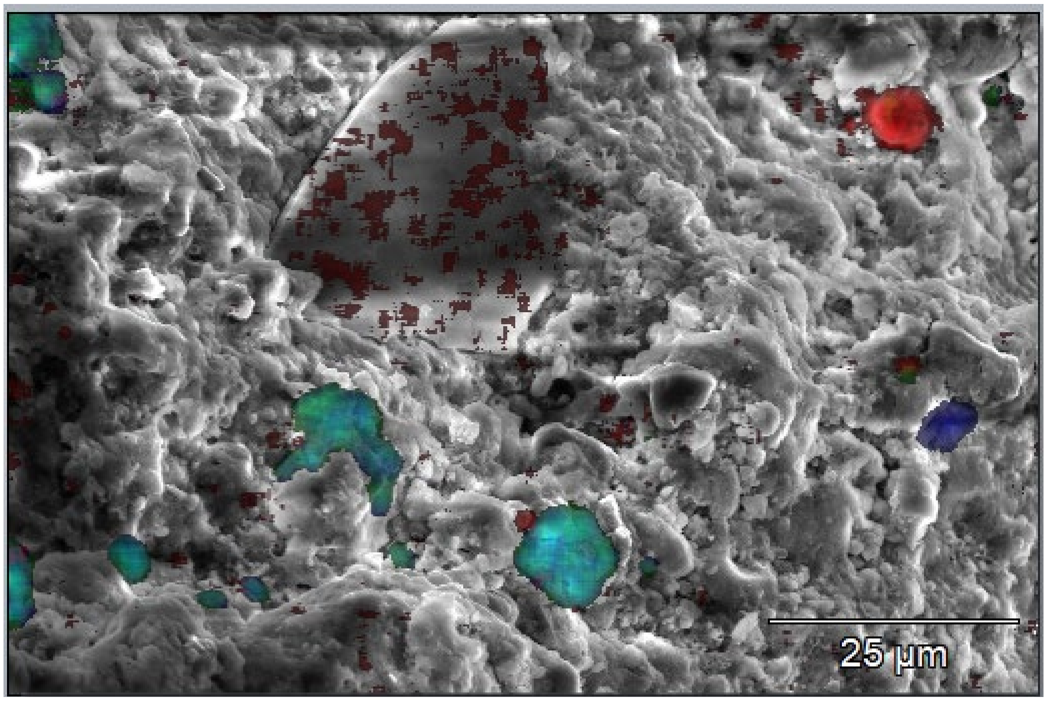
25 µm

Subsurface 8, Facet A, Coating 3

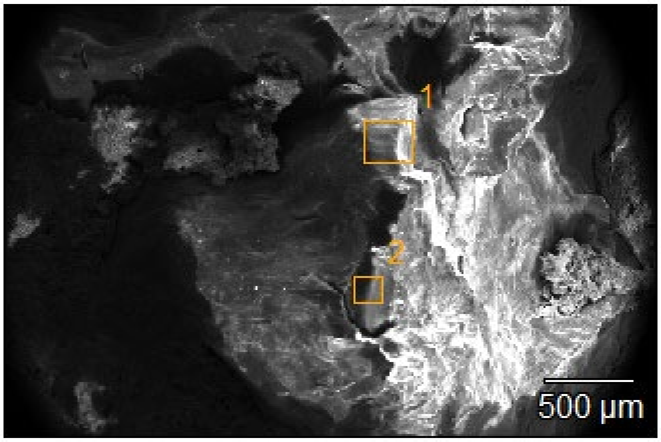

Image Name:	Subsurface 8 (3)
Image Resolution:	512 by 340
Image Pixel Size:	7.23 µm
Acc. Voltage:	15.0 kV
Magnification:	112

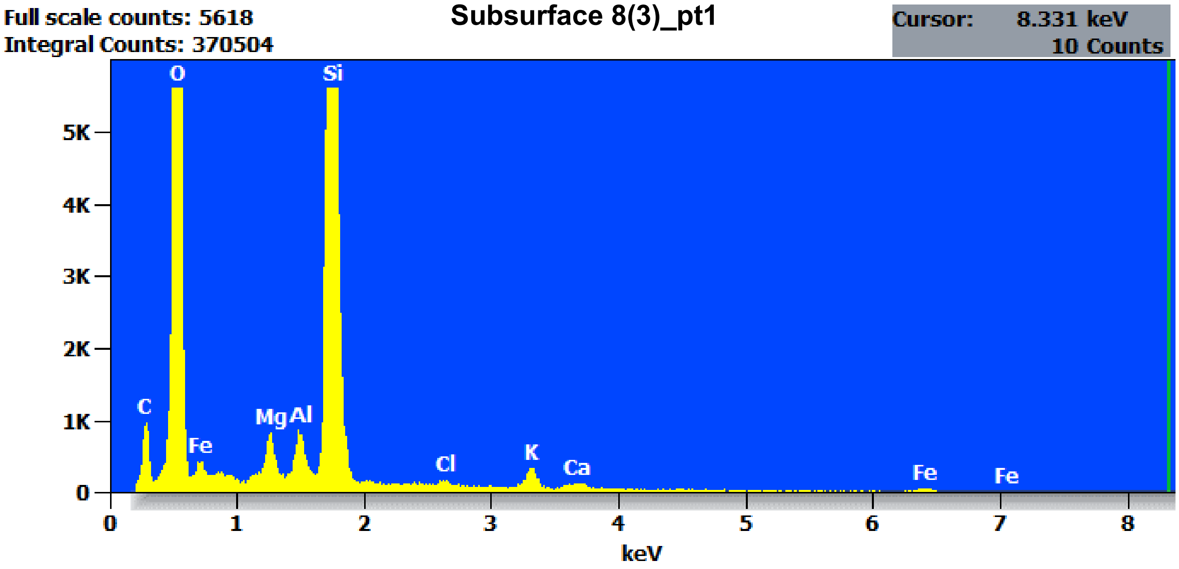

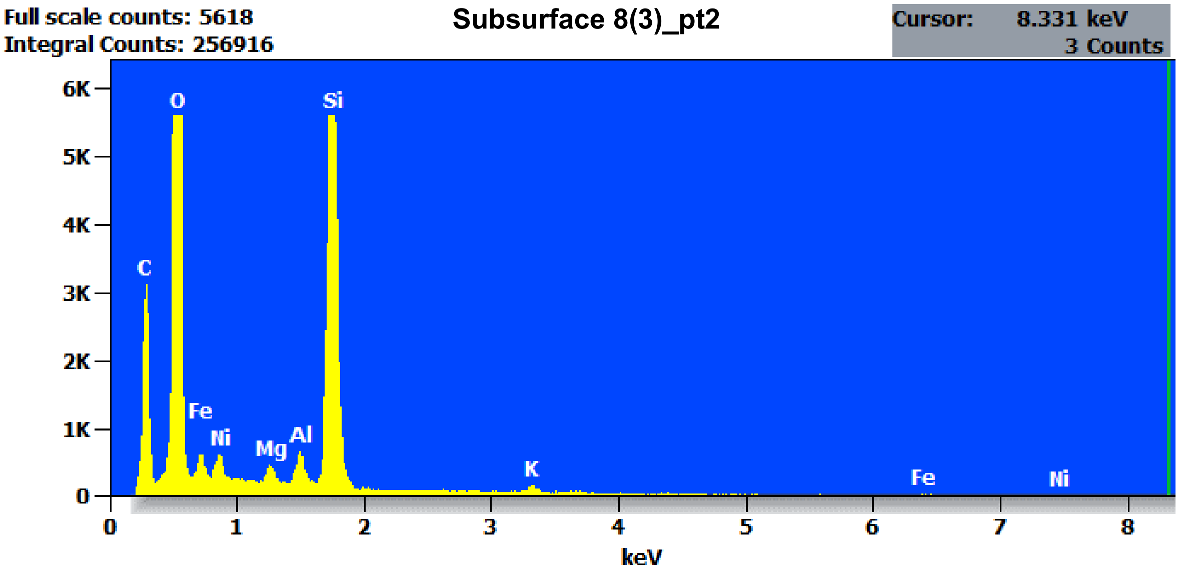

Net Counts										
	C	O	Mg	Al	Si	Cl	K	Ca	Fe	Ni
Subsurface 8 (3)_pt1	4444	130476	4984	4638	148713	738	3480	912	767	-
Subsurface 8 (3)_pt2	17712	97960	2121	3935	70710	-	1360	-	409	268

Weight %										
	C	O	Mg	Al	Si	Cl	K	Ca	Fe	Ni
Subsurface 8 (3)_pt1	3.84	61.84	1.13	0.93	29.71	0.20	1.14	0.34	0.87	-
Subsurface 8 (3)_pt2	14.51	63.38	0.65	1.04	18.63	-	0.57	-	0.60	0.62

Weight % Error (+/-1 Sigma)										
	C	O	Mg	Al	Si	Cl	K	Ca	Fe	Ni
Subsurface 8 (3)_pt1	±0.07	±0.32	±0.03	±0.04	±0.12	±0.02	±0.05	±0.02	±0.13	-
Subsurface 8 (3)_pt2	±0.11	±0.37	±0.02	±0.03	±0.10	-	±0.02	-	±0.06	±0.12

Normalized Wt. %										
	C	O	Mg	Al	Si	Cl	K	Ca	Fe	Ni
Subsurface 8 (3)_pt1	3.84	61.84	1.13	0.93	29.71	0.2	1.14	0.34	0.87	-
Subsurface 8 (3)_pt2	14.51	63.38	0.65	1.04	18.63	-	0.57	-	0.6	0.62

Atom %										
	C	O	Mg	Al	Si	Cl	K	Ca	Fe	Ni
Subsurface 8 (3)_pt1	5.94	71.81	0.87	0.64	19.65	0.11	0.54	0.16	0.29	-
Subsurface 8 (3)_pt2	20.36	66.76	0.45	0.65	11.18	-	0.25	-	0.18	0.18

Formula										
	C	O	Mg	Al	Si	Cl	K	Ca	Fe	Ni
Subsurface 8 (3)_pt1	C	O	Mg	Al	Si	Cl	K	Ca	Fe	-
Subsurface 8 (3)_pt2	C	O	Mg	Al	Si	-	K	-	Fe	Ni

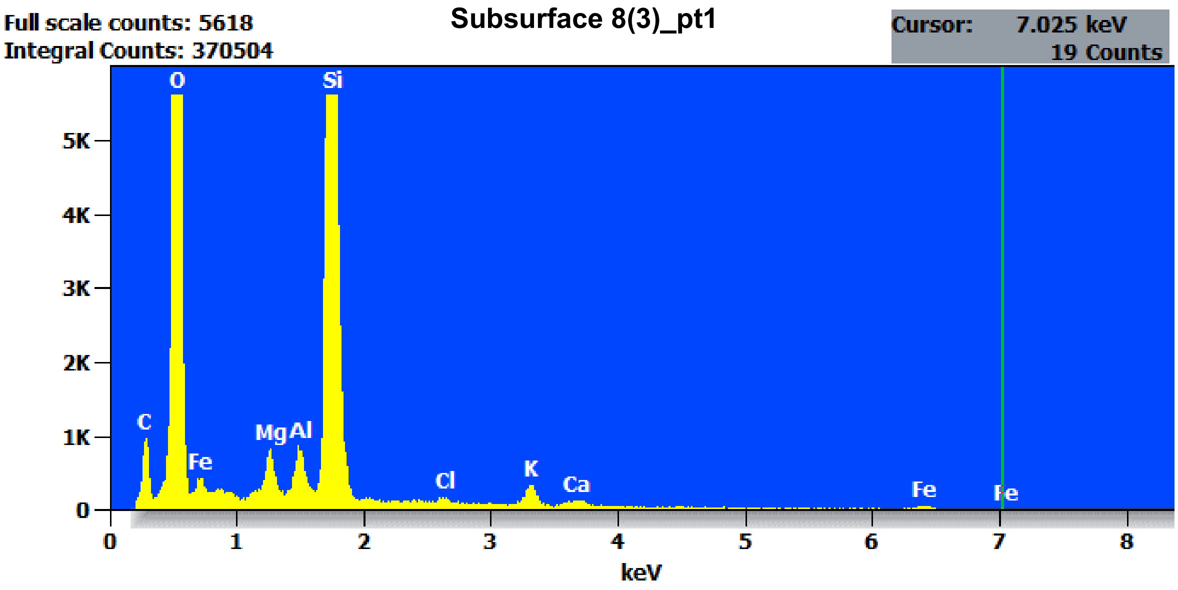

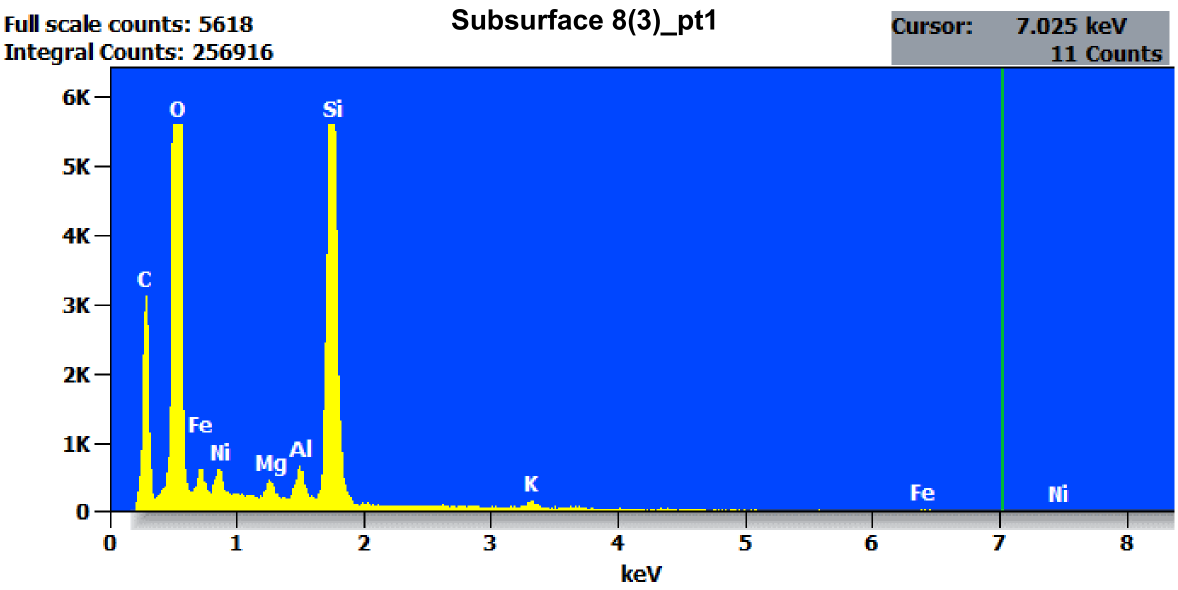
Full scale counts: 5618
Integral Counts: 256916
Subsurface 8(3)_pt1
Cursor: 7.025 keV
11 Counts
6K
5K
4K
3K
2K
1K
0
O
Si
C
Fe
Ni
Mg
Al
K
Fe
Ni
0
1
2
3
4
5
6
7
8
keV

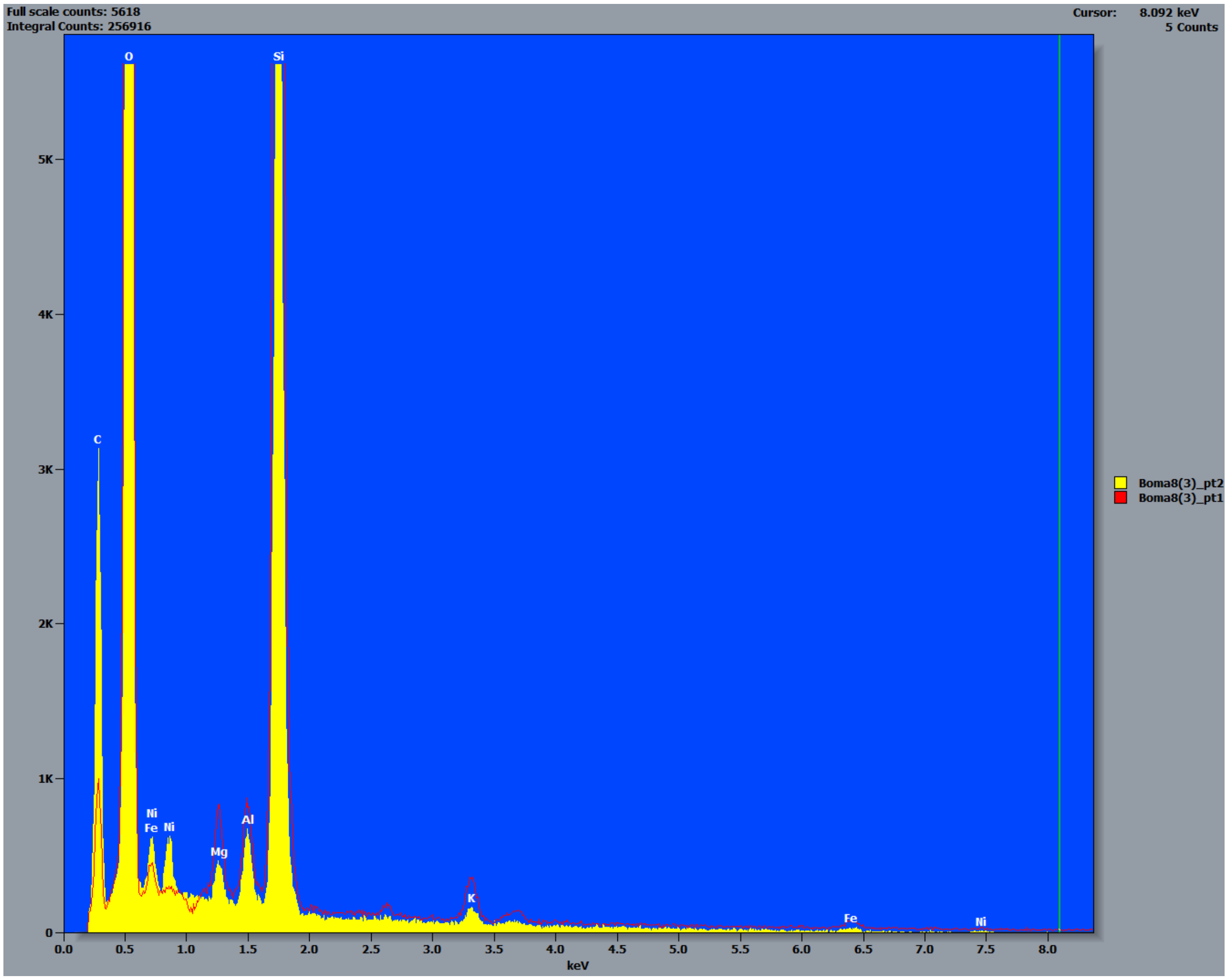
Full scale counts: 5618
Integral Counts: 256916
Cursor: 8.092 keV
5 Counts
5K
4K
3K
2K
1K
0
O
Si
C
Ni
Fe
Ni
Mg
Al
K
Fe
Ni
Boma8(3)_pt2
Boma8(3)_pt1
0.0
0.5
1.0
1.5
2.0
2.5
3.0
3.5
4.0
4.5
5.0
5.5
6.0
6.5
7.0
7.5
8.0
keV

Subsurface 8, Facet A, Coating 4

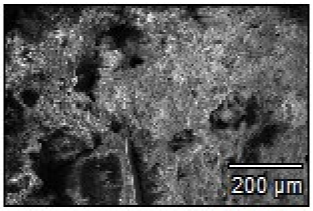

Data Type:	Atomic %
Image Resolution:	512 by 340
Image Pixel Size:	1.62 µm
Map Resolution:	512 by 340
Map Pixel Size:	1.62 µm
Acc. Voltage:	15.0 kV
Magnification:	500

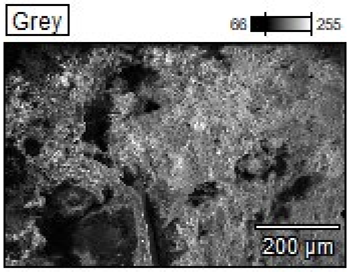

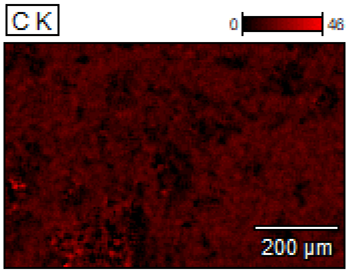

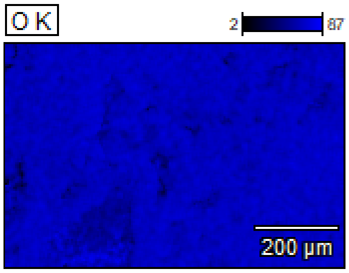

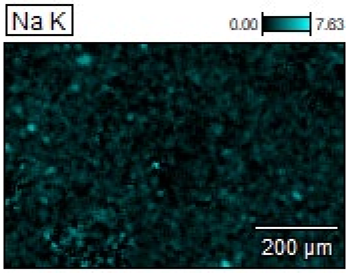

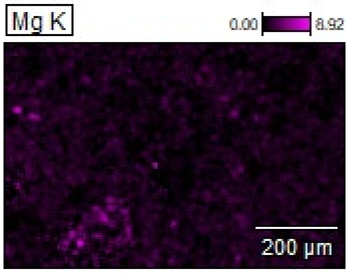

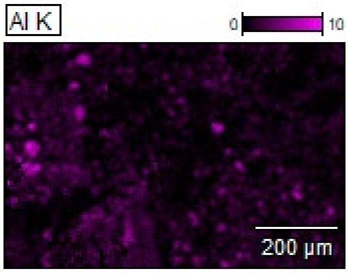

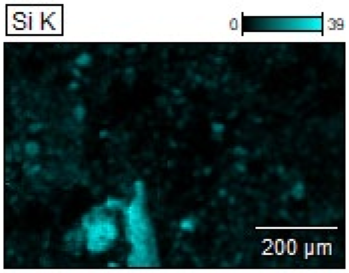

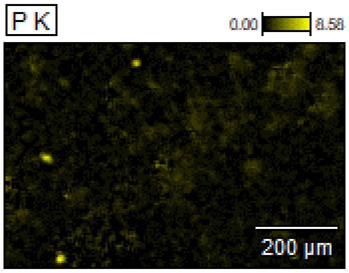

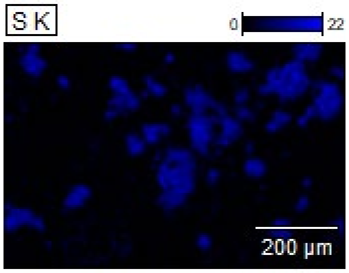

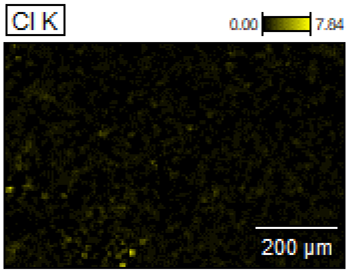

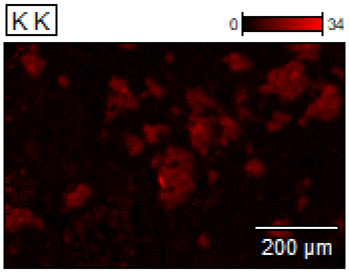

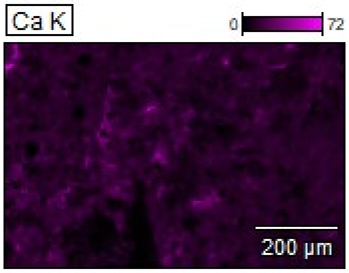

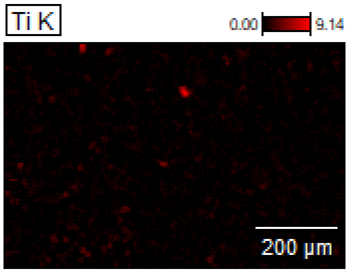

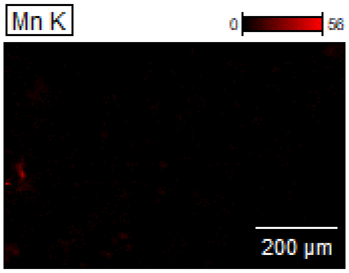

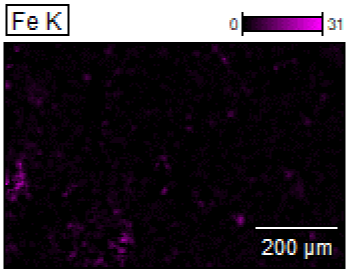

Subsurface 8 (4)						
**Element**	**Net Counts**	**Weight %**	**Weight % err**	**Atom %**	**Norm. Wt. %**	**Chemical Formula**
Fe K	8080	1.59	0.07	0.57	1.59	Fe
Mn K	1658	0.29	0.03	0.10	0.29	Mn
Ti K	3700	0.37	0.03	0.15	0.37	Ti
Ca K	344276	21.52	0.09	10.67	21.52	Ca
K K	99702	5.25	0.04	2.67	5.25	K
Cl K	7879	0.35	0.02	0.19	0.35	Cl
S K	87971	3.10	0.02	1.92	3.10	S
P K	11458	0.40	0.02	0.26	0.40	P
Si K	125735	4.52	0.03	3.20	4.52	Si
Al K	32246	1.19	0.02	0.88	1.19	Al
Mg K	19855	0.86	0.01	0.70	0.86	Mg
Na K	10222	0.59	0.02	0.51	0.59	Na
O K	315848	50.89	0.25	63.18	50.89	O
C K	112985	9.07	0.05	15.00	9.07	C
**% Total**	-	**100**	-	**100**	**100**	-

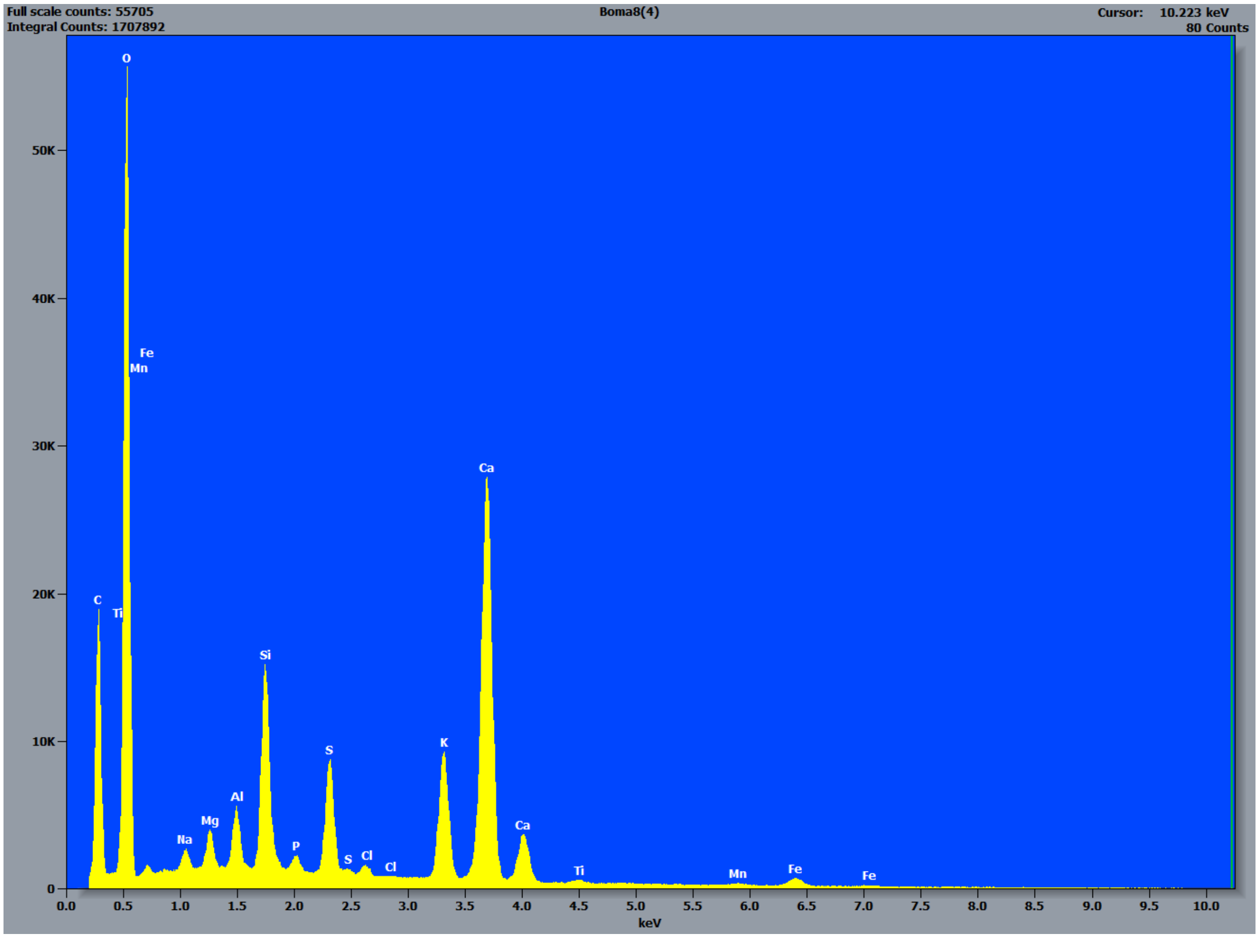

Subsurface 8, Facet A, Coating 5

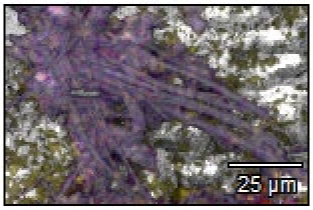

Data Type:	Atomic %
Image Resolution:	512 by 340
Image Pixel Size:	0.20 µm
Map Resolution:	512 by 340
Map Pixel Size:	0.20 µm
Acc. Voltage:	15.0 kV
Magnification:	3996

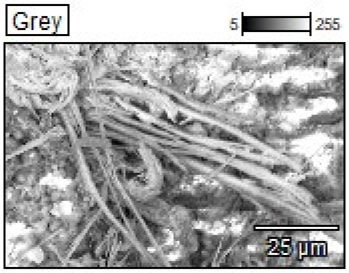

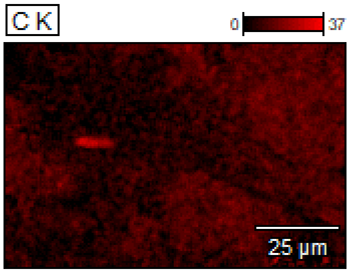

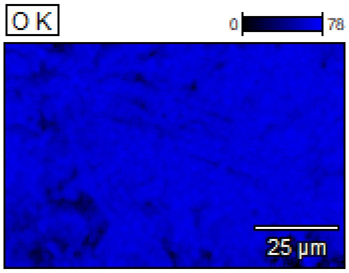

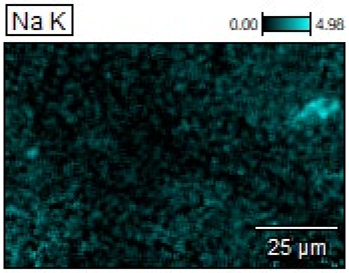

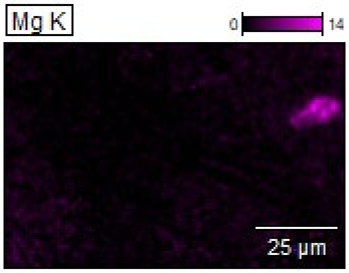

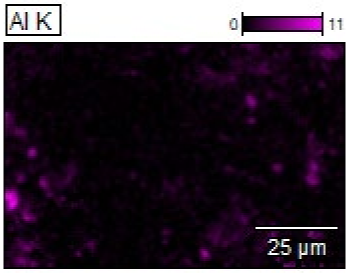

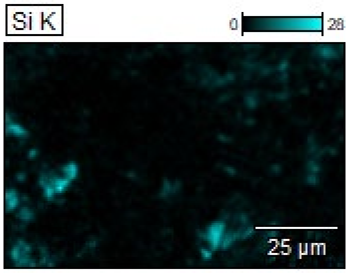

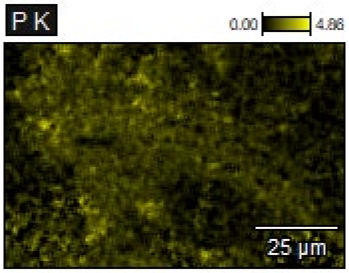

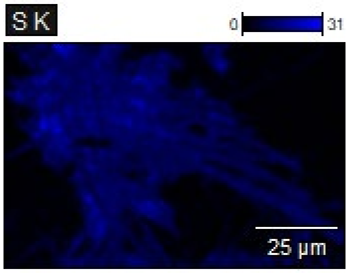

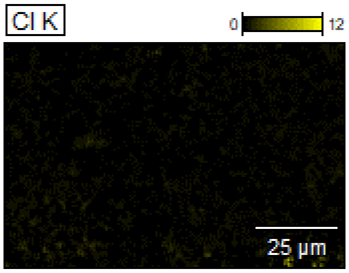

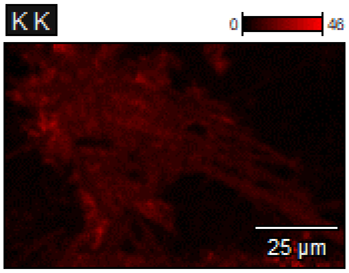

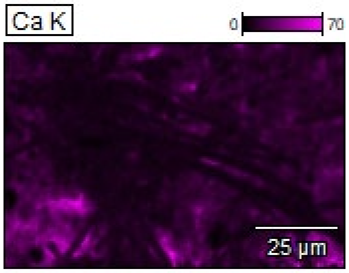

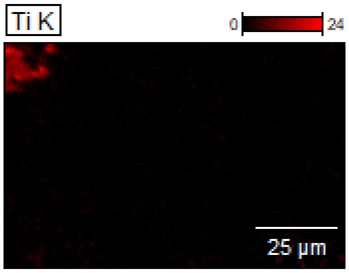

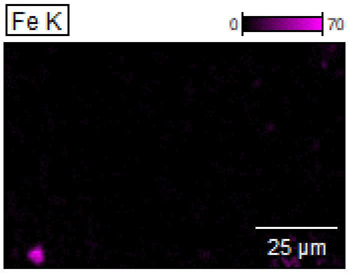

Subsurface 8 (5)						
**Element**	**Net Counts**	**Weight %**	**Weight % err**	**Atom %**	**Norm. Wt. %**	**Chemical Formula**
C K	87212	7.10	0.04	12.27	7.10	C
O K	302490	48.33	0.24	62.67	48.33	O
Na K	6017	0.31	0.02	0.28	0.31	Na
Mg K	17446	0.67	0.02	0.57	0.67	Mg
Al K	16478	0.54	0.02	0.41	0.54	Al
Si K	58991	1.86	0.02	1.37	1.86	Si
P K	9309	0.28	0.02	0.19	0.28	P
S K	276577	8.44	0.03	5.46	8.44	S
Cl K	8618	0.34	0.01	0.20	0.34	Cl
K K	226295	10.68	0.06	5.67	10.68	K
Ca K	353315	19.90	0.09	10.30	19.90	Ca
Ti K	6406	0.57	0.03	0.24	0.57	Ti
Fe K	5685	0.99	0.06	0.37	0.99	Fe
**% Total**	-	**100**	-	**100**	**100**	-

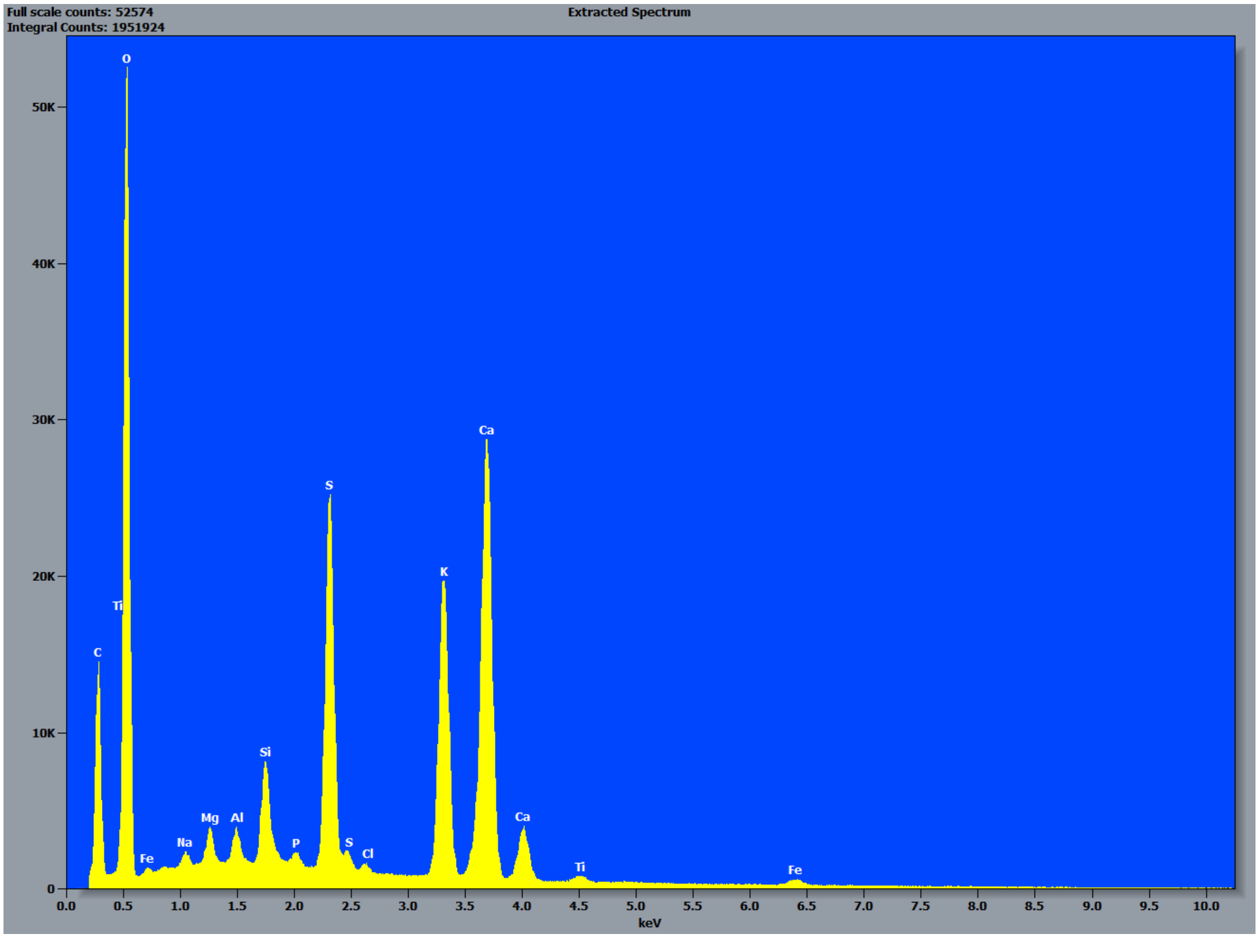

Subsurface 8, Facet A, Coating 6

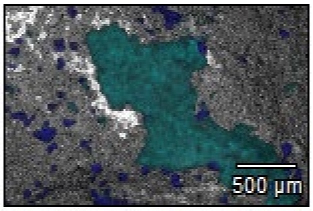

Data Type:	Atomic %
Image Resolution:	512 by 340
Image Pixel Size:	5.06 µm
Map Resolution:	512 by 340
Map Pixel Size:	5.06 µm
Acc. Voltage:	15.0 kV
Magnification:	160

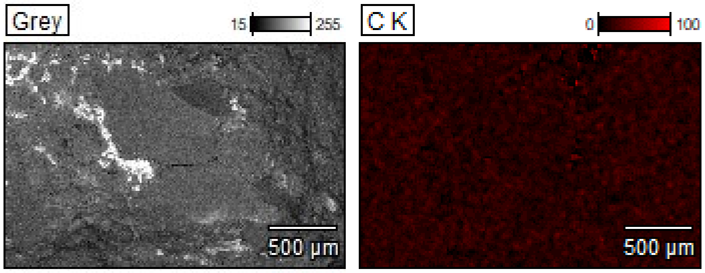

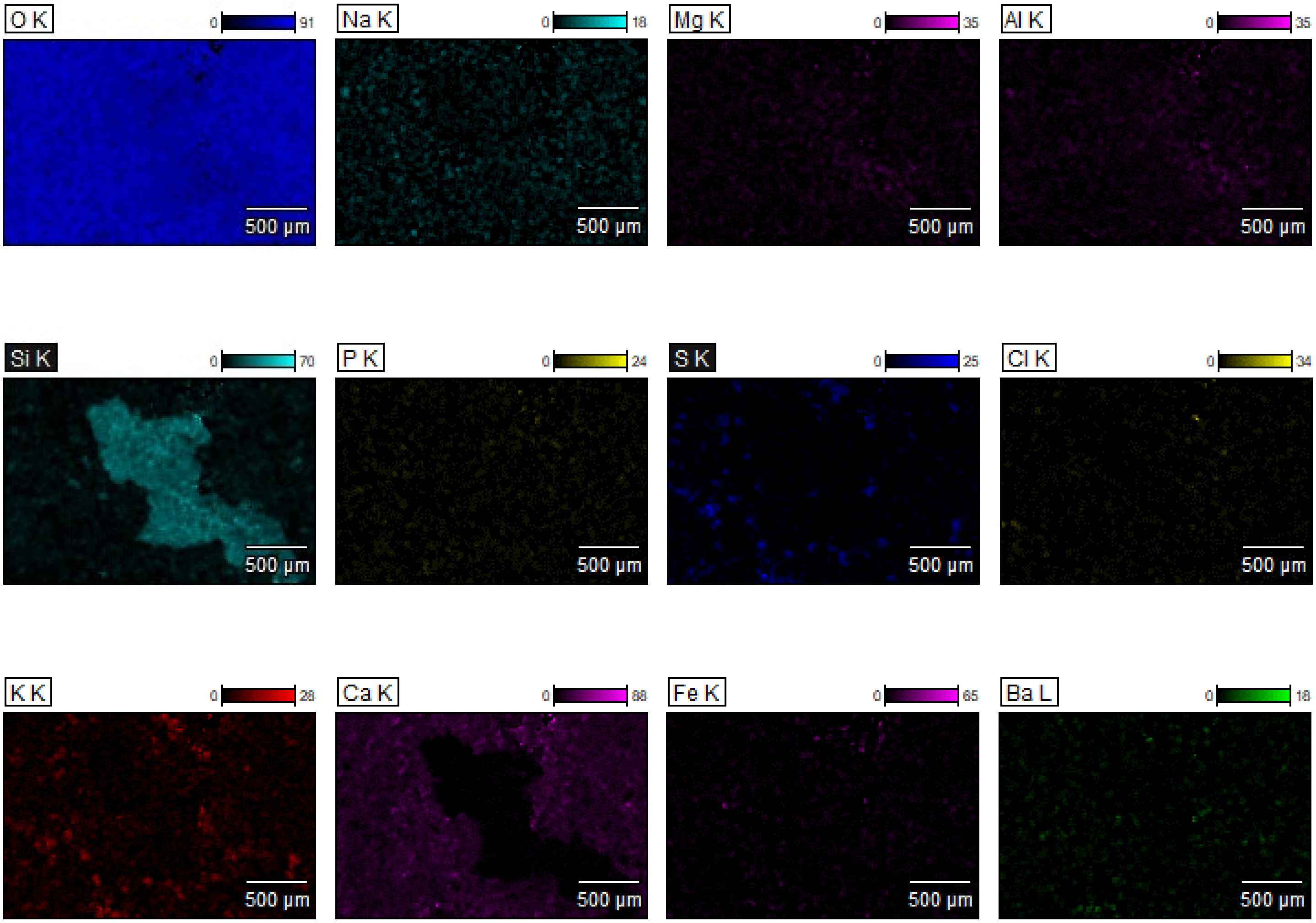

Subsurface 8 (6)

Element	Net Counts	Weight %	Weight % err	Atom %	Norm. Wt. %	Chemical Formula
C K	13270	8.84	0.08	14.55	8.84	C
O K	51778	49.97	0.33	61.74	49.97	O
Na K	1651	0.59	0.03	0.51	0.59	Na
Mg K	4787	1.28	0.03	1.04	1.28	Mg
Al K	7246	1.66	0.03	1.21	1.66	Al
Si K	55032	12.27	0.08	8.63	12.27	Si
P K	1693	0.39	0.02	0.25	0.39	P
S K	5435	1.23	0.02	0.76	1.23	S
Cl K	1702	0.47	0.04	0.26	0.47	Cl
K K	10460	3.42	0.04	1.73	3.42	K
Ca K	45792	17.46	0.10	8.61	17.46	Ca
Fe K	1407	1.68	0.17	0.59	1.68	Fe
Ba L	781	0.73	0.09	0.11	0.73	Ba
**% Total**	-	**100**	-	**100**	**100**	-

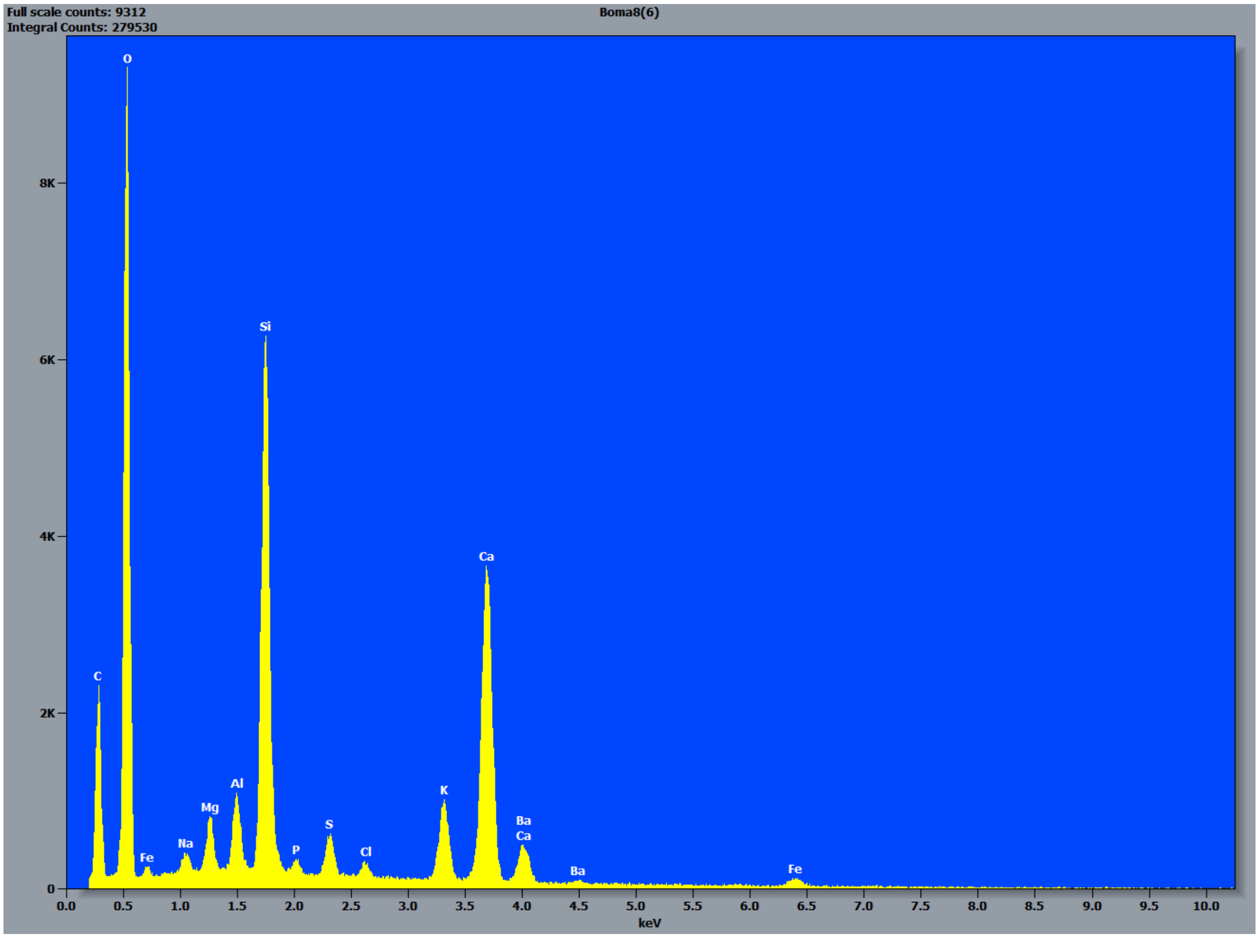

Extended Data Figure 11. Subsurface stone no. 29, Facet A, Coating 1

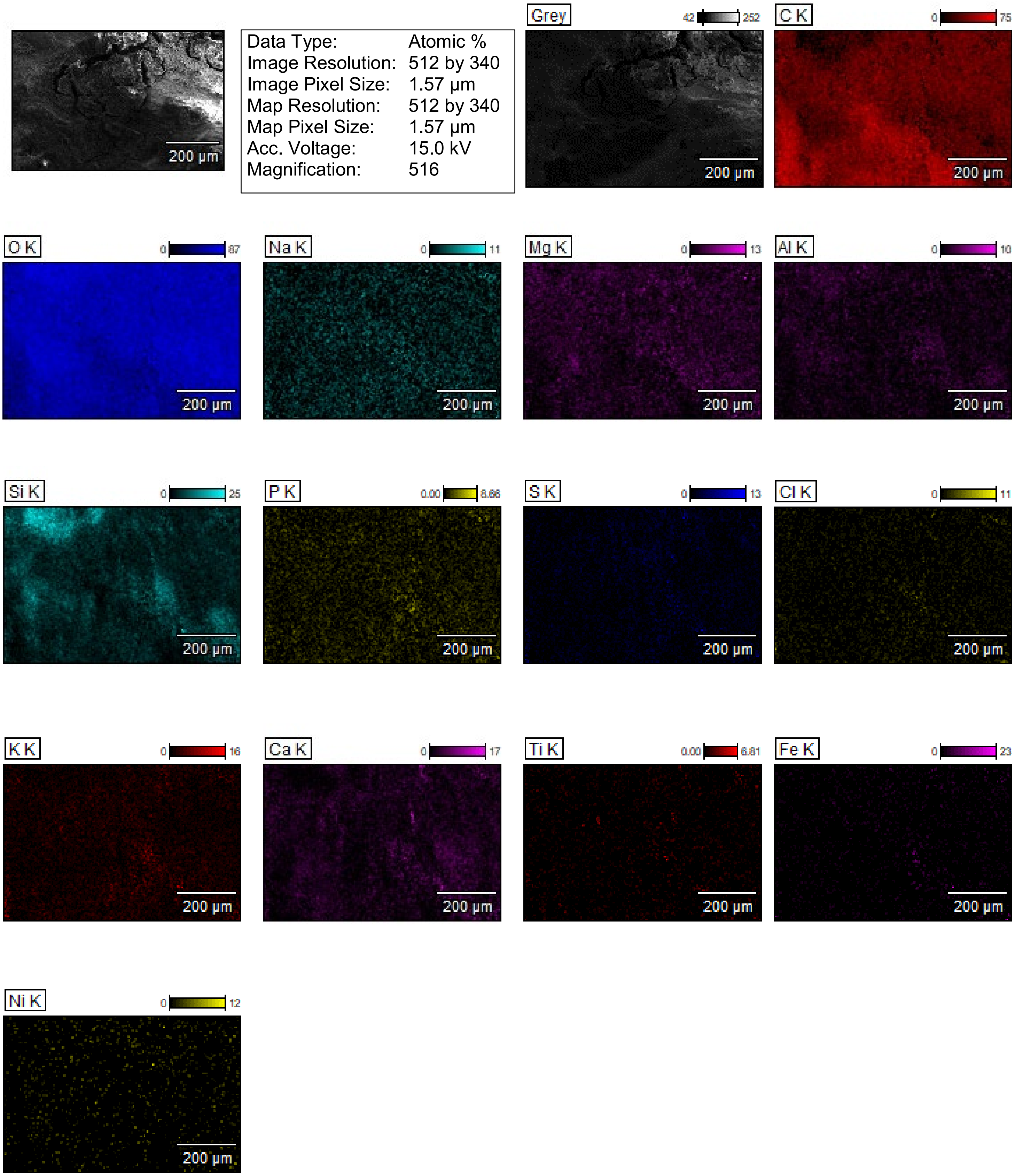

Subsurface 29 A (1)						
Element	Net Counts	Weight %	Weight % err	Atom %	Norm. Wt. %	Chemical Formula
C K	222534	25.51	0.12	34.27	25.51	C
O K	325273	55.13	0.33	55.60	55.13	O
Na K	17658	1.33	0.03	0.93	1.33	Na
Mg K	34650	1.98	0.02	1.32	1.98	Mg
Al K	20827	1.04	0.01	0.62	1.04	Al
Si K	124478	6.04	0.03	3.47	6.04	Si
P K	17832	0.86	0.02	0.45	0.86	P
S K	23283	1.13	0.02	0.57	1.13	S
Cl K	6937	0.42	0.01	0.19	0.42	Cl
K K	33688	2.47	0.03	1.02	2.47	K
Ca K	40571	3.40	0.04	1.37	3.40	Ca
Ti K	543	0.07	0.01	0.02	0.07	Ti
Fe K	1281	0.34	0.05	0.10	0.34	Fe
Ni K	680	0.28	0.03	0.08	0.28	Ni
**% Total**	**-**	**100**	**-**	**100**	**100**	**-**

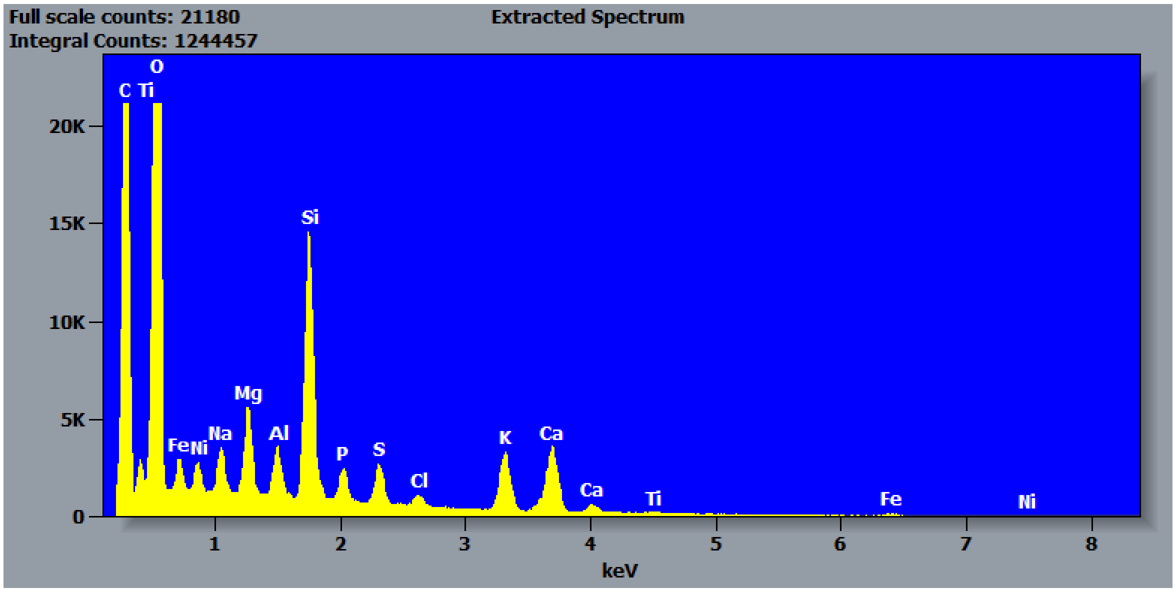

Subsurface 29, Facet A, Coating 2

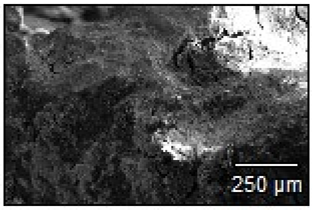

Data Type:	Atomic %
Image Resolution:	512 by 340
Image Pixel Size:	2.41 µm
Map Resolution:	512 by 340
Map Pixel Size:	2.41 µm
Acc. Voltage:	15.0 kV
Magnification:	336

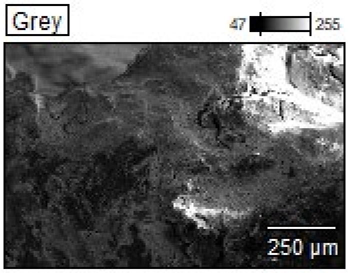

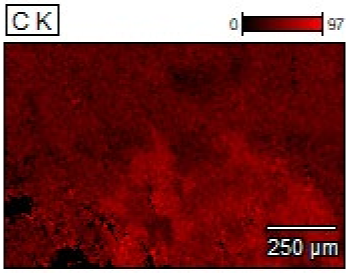

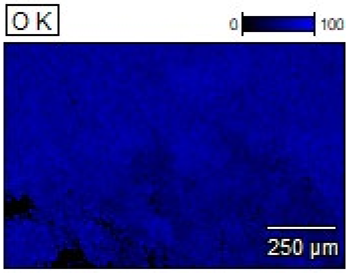

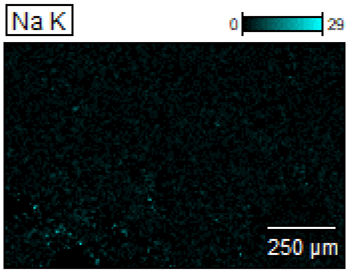

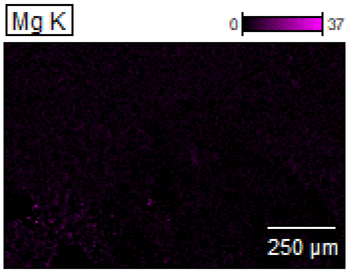

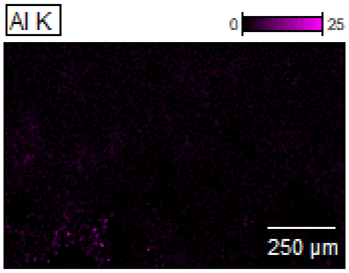

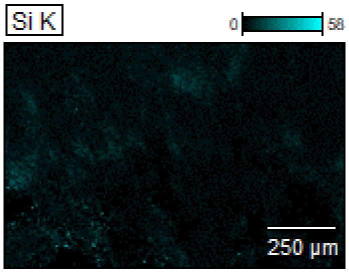

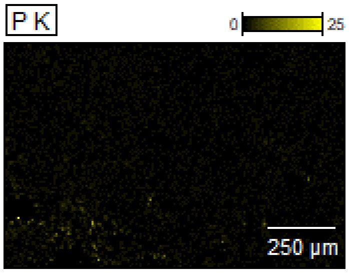

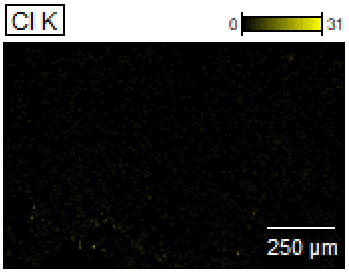

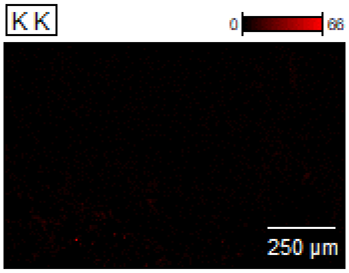

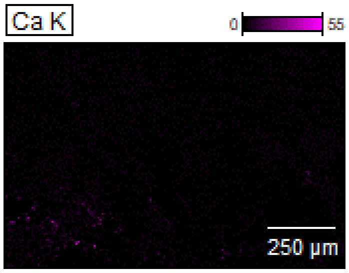

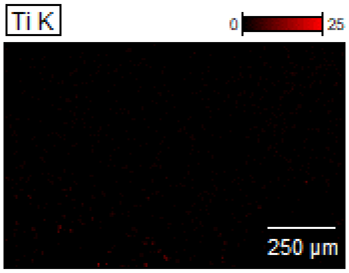

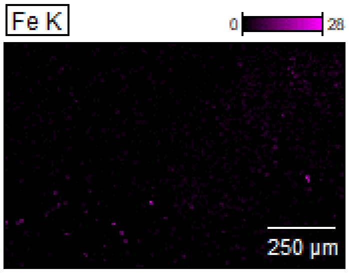

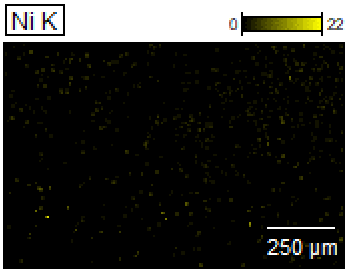

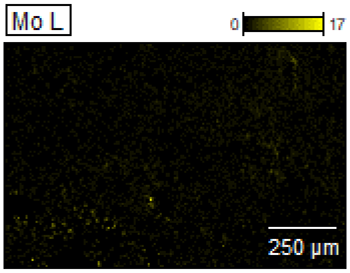

Subsurface 29 A (2)						
**Element**	**Net Counts**	**Weight %**	**Weight % err**	**Atom %**	**Norm. Wt. %**	**Chemical Formula**
C K	183717	27.55	0.14	36.44	27.55	C
O K	238561	56.15	0.37	55.74	56.15	O
Na K	11803	1.30	0.02	0.90	1.30	Na
Mg K	21834	1.81	0.03	1.18	1.81	Mg
Al K	12467	0.89	0.03	0.53	0.89	Al
Si K	67815	4.72	0.04	2.67	4.72	Si
P K	10599	0.73	0.03	0.37	0.73	P
Cl K	5716	0.49	0.02	0.22	0.49	Cl
K K	14071	1.48	0.02	0.60	1.48	K
Ca K	14293	1.70	0.02	0.67	1.70	Ca
Ti K	424	0.08	0.01	0.03	0.08	Ti
Fe K	2073	0.77	0.05	0.22	0.77	Fe
Ni K	774	0.45	0.06	0.12	0.45	Ni
Mo L	16073	1.88	0.05	0.31	1.88	Mo
**% Total**	**-**	**100**	**-**	**100**	**100**	**-**

Subsurface 29, Facet A, Coating 5

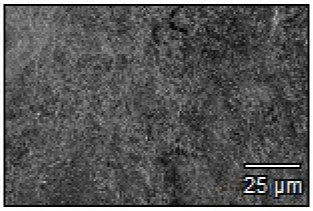

Data Type:	Atomic %
Image Resolution:	512 by 340
Image Pixel Size:	0.27 µm
Map Resolution:	512 by 340
Map Pixel Size:	0.27 µm
Acc. Voltage:	15.0 kV
Magnification:	2996

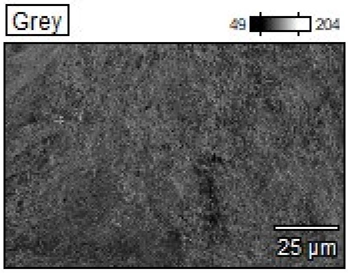

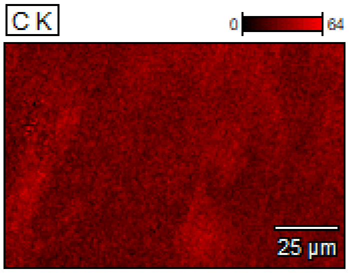

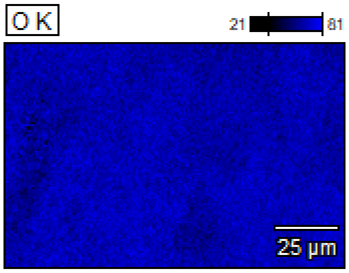

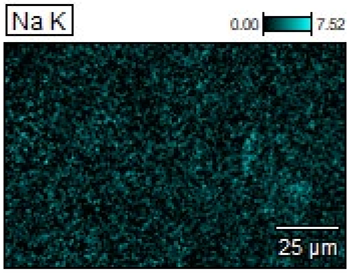

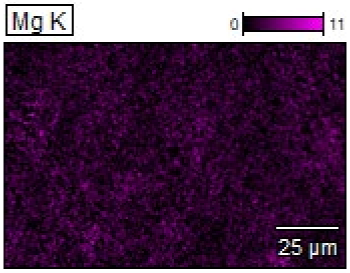

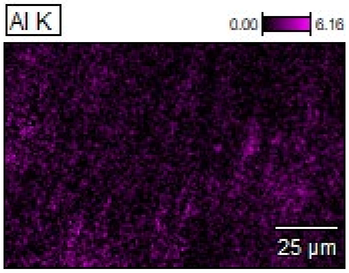

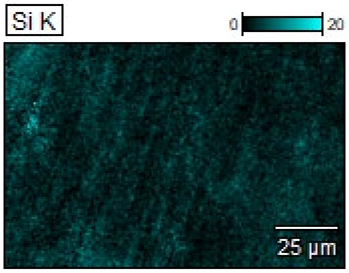

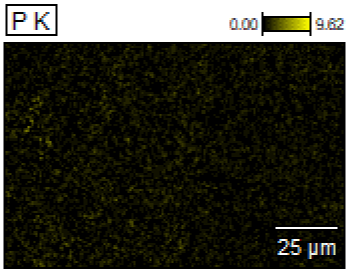

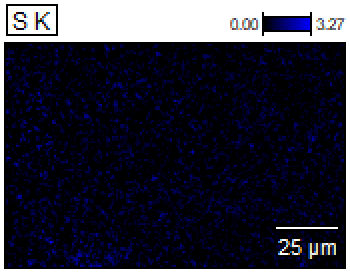

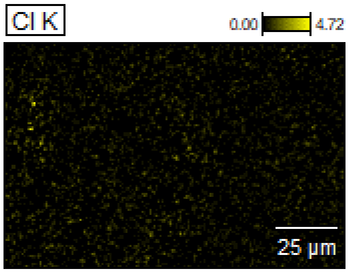

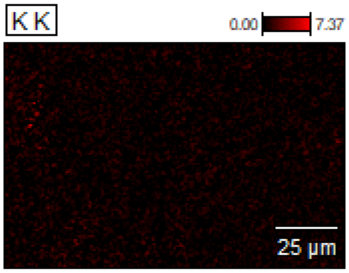

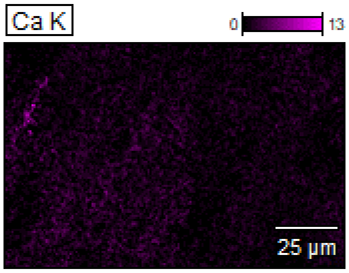

Subsurface 29 A (5)						
**Element**	**Net Counts**	**Weight %**	**Weight % err**	**Atom %**	**Norm. Wt. %**	**Chemical Formula**
C K	131376	21.02	0.10	27.99	21.02	C
O K	318857	63.38	0.31	63.36	63.38	O
Na K	8341	0.92	0.03	0.64	0.92	Na
Mg K	34825	2.90	0.03	1.91	2.90	Mg
Al K	19116	1.40	0.03	0.83	1.40	Al
Si K	82454	5.88	0.04	3.35	5.88	Si
P K	11086	0.79	0.03	0.41	0.79	P
S K	4187	0.30	0.01	0.15	0.30	S
Cl K	940	0.08	0.01	0.04	0.08	Cl
K K	8160	0.87	0.01	0.36	0.87	K
Ca K	20421	2.47	0.04	0.99	2.47	Ca
**% Total**	**-**	**100**	**-**	**100**	**100**	**-**

Subsurface 29, Facet A, Coating 7

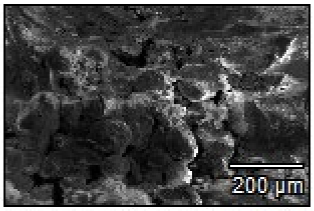

Data Type:	Atomic %
Image Resolution:	512 by 340
Image Pixel Size:	1.62 µm
Map Resolution:	512 by 340
Map Pixel Size:	1.62 µm
Acc. Voltage:	15.0 kV
Magnification:	500

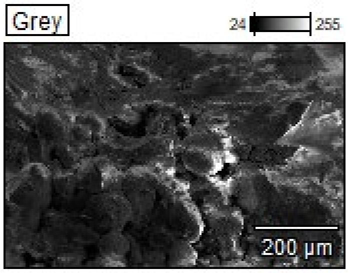

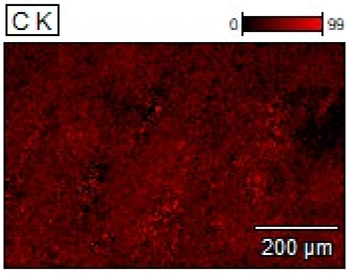

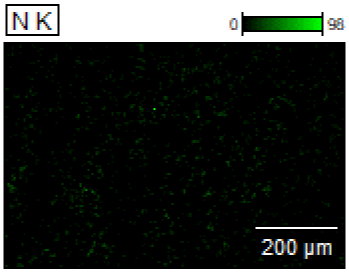

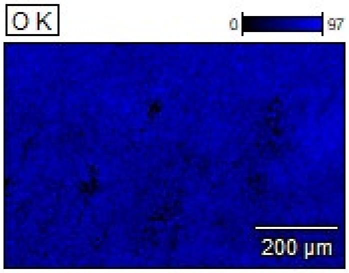

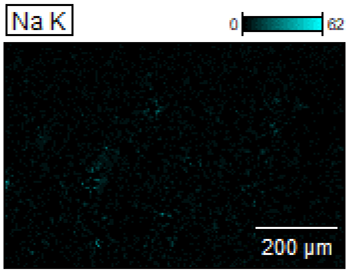

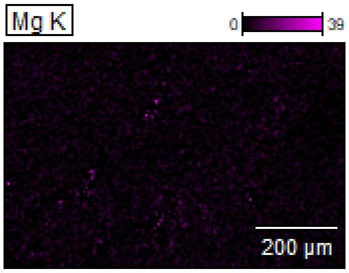

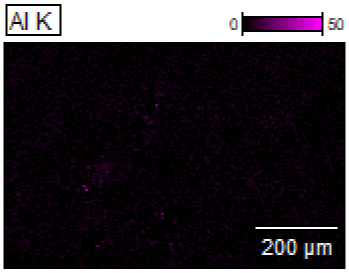

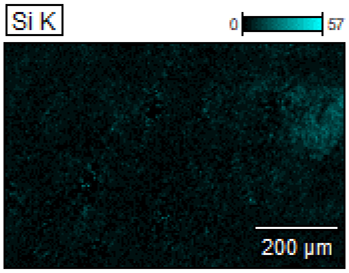

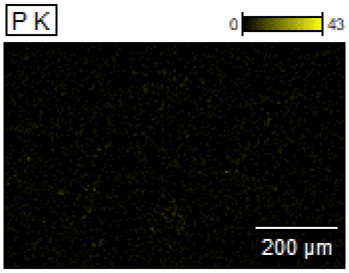

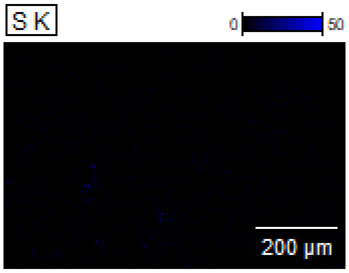

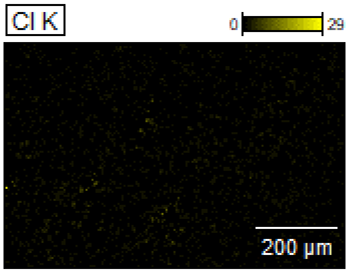

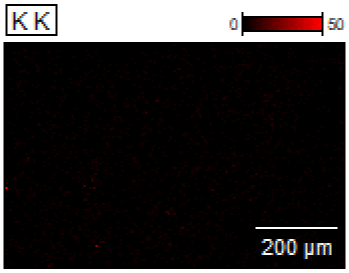

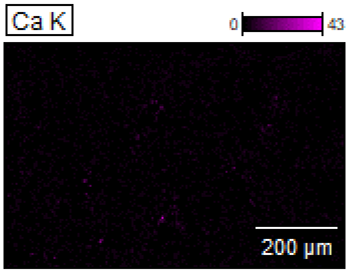

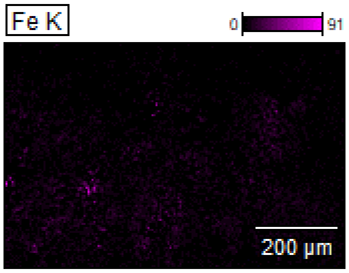

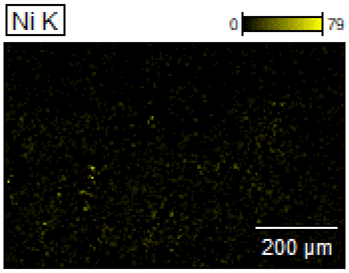

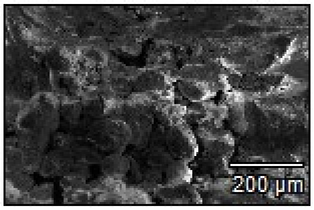

Data Type:	Counts
Image Resolution:	512 by 340
Image Pixel Size:	1.62 µm
Map Resolution:	512 by 340
Map Pixel Size:	1.62 µm
Acc. Voltage:	15.0 kV
Magnification:	500

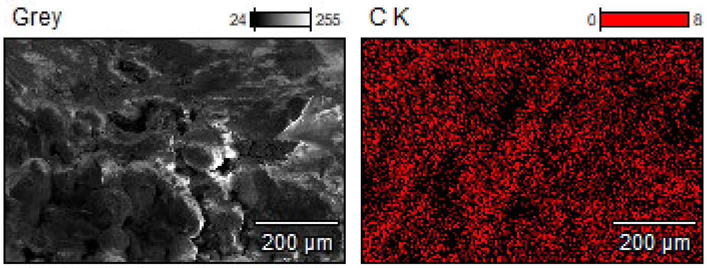

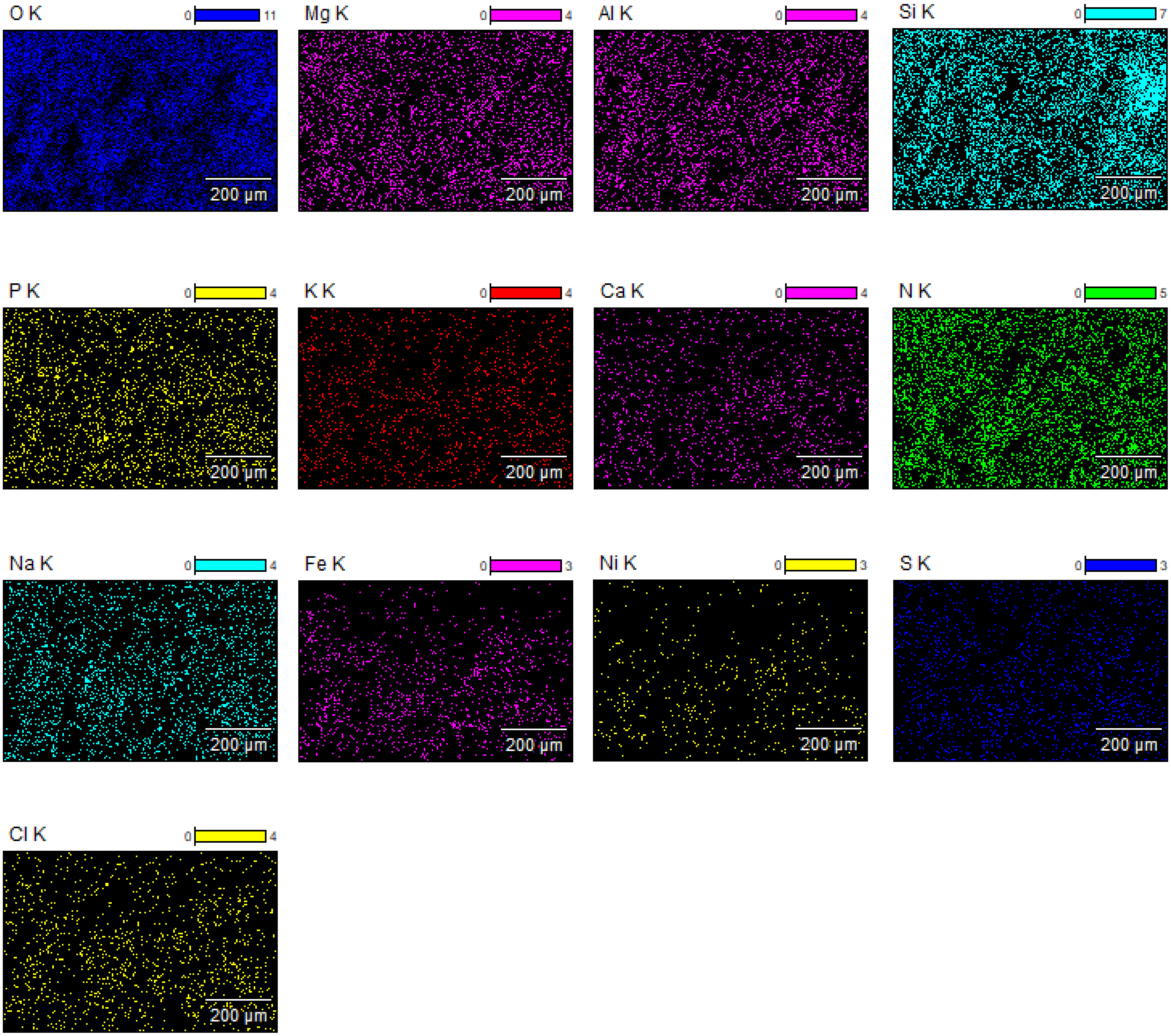

Subsurface 29 A (7)						
**Element**	**Net Counts**	**Weight %**	**Weight % err**	**Atom %**	**Norm. Wt. %**	**Chemical Formula**
C K	93953	23.03	0.13	31.88	23.03	C
N K	1540	1.11	0.32	1.32	1.11	N
O K	179952	54.37	0.36	56.50	54.37	O
Na K	4677	0.81	0.07	0.59	0.81	Na
Mg K	15784	2.05	0.04	1.40	2.05	Mg
Al K	14437	1.62	0.04	1.00	1.62	Al
Si K	57009	6.23	0.05	3.69	6.23	Si
P K	6096	0.66	0.02	0.35	0.66	P
S K	3571	0.39	0.03	0.20	0.39	S
Cl K	824	0.11	0.02	0.05	0.11	Cl
K K	4015	0.65	0.04	0.28	0.65	K
Ca K	4859	0.89	0.03	0.37	0.89	Ca
Fe K	9901	5.62	0.17	1.67	5.62	Fe
Ni K	2738	2.47	0.19	0.70	2.47	Ni
**% Total**	-	**100**	-	**100**	**100**	-

Extended Data Figure 12. Selected images of phytoliths and starch granules from subsurface stones.

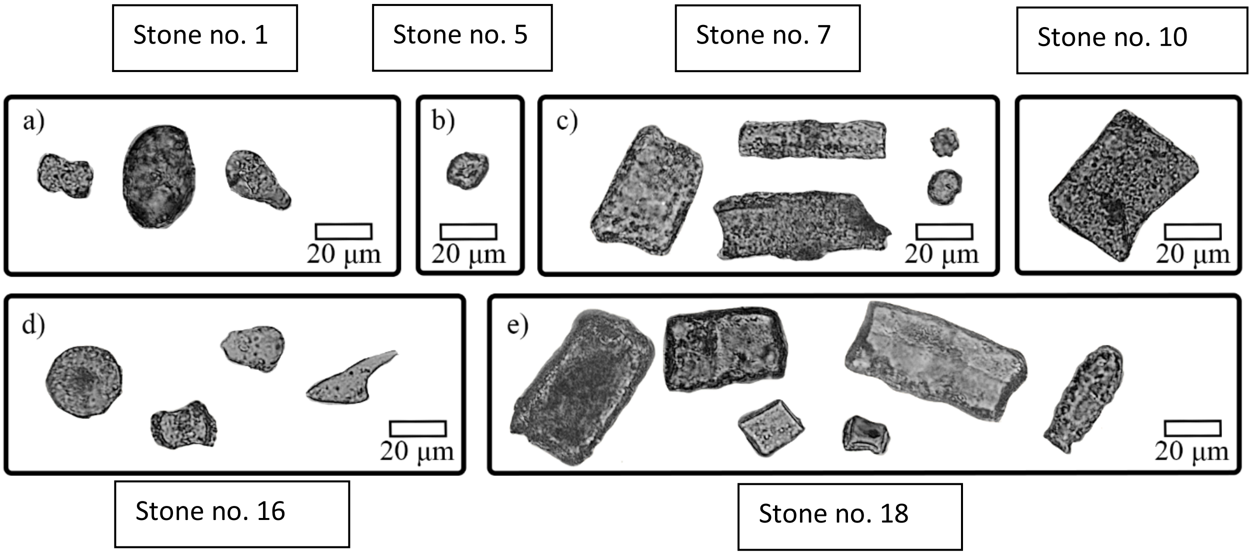

Extended Data Figure 13. Selected microbotanical materials from experimental stones.

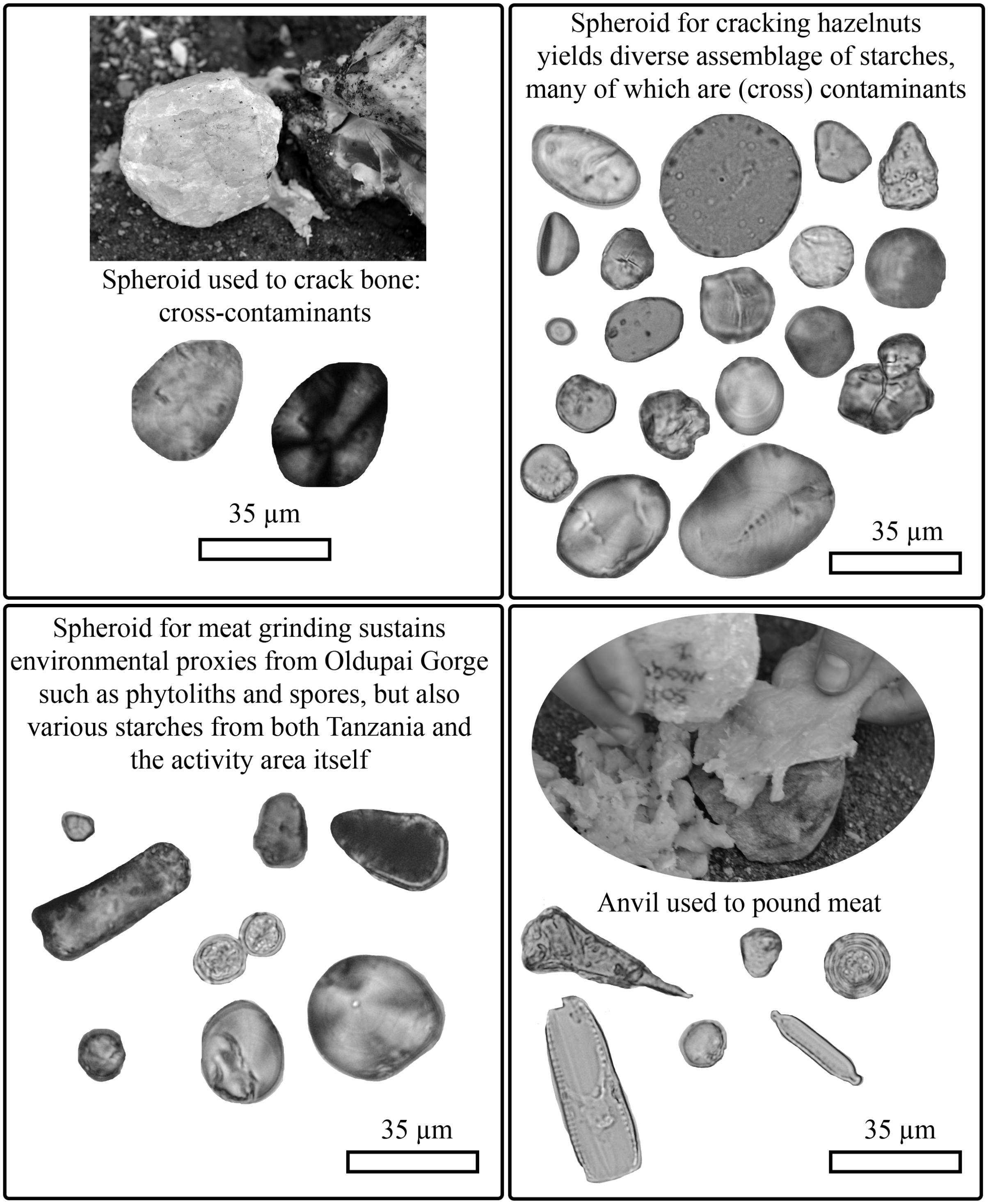

Extended Data Figure 14. Selected microbotanical materials from experimental stones.

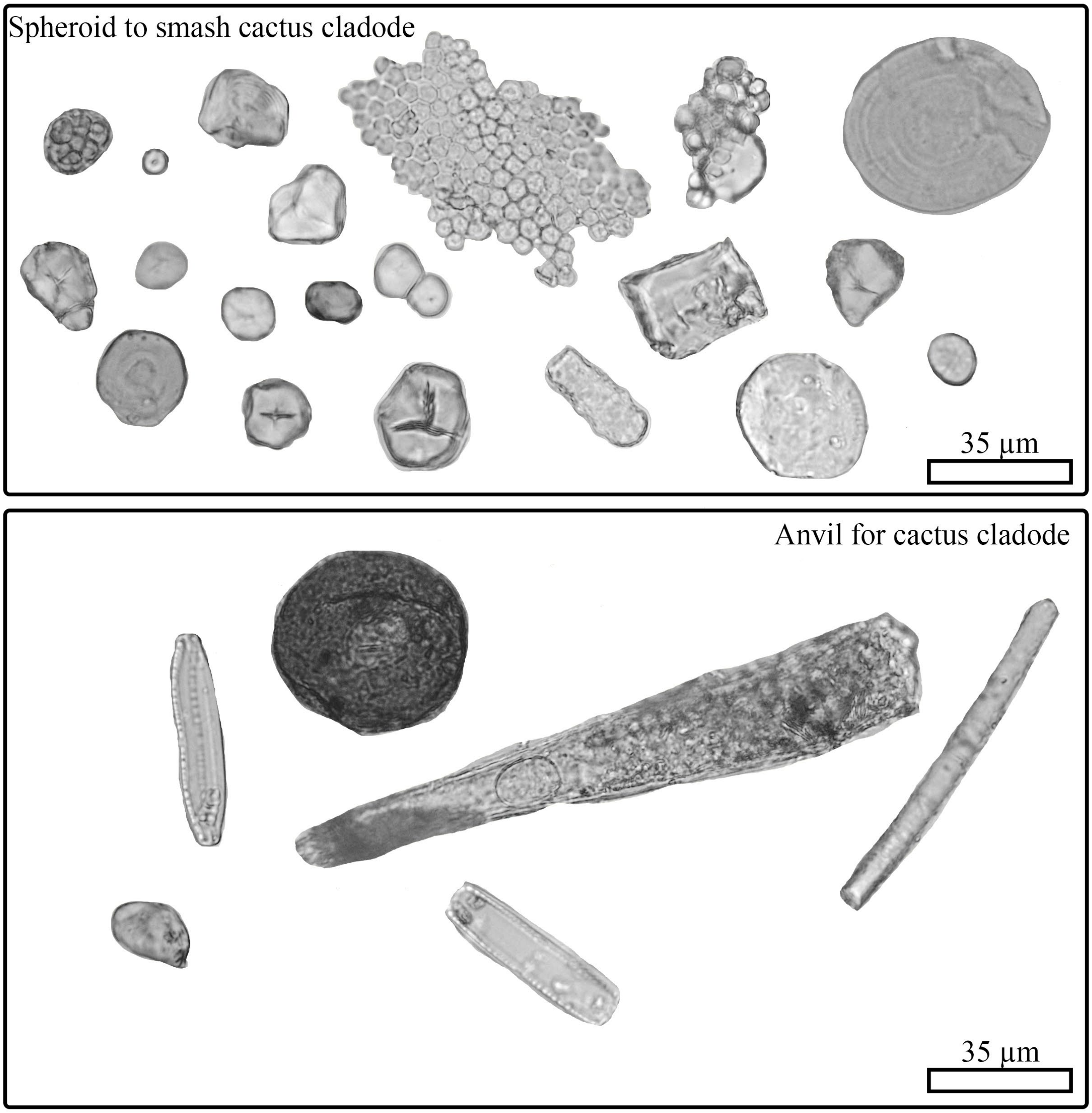

Supplementary Figure 1. Stone assemblages studied in this paper.

Subsurface samples

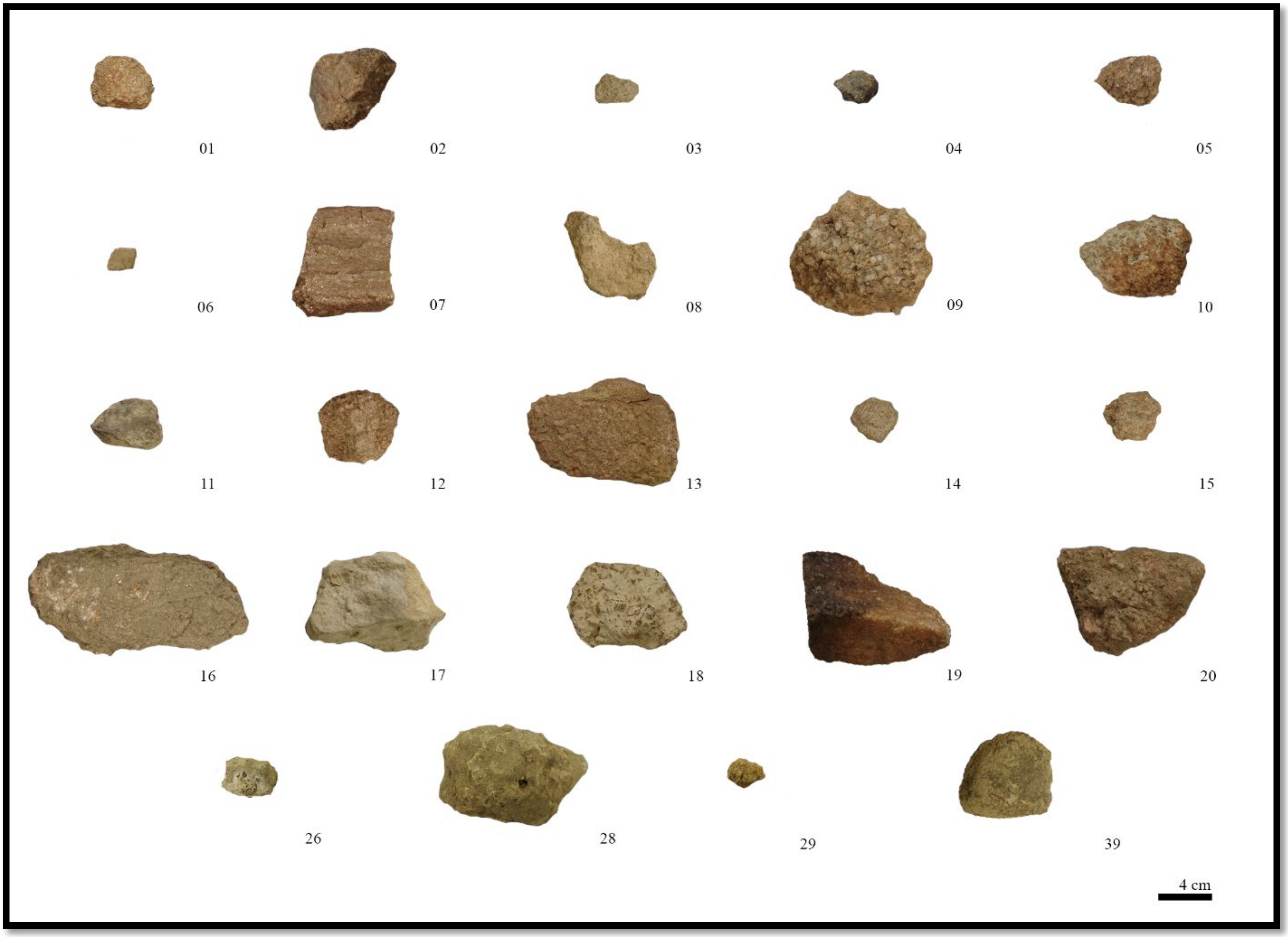

Surface samples

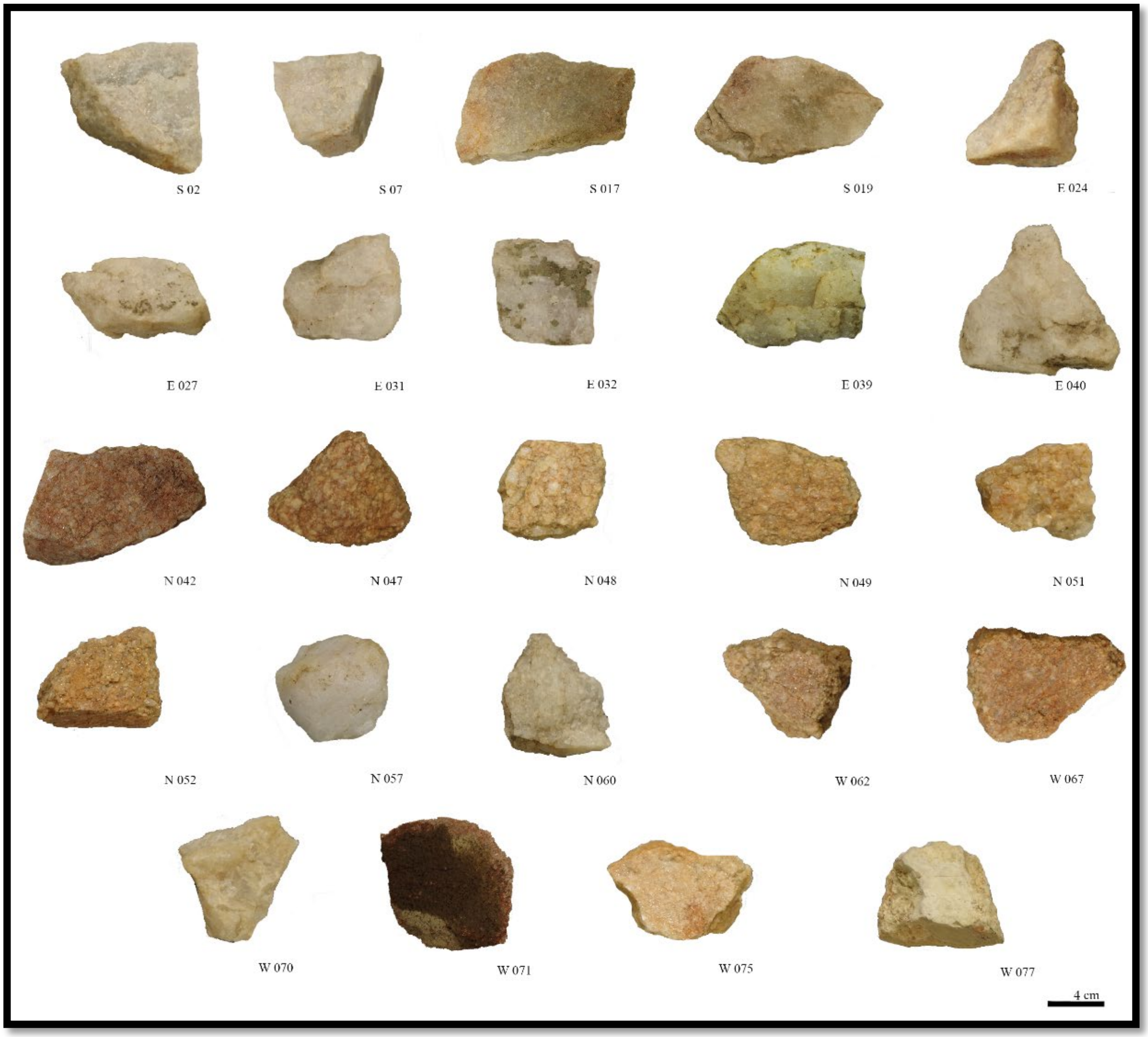

Supplementary Figure 2. Selected images showing quartzite texture (top), microfissure (center), and coating (bottom).

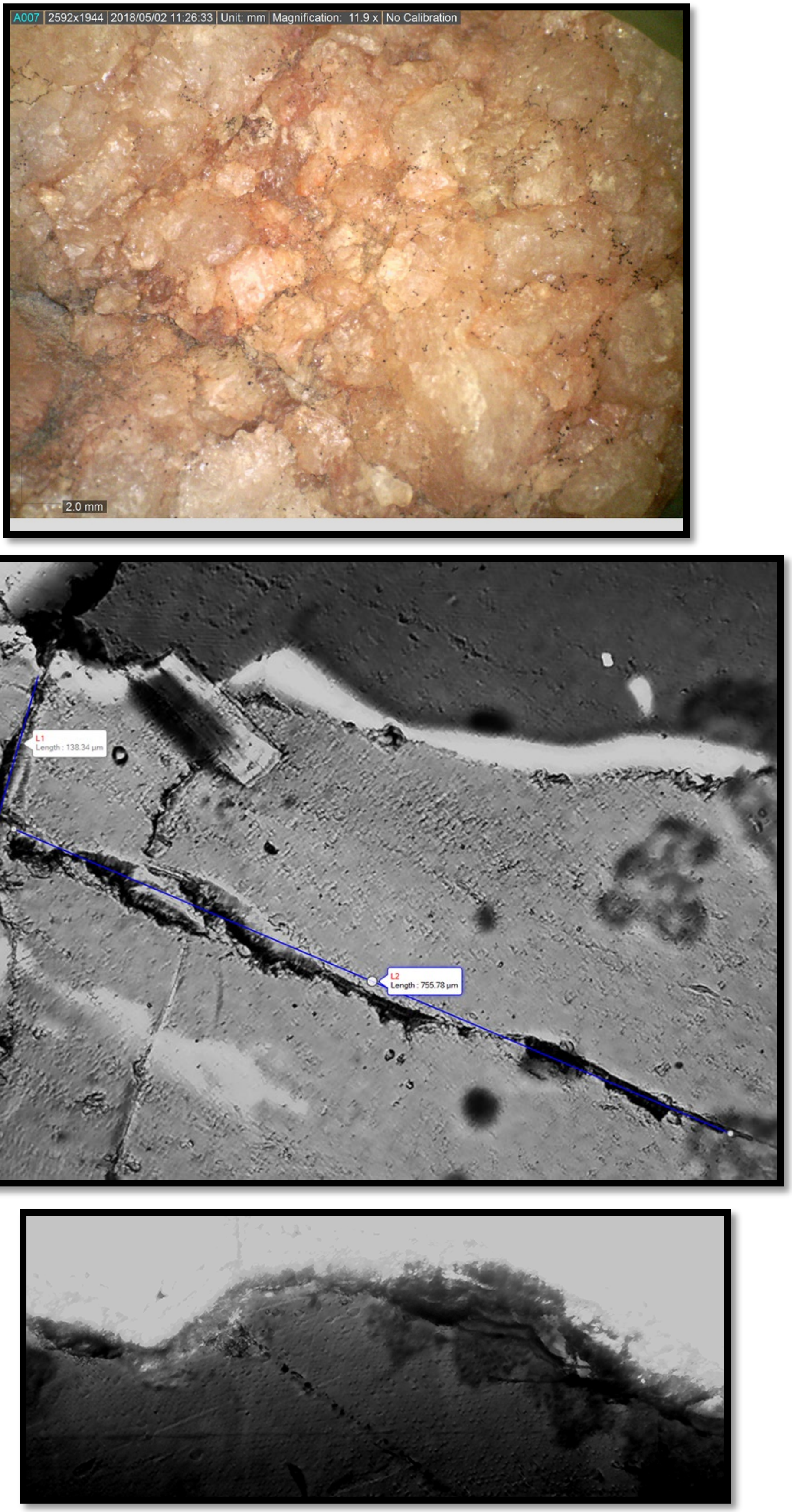

Supplementary Figure 3. Experimental lithics.

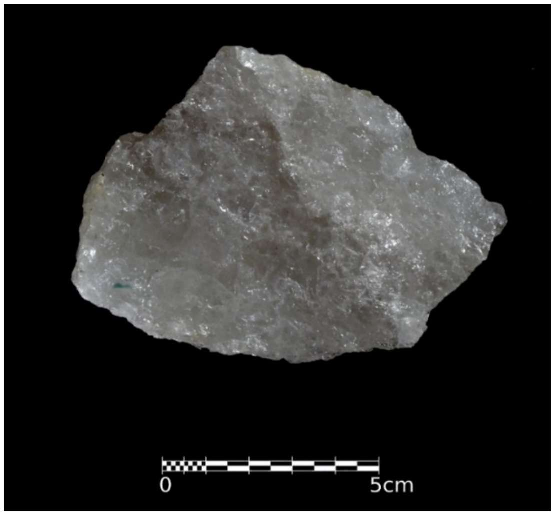

Naibor Soit North 1-2

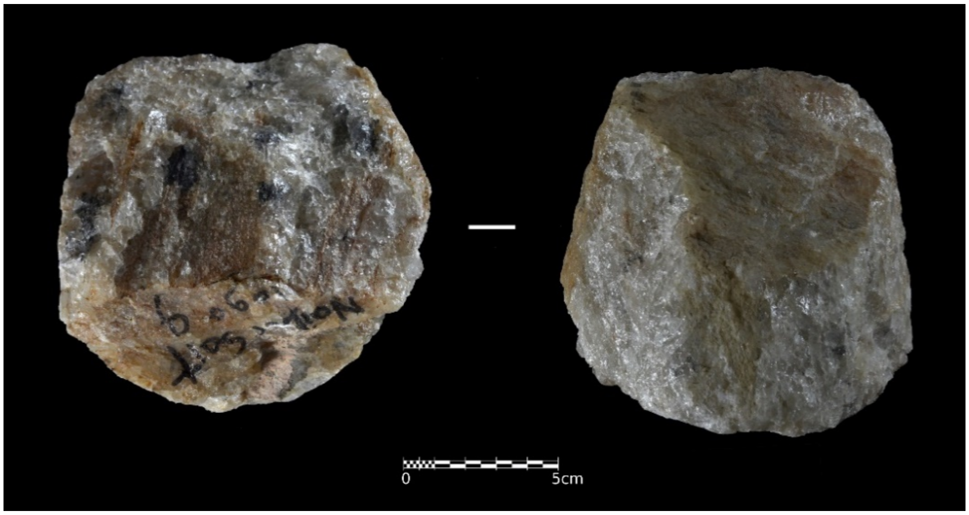

Naibor Soit North 9-1

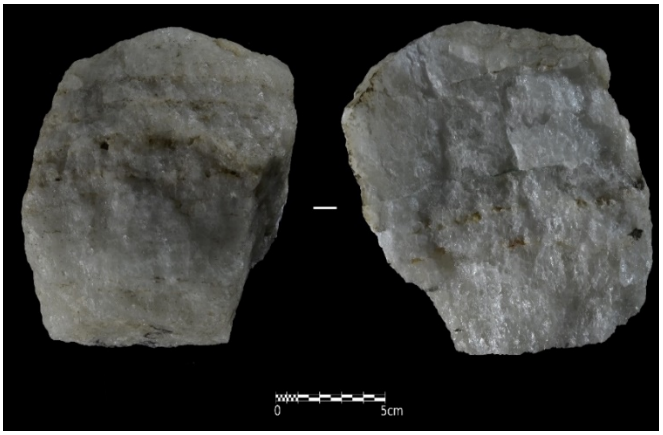

Naibor Soit North 2-1

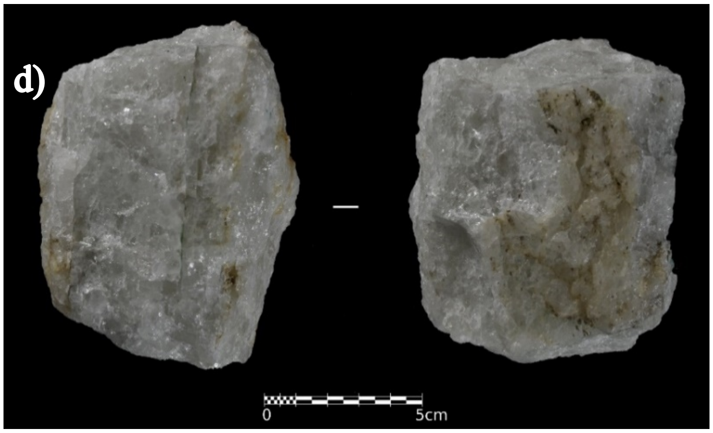

Naibor Soit North 2-2

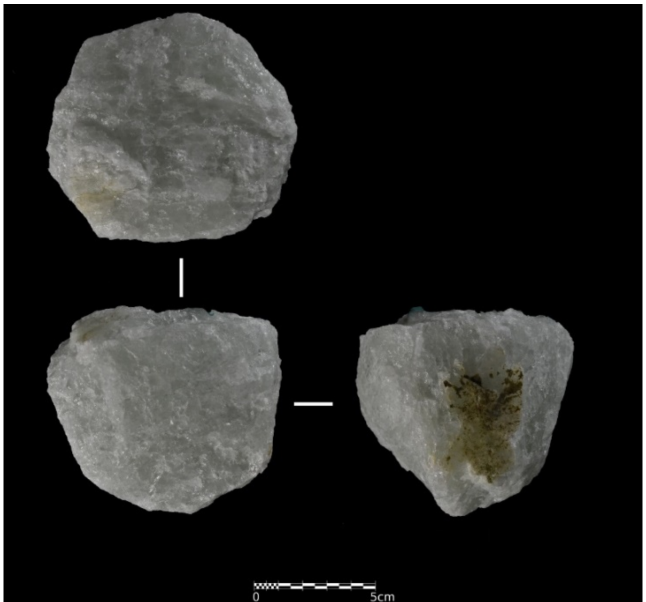

Naibor Soit North 12-1

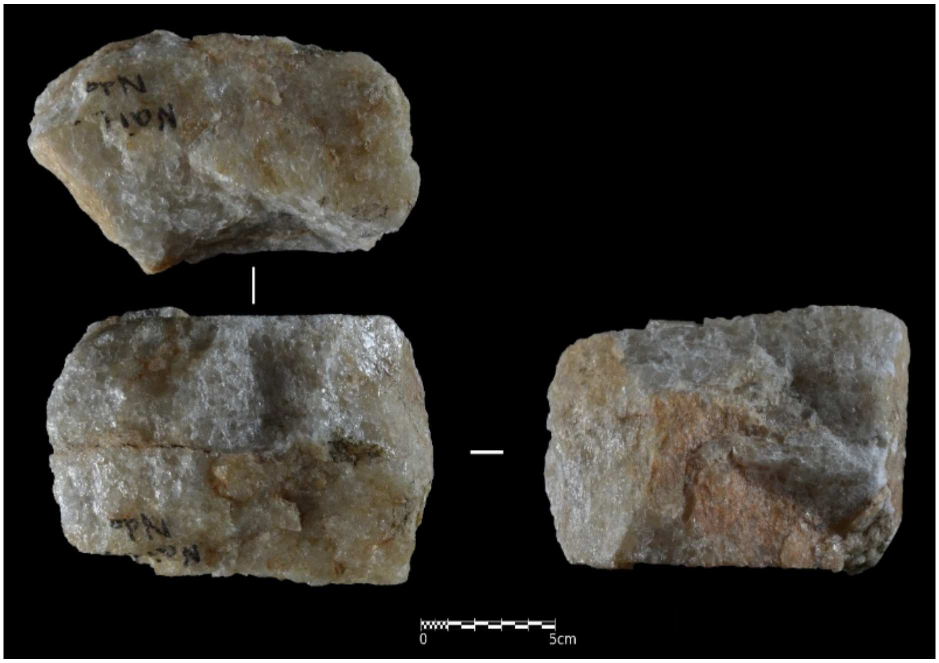

Naibor Soit North 13-1

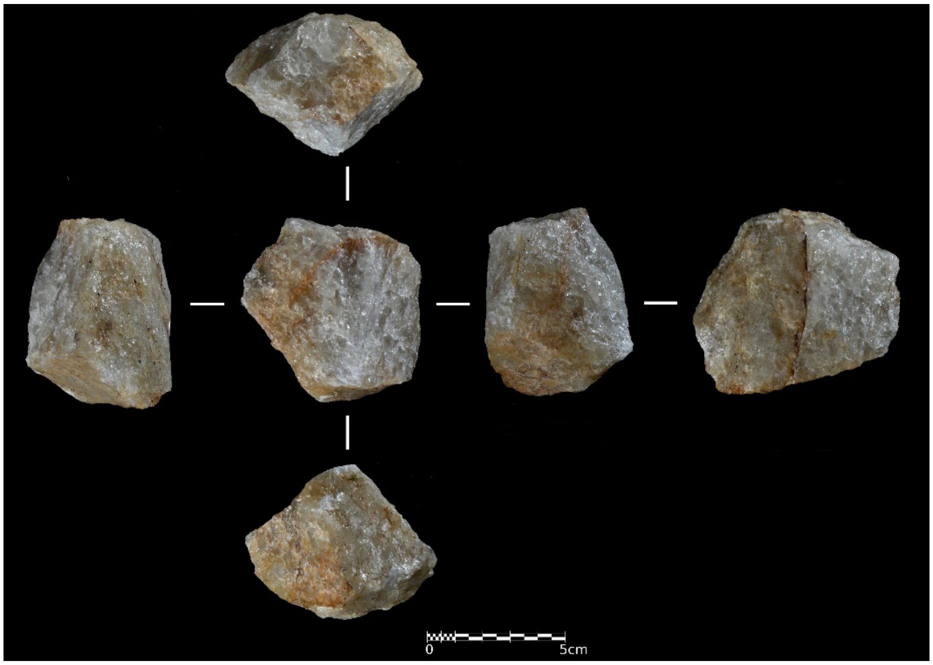

Naibor Soit North 13-2

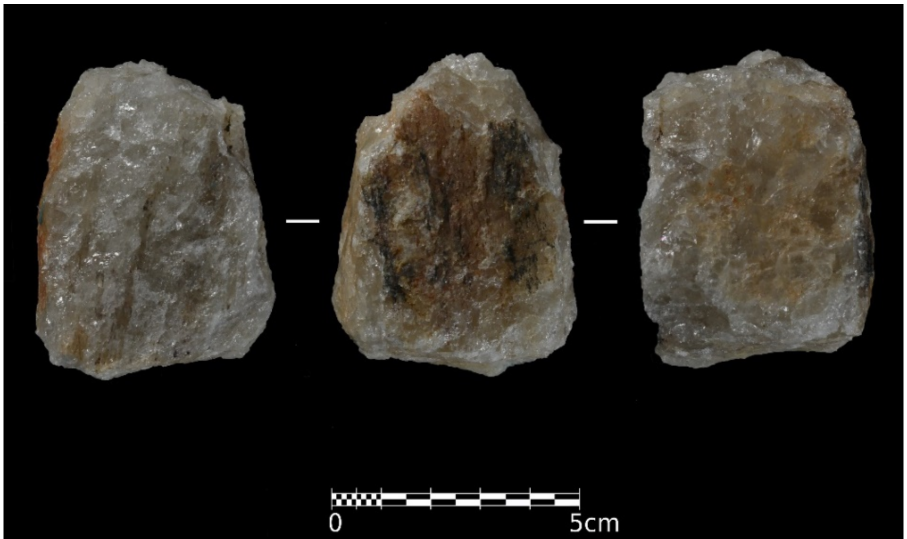

Naibor Soit North 13-3

Supplementary Figure 4. Grid utilized for georeferencing.

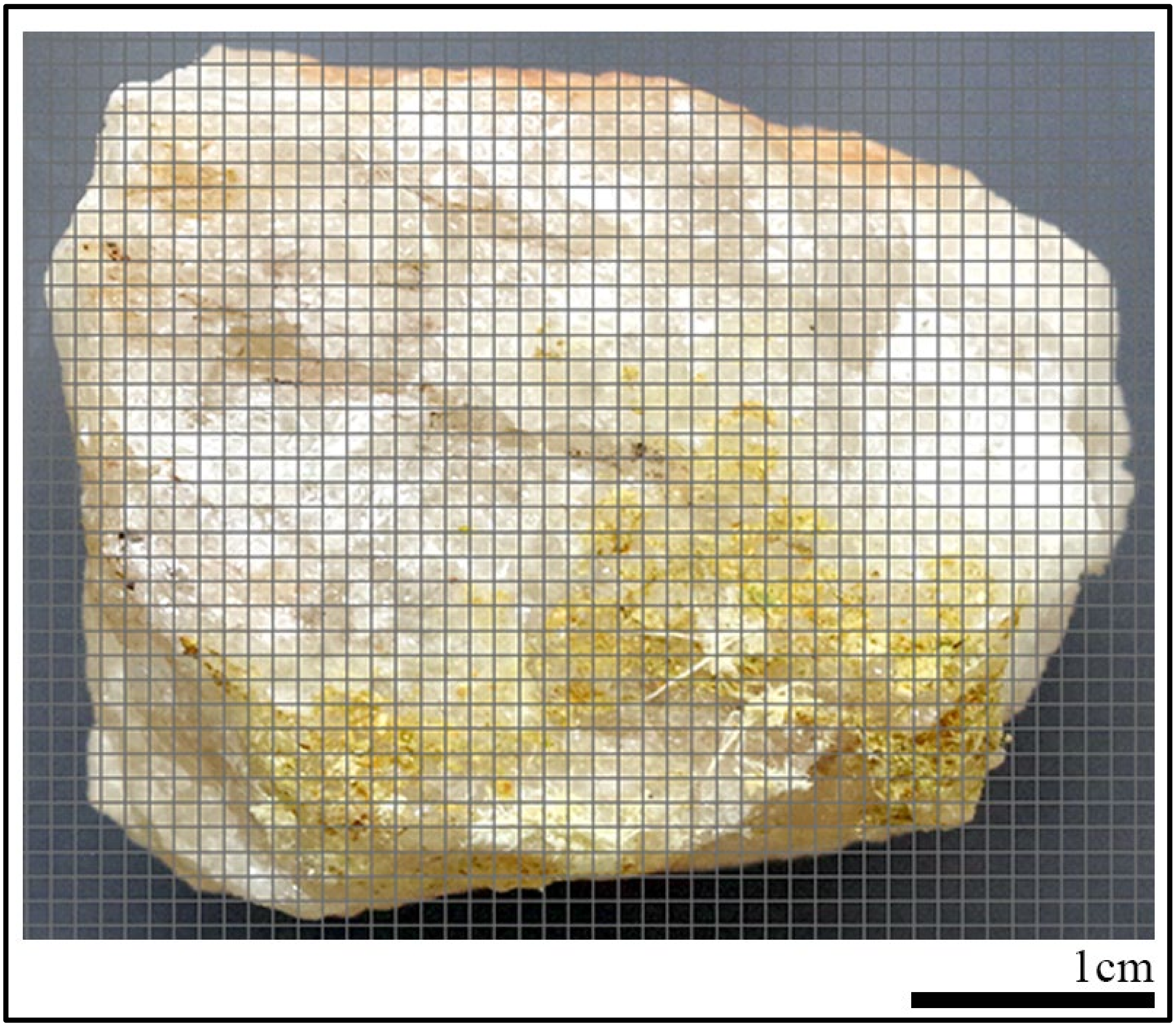

Supplementary Figure 5. Materials utilized during pounding and scraping experiments. Note: Green coloration, noticeable along internal planes of white Naibor Soit quartzite, derives from fuchsite, not necessarily phytogenic material.

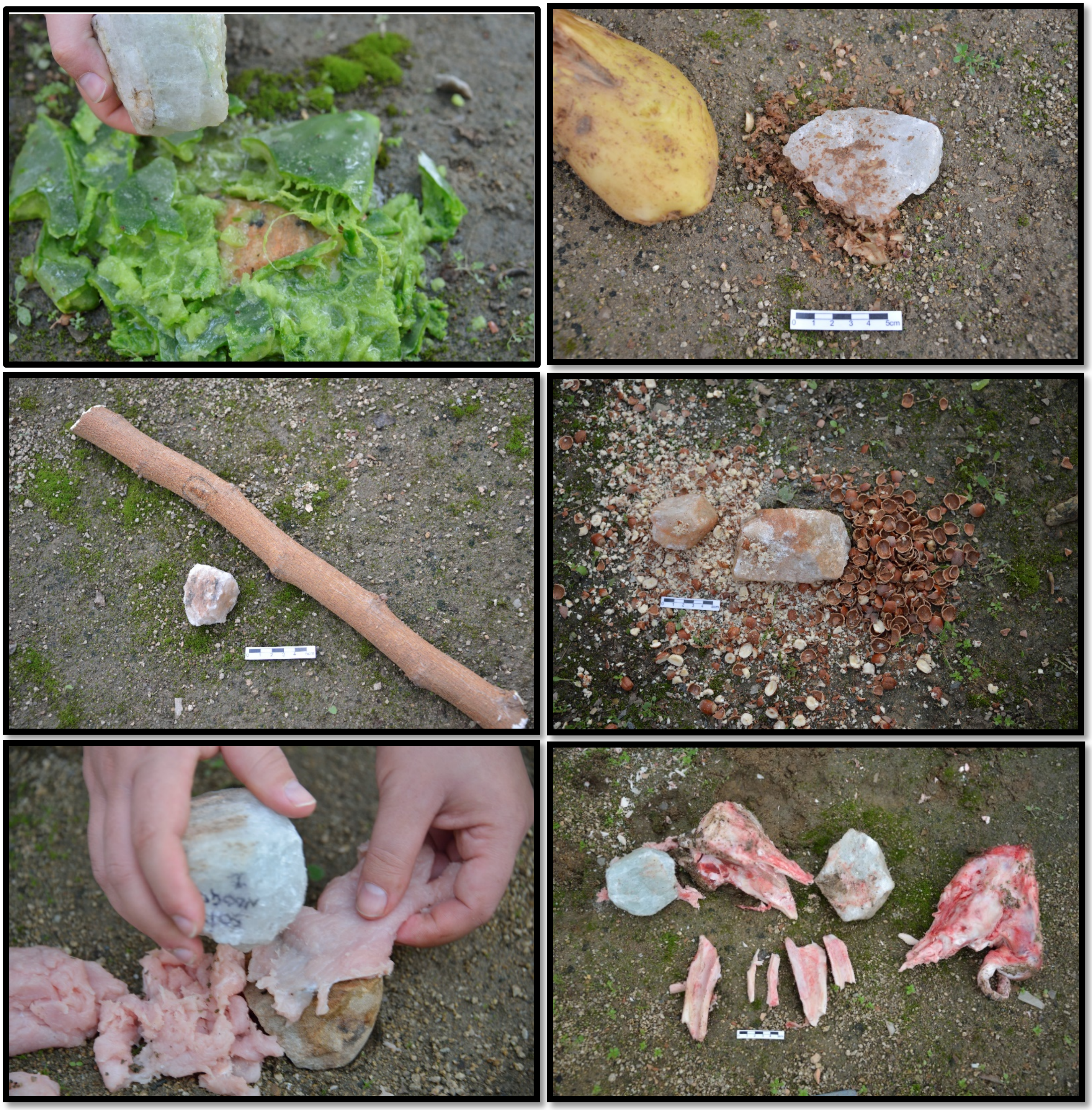

Supplementary Figure 6. Residue distribution after cleaning cycle no. 4 on 'lead' facets (see Supplementary Table 7 for stone metrics), as established by quantitative analysis.

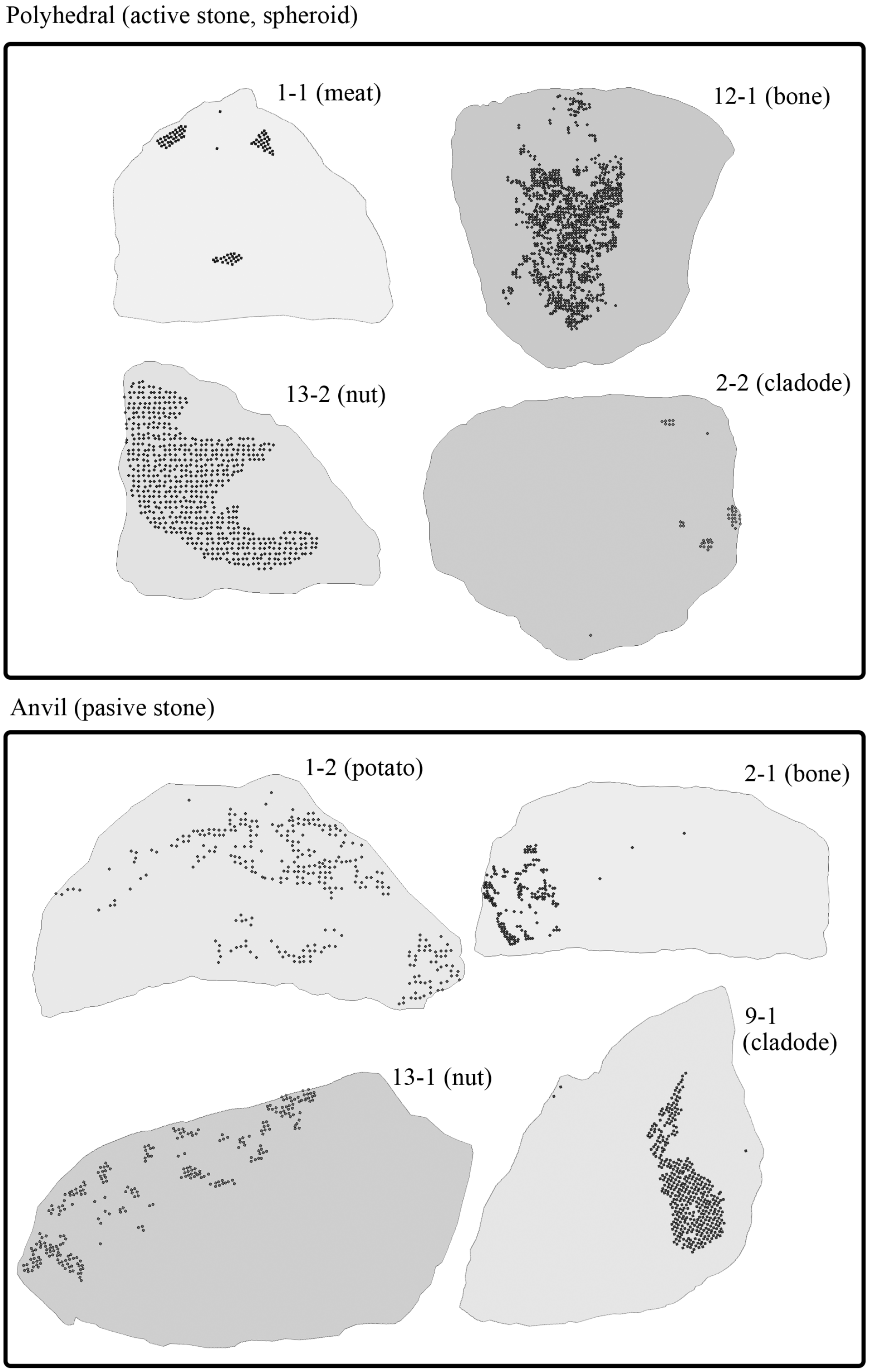

Supplementary Figure 7. Residue distribution after cleaning cycle no. 4 on 'active' facets (see Supplementary Table 7 for stone metrics), as established at the time of use. Elliptical outlines show the active area during pounding. Absence of black dots indicate no residue observed.

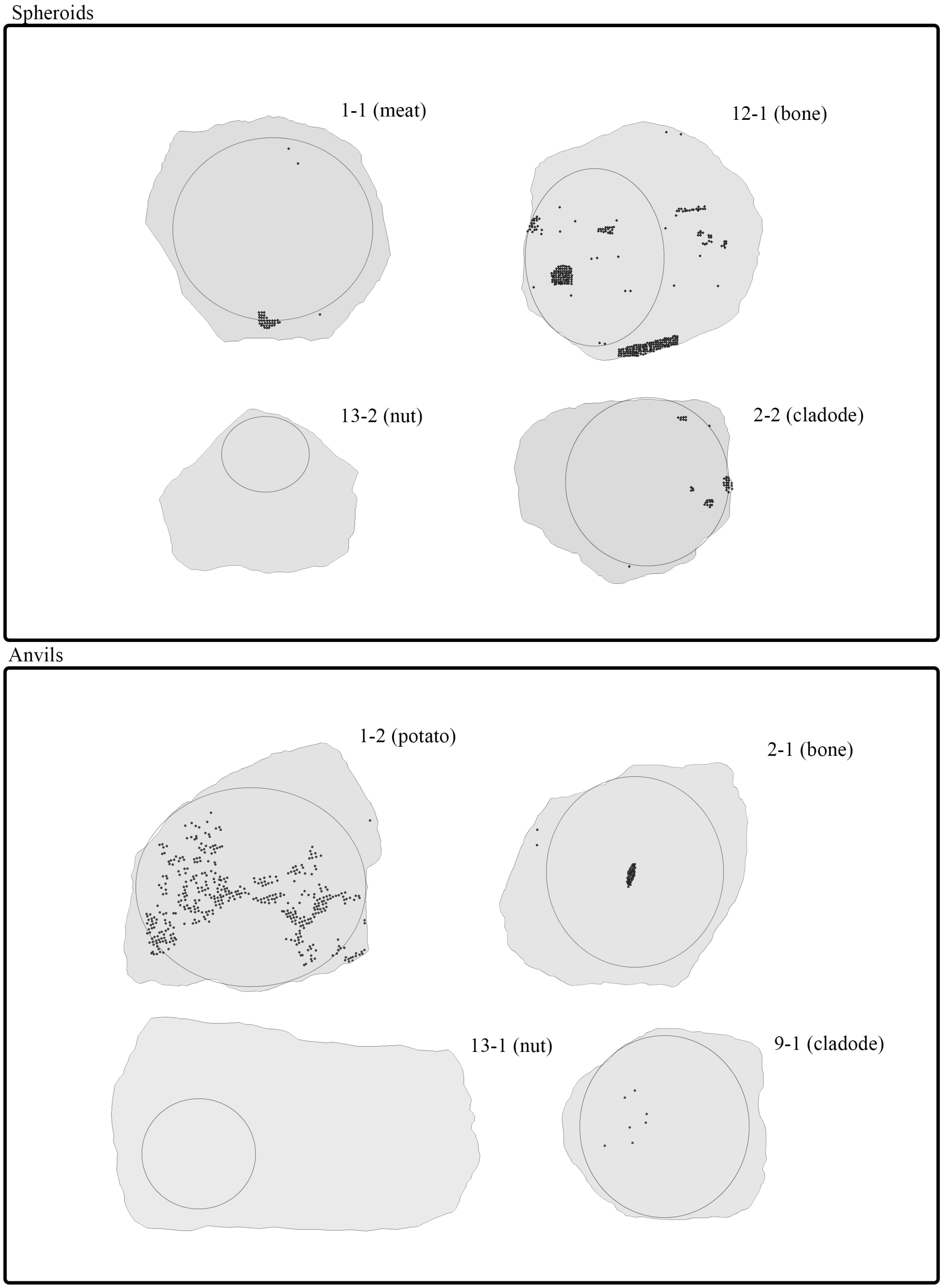

Supplementary Table 1. Stone characterization

ID	Mass (g)	Length (mm)	Width (mm)	Thickness (mm)	Roundness Index	Lithology
Subsurface						
1	73.6	51.1	45.6	29.5	0.6	Very coarse-grain quartzite
2	211.9	75.2	66.2	40.1	0.3	Very coarse-grain quartzite
3	26.3	34.5	24.9	21.5	n/a	Very coarse-grain quartzite
4	18.4	31.4	25.2	17.9	0.6	Coarse-grain quartzite
5	75.5	51.6	44.1	34.7	0.7	Very coarse-grain quartzite
6	14.6	25.1	20.2	15.6	0.7	Sandstone
7	316.2	102.6	94.2	28.8	0.4	Very coarse-grain quartzite
8	63.3	68.7	43.8	23.2	n/a	Calcrete
9	671.4	104.5	88.3	59.2	0.5	Very coarse-grain quartzite
10	284.2	82.3	58.9	47.0	0.4	Very coarse-grain quartzite
11	64.6	52.8	39.8	31.2	0.6	Coarse-grain quartzite
12	130.9	64.3	62.2	41.5	0.5	Very coarse-grain quartzite
13	533.5	120.0	80.8	41.7	0.7	Very coarse-grain quartzite
14	33.0	35.5	34.4	15.9	0.6	Very coarse-grain quartzite
15	56.8	46.1	38.6	27.3	0.8	Very coarse-grain quartzite
16	888.1	179.2	84.7	47.4	0.7	Very coarse-grain quartzite
17	31.4	84.6	66.4	39.4	0.4	Calcrete
18	156.3	93.0	66.7	27.3	0.4	Very coarse-grain quartzite
19	512.0	131.3	83.4	54.5	0.2	Very coarse-grain quartzite
20	535.9	106.2	87.4	56.0	0.4	Very coarse-grain quartzite
**Average**	**234.9**	**77**	**57.8**	**35.0**	**0.5**	
Surface						
**ID**	**Mass (g)**	**Length (mm)**	**Width (mm)**	**Thickness (mm)**	**Roundness Index**	**Lithology**
E 40	397.5	99.7	101.3	35.2	0.3	Coarse-grain quartzite
W 70	340.0	83.8	61.4	45.3	0.3	Coarse-grain quartzite
W 75	370.3	97.3	73.6	36.7	0.3	Very coarse-grain quartzite
N 48	262.9	91.0	76.2	28.6	0.3	Very coarse-grain quartzite
E 27	319.6	96.7	45.9	49.4	0.1	Coarse-grain quartzite
N 60	220.2	85.7	70.2	33.4	0.1	Coarse-grain quartzite
N 49	297.7	103.0	68.8	26.3	0.5	Very coarse-grain quartzite
N 57	225.5	71.9	63.4	44.5	0.6	Fine-grain quartzite
S 7	293.5	84.0	70.2	43.0	0.3	Coarse-grain quartzite
N 52	380.0	99.3	68.7	59.3	0.5	Very coarse-grain quartzite
N 47	314.5	90.1	76.7	36.5	0.8	Very coarse-grain quartzite
E 32	322.6	94.1	79.9	42.6	0.2	Coarse-grain quartzite
E 24	334.2	107.4	88.0	44.8	0.2	Very coarse-grain quartzite
S 19	262.4	118.8	68.9	26.4	0.3	Coarse-grain quartzite
W 62	369.7	89.4	71.8	40.2	0.4	Very coarse-grain quartzite
N 51	345.0	76.0	68.0	38.0	0.5	Very coarse-grain quartzite
W 67	462.0	112.5	81.3	35.2	0.2	Very coarse-grain quartzite
S 2	399.5	112.1	89.1	35.5	0.2	Coarse-grain quartzite
E 31	398.3	82.9	67.3	48.9	0.3	Coarse-grain quartzite
W 77	343.6	82.0	63.0	52.0	0.1	Very coarse-grain quartzite
**Average**	**333.0**	**93.9**	**72.7**	**40.1**	**0.3**	

Supplementary Table 2. Metrics of experimental pounding tools.

Label	Tool type	Length (mm)	Width (mm)	Thickness (mm)	Weight (g)
Surface N 1-1	Polyhedral	82	65	65	531
Surface N 1-2	Anvil	89	67	43	191
Surface N 2-1	Anvil	130	91	52	875
Surface N 2-2	Polyhedral	85	74	64	623
Surface N 9-1	Anvil	105	100	58	693
Surface N 12-1	Polyhedral	89	86	86	825
Surface N 13-1	Anvil	120	77	70	820
Surface N 13-2	Polyhedral	67	61	45	183
Surface N 13-3	Polyhedral	53	40	39	141

Supplementary Table 3. Technical actions and processes during experimental pounding.

Experiment Number	Label	Element	Length (mm)	Width (mm)	Thickness (mm)	Weight (g)	Material	Material, State	Technical Gesture	Motion	Angle	Handedness	Time (min)
Exp.1	Surface N 1-1	Active/Spheroid	82	65	65	531	*Sus domesticus*	Fresh	Grinding	Hitting	90	Right	10
Exp.3	Surface N 13-2	Active/Spheroid	67	61	45	183	*Corylus avellana*	Dry	Cracking, grinding	Hitting	90	Left	20
Exp.3	Surface N 13-1	Passive/Anvil	120	77	70	820	*Corylus avellana*	Dry	Cracking, grinding	Passive	90	-	20
Exp.5	Surface N 12-1	Active/Spheroid	89	86	86	825	*Bos taurus*	Green	Cracking	Hitting	90	Right	5
Exp.5	Surface N 2-1	Passive/Anvil	130	91	52	875	*Bos taurus*	Green	Cracking	Passive	90	-	5
Exp.6	Surface N 13-3	Active/Spheroid	53	40	39	141	*Morus alba*	Green	Scraping	Transverse-bidirectional	90	Left	15
Exp.9	Surface N 1-2	Passive/Anvil	89	67	43	191	*Solanum tuberosum*	Fresh	Peeling	Transverse-unidrectional	-	-	10
Exp.10	Surface N 2-2	Active/Spheroid	85	74	64	623	*Opuntia ficus-indica*	Fresh	Grinding	Hitting	90	Right	20
Exp.10	Surface N 9-1	Passive/Anvil	105	100	58	693	*Opuntia ficus-indica*	Fresh	Grinding	Passive	90	-	20

Supplementary Table 4. Microscopy workflow at IPHES-CERCA.

				Low Vac. SEM		FESEM	
Label		Stereo-microscope	3D Digital microscope	BSED (BSE)	LFD (SE)	T1/ CBS (BS)	ETD (SE)
Surface	E 31	X	X	X	X	X	X
Surface	N 42	X				X	X
Surface	N 47	X					
Surface	S 17	X		X	X	X	
Subsurface	7	X	X				
Subsurface	8	X	X			X	X
Subsurface	29	X					X
Subsurface	39		X				

Supplementary Table 5. Statistical analysis comparing residue distribution and microbotanical remains per cleaning cycle.

Summary of Findings

	Total Diatom	Total Phytolith	Total Starch	Non-Pollen Palynomorph	Total
Surface N 1-1	1	2	6	19	28
Surface N 1-2	136	34	10	105	285
Surface N 12-1	0	0	1	0	1
Surface N 13-1	187	1	22	117	327
Surface N 13-2	2	0	41	45	88
Surface N 2-1	2	1	24	36	63
Surface N 2-2	0	2	25	28	55
Surface N 9-1	89	0	0	0	89
**Total**	**417**	**40**	**129**	**350**	**936**

Normality Test

	Residue Area	Diatom	Phytolith	Starch	Other	Total
N	8	6	4	6	6	8
Shapiro-Wilk W	0.84	0.80	0.66	0.93	0.96	0.95
p(normal)	0.08	0.06	0.00	0.58	0.81	0.68
Anderson-Darling A	0.57	0.58	0.76	0.28	0.21	0.27
p(normal)	0.09	0.07	0.01	0.51	0.75	0.57
p(Monte Carlo)	0.10	0.07	0.00	0.57	0.81	0.63
Lilliefors L	0.28	0.28	0.43	0.22	0.21	0.18
p(normal)	0.06	0.16	0.01	0.50	0.58	0.59
p(Monte Carlo)	0.06	0.17	0.00	0.51	0.58	0.60
Jarque-Bera JB	1.73	0.85	0.96	0.48	0.46	0.70
p(normal)	0.42	0.65	0.62	0.79	0.80	0.71
p(Monte Carlo)	0.07	0.18	0.00	0.64	0.68	0.45

Stone Area (cm^2) and Microbotany

	Residue Area	Diatom	Phytolith	Starch	Non-Pollen Palynomorph	Total
Surface N 1-1 C4	1.35	1	2	6	19	28
Surface N 1-2 C4	14.54	117	26	9	12	164
Surface N 2-1 C4	4.50	2	1	24	36	63
Surface N 2-2 C4	0.84	0	2	25	28	55
Surface N 9-1 C4	6.99	89	0	0	0	89
Surface N 12-1 C4	22.63	0	0	1	0	1
Surface N 13-1 C4	3.34	31	0	41	20	92
Surface N 13-2 C4	6.51	2	0	0	45	47

Kendall's Tau Correlation: Area (cm^2) and Microbotany

	Residue Area	Diatom	Phytolith	Starch	Other	Total
Residue Area	-	0.05	0.71	0.19	0.85	0.80
Diatom	0.69	-	0.60	0.50	0.44	0.02
Phytolith	0.18	0.33	-	0.71	-0.91	0.71
Starch	-0.47	0.33	-0.18	-	0.20	0.19
Other	0.07	-0.32	0.06	0.62	-	0.35
Total	0.07	0.83	0.18	0.47	-0.33	-

Relationship: Microbotany Content versus Cycle

	Diatom	Phytolith	Starch	Other
Surface N 1-2 C1	3	4	1	77
Surface N 1-2 C2	0	0	0	0
Surface N 1-2 C3	16	4	0	16
Surface N 1-2 C4	117	26	9	12
Surface N 13-1 C1	36	0	0	7
Surface N 13-1 C2	19	1	0	65
Surface N 13-1 C3	101	0	9	25
Surface N 13-1 C4	31	0	13	20

Normality Tests for Surface N 1-2 Relationship: Microbotany Content versus Cycle

	Cycle 1	Cycle 2	Cycle 3	Cycle 4
N	4.00	-	4.00	4.00
Shapiro-Wilk W	0.66	-	0.83	0.75
p(normal)	0.00	-	0.16	0.03
Anderson-Darling A	0.76	-	0.41	0.58
p(normal)	0.01	-	0.16	0.04
p(Monte Carlo)	0.00	-	0.19	0.04
Lilliefors L	0.43	-	0.30	0.37
p(normal)	0.01	-	0.23	0.06
p(Monte Carlo)	0.00	-	0.20	0.06
Jarque-Bera JB	0.96	-	0.58	0.87
p(normal)	0.62	-	0.75	0.65
p(Monte Carlo)	0.00	-	0.28	0.04

Dunn´s Post-Hoc: Analyzing Differences on Surface N 1-2 Microbotany Distributions

	Cycle 1	Cycle 2	Cycle 3	Cycle 4
Surface N 1-2 C1	-	0.06	1.00	0.27
Surface N 1-2 C2	0.06	-	0.06	0.00
Surface N 1-2 C3	1.00	0.06	-	0.27
Surface N 1-2 C4	0.27	0.00	0.27	-

Normality Tests for Surface N 13-1

	Cycle 1	Cycle 2	Cycle 3	Cycle 4
N	4.00	4.00	4.00	4.00
Shapiro-Wilk W	0.76	0.82	0.82	1.00
p(normal)	0.05	0.14	0.14	0.99
Anderson-Darling A	0.55	0.44	0.45	0.16
p(normal)	0.06	0.13	0.12	0.85
p(Monte Carlo)	0.06	0.14	0.13	0.98
Lilliefors L	0.34	0.28	0.33	0.16
p(normal)	0.12	0.34	0.15	4.03
p(Monte Carlo)	0.12	0.34	0.14	0.97
Jarque-Bera JB	0.81	0.68	0.76	0.24
p(normal)	0.67	0.71	0.68	0.89
p(Monte Carlo)	0.07	0.15	0.10	0.91

Kruskal-Wallis Test for Surface N 13-1

H (Chi^2)	0.8603
Hc (tie corrected)	0.8864
p (same)	0.8287

Supplementary Table 6. Surface stone metrics per facet, along with residue area and percentage.

	Side cm^2	Residue cm^2	Residue %
Surface E 31 Side A	55.2	0	0
Surface E 31 Side B	50.0	12.8	25.7
Surface E 31 Side C	37.0	0	0
Surface E 31 Side D	46.8	0.4	0.9
Surface E 31 Side E	50.0	0	0
Surface E 31 Side F	32.8	0	0
Surface E 39 Side A	90.7	0.01	0
Surface E 39 Side B	101.2	0.01	0
Surface E 39 Side C	17.5	0	0
Surface N 42 Side A	31.0	0.01	0
Surface N 42 Side B	63.8	0	0
Surface N 42 Side C	20.0	0.03	0.2
Surface N 42 Side D	49.1	0	0
Surface N 42 Side E	39.1	0.02	0.1
Surface N 47 Side A	46.7	0	0
Surface N 47 Side B	43.5	0.03	0.1
Surface N 47 Side C	22.6	0.02	0.1
Surface N 47 Side D	22.8	1.6	7.1
Surface N 47 Side E	11.7	0.02	0.2
Surface S 17 Side A	77.8	0.06	0.1
Surface S 17 Side B	71.8	1.3	1.8
Surface S 17 Side C	3.6	0.03	0.8
Surface S 17 Side D	13.7	0.01	0.1
Surface S 17 Side E	32.0	0	0
Surface S 17 Side F	42.6	0.04	0.1
Surface W 62 Side A	54.2	0.02	0
Surface W 62 Side B	56.3	0	0
Surface W 62 Side C	16.0	0.02	0.1
Surface W 62 Side D	13.7	0.01	0.1
Surface W 62 Side E	32.0	0	0
Surface W 62 Side F	42.6	0.04	0.1
Surface W 71 Side A	52.8	0	0
Surface W 71 Side B	73.1	0	0
Surface W 71 Side C	26.5	0	0
Surface W 71 Side D	43.4	0	0
Surface W 71 Side E	49.9	0	0
Surface W 77 Side A	42.7	0	0
Surface W 77 Side B	49.1	0.02	0
Surface W 77 Side C	30.7	0	0
Surface W 77 Side D	15.7	0	0
Surface W 77 Side E	19.3	0	0
Surface W 77 Side F	62.1	0.01	0

Supplementary Table 7. Subsurface stone metrics per facet, along with residue area and percentage.

	Side cm^2	Residue cm^2	Residue %
Subsurface 5 Side A	17.6	0	0
Subsurface 5 Side B	18	0	0
Subsurface 5 Side C	17.9	0	0
Subsurface 5 Side D	15	0	0
Subsurface 7 Side A	30	0.01	0
Subsurface 7 Side B	65.5	0.03	0
Subsurface 7 Side C	34.7	0	0
Subsurface 7 Side D	37.1	0.02	0.1
Subsurface 7 Side E	24.1	0	0
Subsurface 7 Side F	26.9	0	0
Subsurface 8 Side A	10.9	0	0
Subsurface 8 Side B	7.2	0	0
Subsurface 8 Side C	11.9	0	0
Subsurface 8 Side D	11.7	0	0
Subsurface 18 Side A	52.5	0	0
Subsurface 18 Side B	53.5	0	0
Subsurface 18 Side C	21.5	0	0
Subsurface 26 Side A	10.8	0	0
Subsurface 26 Side B	5.5	0	0
Subsurface 26 Side C	10.3	0	0
Subsurface 26 Side D	4.6	0	0
Subsurface 28 Side A	67.9	0	0
Subsurface 29 Side A	1.9	0.01	0.5
Subsurface 29 Side B	1.8	0	0
Subsurface 29 Side C	1.7	0	0
Subsurface 29 Side D	2.7	0.02	0.7
Subsurface 39 Side A	31.4	0	0
Subsurface 39 Side B	29.5	0.01	0
Subsurface 39 Side C	25.1	0	0
Subsurface 39 Side D	32.9	0.01	0
Subsurface 39 Side E	27.5	0.01	0
Subsurface 39 Side F	31.4	0	0

Supplementary Table 8. Phytolith tally per sample: surface and subsurface stones.

Sample		Bilobate	Blocky	Bulliform	Cylindroid	Epidermis, Leaf, Grass	Globular Granulate	Globular Psilate	Globular Ridged	Globular Ridged with Centric Cavity	Hair	Hemisphere	Rondel	Saddle	Saddle, Long	Sclereid	Scutiform	Shield	Tabular	Tabular Oblong	Tower	Vessel	Total
Subsurface	1	0	2	1	1	0	0	0	0	1	2	0	0	0	0	0	0	0	4	1	1	0	**13**
Subsurface	2	4	1	0	4	0	1	0	0	2	1	0	1	1	0	0	0	0	2	2	0	0	**19**
Subsurface	3	0	0	0	0	0	0	0	0	1	0	0	0	0	0	0	0	0	0	0	0	0	**1**
Subsurface	4	0	0	0	1	0	0	0	0	0	0	0	0	0	0	0	0	0	0	0	0	0	**1**
Subsurface	5	0	0	1	0	0	0	0	0	2	2	2	0	0	0	0	0	0	1	0	0	0	**8**
Subsurface	6	1	0	0	1	0	0	0	0	0	0	0	0	0	0	0	0	0	2	0	0	0	**4**
Subsurface	7	7	6	0	3	0	0	0	0	1	1	4	2	4	0	0	1	0	4	0	5	0	**38**
Subsurface	8	0	0	0	1	0	0	0	0	0	0	0	2	0	0	0	0	0	0	0	0	0	**3**
Subsurface	9	1	1	1	5	0	0	0	0	1	0	0	0	1	0	0	2	0	5	1	0	1	**19**
Subsurface	10	4	0	0	0	0	0	0	0	0	0	0	1	1	0	0	0	0	2	0	1	0	**9**
Subsurface	11	0	3	0	0	0	1	0	0	1	0	0	1	1	0	0	0	0	4	0	0	0	**11**
Subsurface	12	1	3	0	0	0	0	0	0	1	2	1	0	0	0	0	1	0	5	0	0	0	**14**
Subsurface	13	0	1	1	1	0	0	0	0	3	1	0	1	2	0	0	0	0	0	0	0	0	**10**
Subsurface	14	0	1	0	0	0	0	0	0	1	1	0	0	0	0	0	0	0	2	0	0	0	**5**
Subsurface	15	0	0	0	0	0	0	0	0	0	0	0	0	0	0	0	0	0	0	0	0	0	**0**
Subsurface	16	1	2	1	1	0	0	0	0	1	1	0	4	3	1	1	0	0	6	0	1	0	**23**
Subsurface	17	1	5	1	2	0	0	0	0	1	2	0	1	0	0	2	1	0	10	2	0	0	**28**
Subsurface	18	5	5	0	8	0	0	0	0	2	4	0	0	0	0	1	9	0	11	8	0	0	**53**
Subsurface	19	7	1	0	1	0	0	0	0	0	1	1	1	0	0	0	1	0	1	1	0	0	**15**
Subsurface	20	3	5	0	2	1	0	0	0	3	0	0	0	1	0	1	2	0	11	3	1	0	**33**
**Subsurface Total**		**35**	**36**	**6**	**31**	**1**	**2**	**0**	**0**	**21**	**18**	**8**	**14**	**14**	**1**	**5**	**17**	**0**	**70**	**18**	**9**	**1**	**307**
Surface	E 24	1	5	1	0	0	0	0	0	0	0	0	0	0	0	0	0	0	7	1	0	0	**15**
Surface	E 27	0	0	0	3	0	0	0	0	1	0	0	0	0	0	1	1	0	3	3	0	0	**12**
Surface	E 31	2	6	1	6	0	2	0	14	0	1	0	0	0	2	1	2	0	10	0	0	0	**47**
Surface	E 32	0	10	0	1	1	0	0	0	4	0	0	0	0	0	0	0	1	10	1	1	0	**29**
Surface	E 40	0	1	0	0	1	0	0	0	1	4	0	0	0	0	0	0	0	3	1	0	0	**11**
Surface	N 47	0	1	0	1	0	0	0	0	2	0	0	1	0	0	0	0	0	1	0	0	0	**6**
Surface	N 48	2	0	0	2	0	0	0	0	0	2	0	0	2	0	0	0	0	4	0	1	1	**14**
Surface	N 49	1	7	0	7	0	1	1	0	1	0	0	0	1	0	0	1	0	4	2	0	0	**26**
Surface	N 51	0	3	2	5	0	0	0	0	0	0	0	0	0	0	0	0	0	3	1	0	0	**14**
Surface	N 52	1	6	5	6	0	0	1	0	0	1	0	1	1	0	0	1	0	3	0	1	0	**27**
Surface	N 57	0	3	0	1	0	0	0	1	0	2	0	0	0	1	0	2	0	4	0	0	1	**15**
Surface	N 60	2	1	0	4	0	0	0	0	0	1	0	1	0	0	1	0	0	3	1	0	0	**14**
Surface	S 19	1	0	0	2	0	0	0	0	0	0	0	0	0	0	0	1	0	2	0	1	0	**7**
Surface	S 2	4	3	0	2	1	0	1	0	0	0	0	0	0	0	0	1	0	1	0	0	0	**13**
Surface	S 7	0	0	0	0	0	0	0	0	0	0	0	0	0	0	0	0	0	0	0	0	0	**0**
Surface	W 62	0	0	0	0	0	0	0	0	0	0	0	0	0	0	0	0	0	0	0	0	0	**0**
Surface	W 67	2	8	0	4	0	0	0	0	2	0	0	2	2	0	0	0	0	0	0	4	0	**24**
Surface	W 70	0	1	0	0	0	0	0	0	0	1	0	0	0	0	0	0	0	0	2	0	0	**4**
Surface	W 75	0	0	0	0	0	0	0	0	0	0	0	0	0	0	0	0	0	0	0	0	0	**0**
Surface	W 77	0	0	0	0	0	0	0	0	0	0	0	0	0	0	0	0	0	0	0	0	0	**0**
**Surface Total**		**16**	**55**	**9**	**44**	**3**	**3**	**3**	**15**	**11**	**12**	**0**	**5**	**6**	**3**	**3**	**9**	**1**	**58**	**12**	**8**	**2**	**278**
**Grand Total**		**51**	**91**	**15**	**75**	**4**	**5**	**3**	**15**	**32**	**30**	**8**	**19**	**20**	**4**	**8**	**26**	**1**	**128**	**30**	**17**	**3**	**585**

Supplementary Table 9. Starch granule tally per sample: surface and subsurface.

Sample		Conoid	Cylindroid	Globular	Lenticular	Parabolic Prism	Pear Shaped	Prismatic	Reniform	Total
Subsurface	1	3	0	1	0	1	0	0	0	**5**
Subsurface	2	0	5	3	0	1	1	2	0	**12**
Subsurface	3	0	0	0	0	0	0	0	0	**0**
Subsurface	4	0	0	0	0	0	0	0	0	**0**
Subsurface	5	1	0	75	10	0	1	0	0	**87**
Subsurface	6	0	4	11	1	0	2	0	0	**18**
Subsurface	7	2	3	8	0	0	2	1	0	**16**
Subsurface	8	0	1	0	0	2	0	0	0	**3**
Subsurface	9	0	0	0	0	0	0	0	0	**0**
Subsurface	10	0	3	8	0	1	5	0	0	**17**
Subsurface	11	0	2	2	0	0	1	2	0	**7**
Subsurface	12	0	2	2	0	0	0	0	0	**4**
Subsurface	13	0	2	1	0	0	0	0	0	**3**
Subsurface	14	0	0	1	0	1	1	0	0	**3**
Subsurface	15	0	0	0	0	0	0	0	0	**0**
Subsurface	16	0	3	5	0	0	1	3	0	**12**
Subsurface	17	0	1	19	0	0	0	3	0	**23**
Subsurface	18	0	2	3	0	1	1	0	0	**7**
Subsurface	19	0	0	6	0	0	2	1	0	**9**
Subsurface	20	0	0	2	0	1	0	0	0	**3**
**Subsurface Total**		**6**	**28**	**147**	**11**	**8**	**17**	**12**	**0**	**229**
Surface	E 24	0	7	1	1	3	0	1	1	**14**
Surface	E 27	0	6	1	1	3	3	1	0	**15**
Surface	E 31	0	5	7	0	3	3	1	0	**19**
Surface	E 32	0	1	1	0	1	3	2	0	**8**
Surface	E 40	1	1	2	0	0	0	1	0	**5**
Surface	N 47	0	2	3	0	0	0	2	0	**7**
Surface	N 48	0	0	2	0	1	2	2	0	**7**
Surface	N 49	0	1	3	0	0	0	0	0	**4**
Surface	N 51	0	0	2	0	0	0	0	0	**2**
Surface	N 52	0	0	4	2	1	2	1	0	**10**
Surface	N 57	0	0	0	0	1	0	0	0	**1**
Surface	N 60	0	6	12	0	0	2	3	1	**24**
Surface	S 19	0	0	2	0	1	0	1	0	**4**
Surface	S 2	0	5	12	2	4	6	2	0	**31**
Surface	S 7	0	1	5	0	1	0	1	0	**8**
Surface	W 62	0	0	1	0	1	0	1	0	**3**
Surface	W 67	0	1	5	0	0	0	0	0	**6**
Surface	W 70	0	0	3	0	0	0	0	0	**3**
Surface	W 75	0	0	0	0	0	0	0	0	**0**
Surface	W 77	0	2	1	0	0	0	0	0	**3**
**Surface Total**		**1**	**38**	**67**	**6**	**20**	**21**	**19**	**2**	**174**
**Grand Total**		**7**	**66**	**214**	**17**	**28**	**38**	**31**	**2**	**403**

Supplementary Table 10. Tally of diatoms, spores, and spicules.

Sample		Diatom	Palynomorph	Sponge, Spicule	Total
Subsurface	1	0	0	0	0
Subsurface	2	0	2	0	2
Subsurface	3	0	0	0	0
Subsurface	4	0	0	0	0
Subsurface	5	0	0	0	0
Subsurface	6	0	1	0	1
Subsurface	7	2	1	0	3
Subsurface	8	0	2	0	2
Subsurface	9	0	3	0	3
Subsurface	10	0	0	0	0
Subsurface	11	0	0	1	1
Subsurface	12	1	0	0	1
Subsurface	13	3	0	0	3
Subsurface	14	0	0	0	0
Subsurface	15	0	0	0	0
Subsurface	16	0	2	0	2
Subsurface	17	0	1	0	1
Subsurface	18	0	5	0	5
Subsurface	19	0	0	0	0
Subsurface	20	0	0	0	0
**Subsurface Total**		**6**	**17**	**1**	**24**
Surface	E 24	1	1	0	2
Surface	E 27	6	1	1	8
Surface	E 31	8	9	0	17
Surface	E 32	0	0	0	0
Surface	E 40	11	0	0	11
Surface	N 47	0	0	0	0
Surface	N 48	2	1	0	3
Surface	N 49	1	2	0	3
Surface	N 51	4	3	0	7
Surface	N 52	1	0	0	1
Surface	N 57	2	0	0	2
Surface	N 60	18	3	1	22
Surface	S 19	0	2	0	2
Surface	S 2	0	0	1	1
Surface	S 7	0	1	0	1
Surface	W 62	0	0	0	0
Surface	W 67	1	1	0	2
Surface	W 70	0	0	0	0
Surface	W 75	0	0	0	0
Surface	W 77	0	2	0	2
**Surface Total**		**55**	**26**	**3**	**84**
**Grand Total**		**61**	**43**	**4**	**108**

Supplementary Table 11. Statistical classification of residue patterning in surface and subsurface stones.

ID	Side A	Side B	Side C	Side D	Side E	Side F
Subsurface 5	Dispersed (p-value) 0.01	Dispersed 0.01	Dispersed 0.01	–	–	–
Subsurface 7	Dispersed 0.01	Dispersed 0.01	Dispersed 0.01	Dispersed 0.01	Dispersed 0.01	Dispersed 0.01
Subsurface 8	–	Random	Dispersed 0.01	–	–	–
Subsurface 18	–	–	–	–	–	–
Subsurface 26	–	–	–	–	–	–
Subsurface 28	–	–	–	–	–	–
Subsurface 29	–	–	–	Dispersed 0.01	–	–
Subsurface 39	–	Dispersed 0.01	–	–	–	–
Surface E 31	Dispersed 0.01	Dispersed 0.05	Dispersed 0.01	Dispersed 0.01	Dispersed 0.01	Dispersed 0.01
Surface E 39	Random	Dispersed 0.05	Dispersed 0.01	–	–	–
Surface N 42	Clustered 0.01	Dispersed 0.01	Dispersed 0.01	Dispersed 0.01	Dispersed 0.01	–
Surface N 47	Random	Random	Dispersed 0.01	Dispersed 0.01	Dispersed 0.01	–
Surface S 17	Dispersed 0.01	Random	Clustered 0.01	Clustered 0.01	Dispersed 0.01	Dispersed 0.01
Surface W 62	Dispersed 0.01	Random	Dispersed 0.05	–	–	–
Surface W 71	–	–	–	–	–	–
Surface W 77	Dispersed 0.01	Dispersed 0.01	Dispersed 0.01	Dispersed 0.10	–	–

Supplementary Table 12a. Correlation between total stone area and coating area: Surface, subsurface, and experimental blanks.

Normality Test for Surface Assemblage

	Stone Area	Residues Area
N	8	8
Shapiro-Wilk W	0.94	0.53
p(normal)	0.64	2.35E-05
Anderson-Darling A	0.30	1.78
p(normal)	0.50	4.10E-05
p(Monte Carlo)	0.53	0.00
Jarque-Bera JB	0.50	9.02
p(normal)	0.78	0.01
p(Monte Carlo)	0.65	0.00

Kendall's Tau

K-T	p (uncorr.)
0	1

Normality Test for Subsurface Assemblage

	Stone Area	Residues Area
N	8	8
Shapiro-Wilk W	0.91	0.72
p(normal)	0.37	0.00
Anderson-Darling A	0.36	1.06
p(normal)	0.34	0.00
p(Monte Carlo)	0.37	0.00
Jarque-Bera JB	0.84	1.52
p(normal)	0.66	0.47
p(Monte Carlo)	0.30	0.09

Kendall's Tau

K-T	p (uncorr.)
0.32	0.27

Normality Test for Experimental Assemblage (Cycle 1)

	StoneArea	Residues Area
N	9	9
Shapiro-Wilk W	0.90	0.82
p(normal)	0.25	0.03
Anderson-Darling A	0.40	0.82
p(normal)	0.28	0.02
p(Monte Carlo)	0.29	0.02
Jarque-Bera JB	0.80	1.39
p(normal)	0.67	0.50
p(Monte Carlo)	0.37	0.11

Kendall's Tau

K-T	p (uncorr.)
-0.17	0.53

Normality Test for Experimental Assemblage (Cycle 4)

	StoneArea	Residues Area
N	9	9
Shapiro-Wilk W	0.90	0.84
p(normal)	0.25	0.05
Anderson-Darling A	0.40	0.64
p(normal)	0.28	0.06
p(Monte Carlo)	0.31	0.06
Jarque-Bera JB	0.80	2.31
p(normal)	0.67	0.31
p(Monte Carlo)	0.37	0.05

Correlation between Blank and Residue Areas for Experimental (Cycle 4)

RPearson	P (uncorr.)
0.14	0.71
K-T	p (uncorr.)
-0.17	0.53

Supplementary Table 12b. Correlation between facet/side area and coating extent
Surface, subsurface, and experimental blanks.

Normality Test for Surface Assemblage

	Side Area	Residues Area
N	42	42
Shapiro-Wilk W	0.96	0.20
p(normal)	0.22	8.23E-14
Anderson-Darling A	0.41	13.74
p(normal)	0.33	2.63E-33
p(Monte Carlo)	0.34	0.00
Jarque-Bera JB	2.45	2397
p(normal)	0.29	0
p(Monte Carlo)	0.15	0.00

Kendall's Tau Correlation between Side and Residue Areas at Surface Assemblage

K-T	p (uncorr.)
-0.02	0.87

Normality Test for Subsurface Assemblage

	Side Area	Residues Area
N	31	31
Shapiro-Wilk W	0.91	0.58
p(normal)	0.01	3.03E-08
Anderson-Darling A	0.80	5.87
p(normal)	0.03	8.12E-15
p(Monte Carlo)	0.03	0.00
Jarque-Bera JB	4.25	35
p(normal)	0.12	2.52E-08
p(Monte Carlo)	0.05	0.00

Kendall's Tau Correlation between Side and Residue Areas at Surface Assemblage

K-T	p (uncorr.)
0.19	0.13

Normality Test for Experimental Assemblage at Cycle 1

	Side Area	Residues Area
N	53	53
Shapiro-Wilk W	0.95	0.72
p(normal)	0.04	9.24E-09
Anderson-Darling A	0.62	5.68
p(normal)	0.10	3.48E-14
p(Monte Carlo)	0.10	0.00
Jarque-Bera JB	2.43	33.77
p(normal)	0.30	4.65E-08
p(Monte Carlo)	0.17	0.00

Kendall's Tau Correlation between Side and Residue Areas at Surface versus Experimental (Cycle 1)

K-T	p (uncorr.)
-0.24	0.09

Normality Test for Experimental Cycle 4

	Side Area	Residues Area
N	53	53
Shapiro-Wilk W	0.95	0.62
p(normal)	0.04	2.08E-10
Anderson-Darling A	0.62	7.26
p(normal)	0.10	5.40E-18
p(Monte Carlo)	0.10	0.00
Jarque-Bera JB	2.43	184.50
p(normal)	0.30	8.44E-41
p(Monte Carlo)	0.17	0.00

Kendall's Tau Correlation between Side and Residue Areas at Experimental Cycle 4

K-T	p (uncorr.)
0.02	0.07

Supplementary Table 12c. Correlation between stone total area and microbotanical remains Surface, subsurface, and experimental blanks.

Contingency Table: GIS Surface-Microbotany

	Stone Area	Starch Total	Phytolith Total	Other Total
Surface 31	271.8	19	47	17
Surface 47	147.3	7	6	0
Surface 62	214.8	3	0	0
Surface 77	219.6	3	0	2

Normality Test

	Stone Area	Starch Total	Phytolith Total	Other Total
N	4	4	4	3
Shapiro-Wilk W	0.94	0.75	0.90	0.96
p(normal)	0.64	0.04	0.42	0.64
Anderson-Darling A	0.24	0.58	0.30	0.23
p(normal)	0.53	0.04	0.37	0.49
p(Monte Carlo)	0.67	0.04	0.47	0.63
Jarque-Bera JB	0.44	0.88	0.52	0.35
p(normal)	0.80	0.64	0.77	0.84
p(Monte Carlo)	0.60	0.04	0.38	0.63

Kendall's Tau

	Stone Area	Starch Total	Phytolith Total	Other Total
Stone Area	-	0.42	0.08	1
Starch Total	0.40	-	0.75	1
Phytolith Total	0.80	0.20	-	0.33
Other Total	0.50	0.50	1	-

Contingency Table: GIS Subsurface-Microbotany

	Stone Area	Starch Total	Phytolith Total	Other Total
Subsurface 5	68.5	87	8	0
Subsurface 7	218.3	16	38	3
Subsurface 8	41.7	3	3	2
Subsurface 18	127.5	7	53	5

Normality Test

	Stone Area	Starch Total	Phytolith Total	Other Total
N	4	4	4	3
Shapiro-Wilk W	0.94	0.75	0.90	0.96
p(normal)	0.64	0.04	0.42	0.64
Anderson-Darling A	0.24	0.58	0.30	0.23
p(normal)	0.53	0.04	0.37	0.49
p(Monte Carlo)	0.67	0.04	0.47	0.62
Jarque-Bera JB	0.44	0.88	0.52	0.35
p(normal)	0.80	0.64	0.77	0.84
p(Monte Carlo)	0.61	0.04	0.38	0.63

Kendall's Tau Correlation: Subsurface Stone Area-Microbotany Content

	Stone Area	Starch Total	Phytolith Total	Other Total
Stone Area	-	0.50	0.17	0.60
Starch Total	0.33	-	1	0.60
Phytolith Total	0.67	0	-	0.12
Other Total	0.33	0.33	1	-

Supplementary Table 13. Surface stone metrics per facet, along with residue area and percentage.

	Side cm^2	Residue cm^2	Residue %
Surface N 1-1 C1 Side A	54.9	0.4	0.7
Surface N 1-1 C1 Side B	64.8	0.01	0
Surface N 1-1 C1 Side C	56.0	0.02	0
Surface N 1-1 C1 Side D	60.0	0.04	0.1
Surface N 1-1 C1 Side E	43.2	0.9	2.0
Surface N 1-1 C1 Side F	59.6	0.07	0.1
Surface N 1-1 C4 Side A	54.9	0.4	0.7
Surface N 1-1 C4 Side B	64.8	0.01	0
Surface N 1-1 C4 Side C	56.0	0.02	0
Surface N 1-1 C4 Side D	60.0	0.04	0.1
Surface N 1-1 C4 Side E	43.2	0.9	2.0
Surface N 1-1 C4 Side F	59.6	0.02	0
Surface N 1-2 C1 Side A	33.0	9.4	28.4
Surface N 1-2 C1 Side B	49.4	14.6	29.6
Surface N 1-2 C1 Side C	39.8	4.4	11.2
Surface N 1-2 C1 Side D	26.8	4.9	18.3
Surface N 1-2 C1 Side E	11.6	0.7	5.8
Surface N 1-2 C4 Side A	33.0	3.3	9.8
Surface N 1-2 C4 Side B	49.4	3.9	7.9
Surface N 1-2 C4 Side C	39.8	2.4	6.1
Surface N 1-2 C4 Side D	26.8	4.5	16.8
Surface N 1-2 C4 Side E	11.6	0.5	3.9
Surface N 2-1 C1 Side A	85.8	4.8	5.5
Surface N 2-1 C1 Side B	82.3	0.0	0
Surface N 2-1 C1 Side C	106.0	0.7	0.7
Surface N 2-1 C1 Side D	64.8	1.6	2.5
Surface N 2-1 C1 Side E	116.0	0.6	0.5
Surface N 2-1 C1 Side F	57.4	0	0
Surface N 2-1 C4 Side A	85.8	1.9	2.2
Surface N 2-1 C4 Side B	82.3	0.0	0
Surface N 2-1 C4 Side C	106.0	0.7	0.7
Surface N 2-1 C4 Side D	64.8	1.2	1.9
Surface N 2-1 C4 Side E	116.0	0.6	0.5
Surface N 2-1 C4 Side F	57.4	0	0
Surface N 2-2 C1 Side A	85.4	3.6	4.2
Surface N 2-2 C1 Side B	58.3	0.5	0.8
Surface N 2-2 C1 Side C	53.3	0.6	1
Surface N 2-2 C1 Side D	53.7	0.07	0.1
Surface N 2-2 C1 Side E	66.3	0.2	0.3
Surface N 2-2 C1 Side F	54.0	3.4	6.2
Surface N 2-2 C4 Side A	85.4	0.06	0.1
Surface N 2-2 C4 Side B	58.3	0.05	0.1
Surface N 2-2 C4 Side C	53.3	0.03	0.1
Surface N 2-2 C4 Side D	53.7	0.04	0.1
Surface N 2-2 C4 Side E	66.3	0.2	0.2
Surface N 2-2 C4 Side F	54.0	0.5	0.9
Surface N 9-1 C1 Side A	109.7	6.8	6.2
Surface N 9-1 C1 Side B	100.0	0.6	0.6
Surface N 9-1 C1 Side C	34.5	2.2	6.3
Surface N 9-1 C1 Side D	41.5	0.4	0.9
Surface N 9-1 C1 Side E	59.8	13.2	22.0
Surface N 9-1 C4 Side A	109.7	0.03	0
Surface N 9-1 C4 Side B	100.0	0.07	0.1
Surface N 9-1 C4 Side C	34.5	2.1	6.0
Surface N 9-1 C4 Side D	41.5	0.4	0.9
Surface N 9-1 C4 Side E	59.8	4.4	7.4
Surface N 12-1 C1 Side A	76.1	1.2	1.6
Surface N 12-1 C1 Side B	88.9	7.4	8.3
Surface N 12-1 C1 Side C	75.9	4.3	5.7
Surface N 12-1 C1 Side D	98.6	3.5	3.5
Surface N 12-1 C1 Side E	78.0	11.6	14.8
Surface N 12-1 C1 Side F	68.8	11.6	16.8
Surface N 12-1 C4 Side A	76.1	0.2	0.2
Surface N 12-1 C4 Side B	88.9	2.2	2.5
Surface N 12-1 C4 Side C	75.9	1.1	1.4
Surface N 12-1 C4 Side D	98.6	3.2	3.3
Surface N 12-1 C4 Side E	78.0	5.2	6.7
Surface N 12-1 C4 Side F	68.8	10.7	15.6
Surface N 13-1 C1 Side A	80.7	0.03	0
Surface N 13-1 C1 Side B	104.9	0	0
Surface N 13-1 C1 Side C	64.0	1.1	1.7
Surface N 13-1 C1 Side D	117.5	2.1	1.7
Surface N 13-1 C1 Side E	87.3	0.01	0
Surface N 13-1 C1 Side F	72.1	2.9	4.0
Surface N 13-1 C4 Side A	80.7	0	0
Surface N 13-1 C4 Side B	104.9	0	0
Surface N 13-1 C4 Side C	64.0	0.5	0.8
Surface N 13-1 C4 Side D	117.5	0.04	0
Surface N 13-1 C4 Side E	87.3	0	0
Surface N 13-1 C4 Side F	72.1	2.8	3.9
Surface N 13-2 C1 Side A	20.2	0	0
Surface N 13-2 C1 Side B	30.7	0	0
Surface N 13-2 C1 Side C	27.3	0	0
Surface N 13-2 C1 Side D	20.2	9.5	47.1
Surface N 13-2 C1 Side E	21.4	0	0
Surface N 13-2 C1 Side F	17.0	0	0
Surface N 13-2 C1 Side G	25.9	0	0
Surface N 13-2 C4 Side A	20.2	0	0
Surface N 13-2 C4 Side B	30.7	0	0
Surface N 13-2 C4 Side C	27.3	0	0
Surface N 13-2 C4 Side D	20.2	6.5	32.2
Surface N 13-2 C4 Side E	21.4	0	0
Surface N 13-2 C4 Side F	17.0	0	0
Surface N 13-2 C4 Side G	25.9	0	0
Surface N 13-3 C1 Side A	20.6	4.8	23.1
Surface N 13-3 C1 Side B	13.1	0.01	0.1
Surface N 13-3 C1 Side C	19.2	2.2	11.3
Surface N 13-3 C1 Side D	17.1	0.06	0.4
Surface N 13-3 C1 Side E	12.3	0.5	4.1
Surface N 13-3 C1 Side F	19.1	0.5	2.8
Surface N 13-3 PC1 Side A	20.6	0.6	2.9
Surface N 13-3 PC1 Side B	13.1	0.01	0.1
Surface N 13-3 PC1 Side C	19.2	0.4	2.0
Surface N 13-3 PC1 Side D	17.1	0	0
Surface N 13-3 PC1 Side E	12.3	0.02	0.2
Surface N 13-3 PC1 Side F	19.1	0.09	0.5
Surface N 13-3 C2 Side A	20.6	0.2	1.1
Surface N 13-3 C2 Side B	13.1	0	0
Surface N 13-3 C2 Side C	19.2	0.11	0.6
Surface N 13-3 C2 Side D	17.1	0	0
Surface N 13-3 C2 Side E	12.3	0.01	0.1
Surface N 13-3 C2 Side F	19.1	0.05	0.3
Surface N 13-3 C3 Side A	20.6	0.13	0.6
Surface N 13-2 C3 Side B	13.1	0	0
Surface N 13-2 C3 Side C	19.2	0.01	0.1
Surface N 13-2 C3 Side D	17.1	0	0
Surface N 13-2 C3 Side E	12.3	0.01	0.1
Surface N 13-2 C3 Side F	19.1	0.03	0.2
Surface N 13-2 C4 Side A	20.6	0.06	0.3
Surface N 13-2 C4 Side B	13.1	0	0
Surface N 13-2 C4 Side C	19.2	0	0
Surface N 13-2 C4 Side D	17.1	0	0
Surface N 13-2 C4 Side E	12.3	0.01	0.1
Surface N13-2 C4 Side F	19.1	0.01	0.1

Supplementary Table 14. Environmental starch cross contamination during experimental pounding: granule types per tool.

Tool	Action	Material	Hazelnut	Cactus	Potato	Wheat	Maize	Other
Spheroid	Cracking	Hazelnut	0	8	11	5	0	6
Anvil	Cracking	Hazelnut	12	0	1	0	0	0
Spheroid	Smashing	Cactus	7	1	0	6	4	5
Anvil	Smashing	Cactus	0	0	0	0	0	0
Spheroid	Pounding	Meat	3	0	2	0	0	1
Anvil	Peeling	Potato	3	0	6	0	1	0
Spheroid	Cracking	Bone	0	0	1	0	0	0
Anvil	Cracking	Bone	1	7	1	0	0	15
**Total**			**26**	**16**	**22**	**11**	**5**	**27**

Supplementary Table 15. Comparison of microbotanical remains on stone blanks and freestanding soils.

	Surface	Subsurface	Experimental
Starch	174	229	129
Phytolith	278	307	40
Other	84	24	767

Normality Tests MCB Surface versus subsurface versus experimental stone

	Surface	Subsurface	Experimental
N	3	3	3
Shapiro-Wilk W	1.00	0.94	0.84
p(normal)	0.92	0.52	0.21
Anderson-Darling A	0.19	0.26	0.38
p(normal)	0.62	0.38	0.15
p(Monte Carlo)	0.92	0.52	0.21
Lilliefors L	0.19	0.28	0.34
p(normal)	1.14	0.49	0.19
p(Monte Carlo)	0.92	0.51	0.21
Jarque-Bera JB	0.29	0.40	0.50
p(normal)	0.87	0.82	0.78
p(Monte Carlo)	0.92	0.51	0.22

Chi^2 Microbotany

Rows, columns:	3, 3	df:	4
Chi^2:	1123.40	p (no assoc.):	6.40E-242
Monte Carlo p :	0.00		
Fisher´s exact			
Not available			
Other statistics			
Cramer´s V :	0.53	Contingency C :	0.60

ANOVA Test: Microbotany Population Distribution

	Sum of sqrs	df	Mean square	F	p (same)
Between groups:	33550.20	2	16775.10	0.27	0.77
Within groups:	376081	6	62680.20	ıutation p (n=99999)	
Total:	409632	8	0.90		
Components of variance (only for random effects):					
Var(group):	-15301.70	Var(error):	62680.20	ICC:	-0.32
omega2:	0				
Levene´s test for homogeneity of variance, from means			p (same):	0.04	
Levene´s test, from medians			p (same):	0.57	
Welch F test in the case of unequal variances: F=0,1343, df=3,482, p=0,8787					

Tukeys Pairwise Test: Microbotany

	Surface	Subsurface	Experimental
Surface	-	1.00	0.80
Subsurface	0.06	-	0.82
Experimental	0.92	0.87	-

Contingency Table of MBT from experimental stones versus Bootstrapped samples representing microbotanicals published from topsoils

	Experimental	Soil (Bootstrapped n=8)
Starch	129	54
Phytolith	40	2441

Normality Tests

	Experimental	Soil (Bootstrapped n=8)
N	2	2
Shapiro-Wilk W	1	1
p(normal)	1	1
Anderson-Darling A	0.25	0.25
p(normal)	0.23	0.23
p(Monte Carlo)	1	1
Lilliefors L	0.26	0.26
p(normal)	0.78	0.78
p(Monte Carlo)	1	1
Jarque-Bera JB	0.33	0.33
p(normal)	0.85	0.85
p(Monte Carlo)	0.89	0.90

Chi^2 of both distributions

Chi squared			
Rows, columns:	2, 2	df:	1
Chi^2	1360.90	p (no assoc.):	6.50E-298
Monte Carlo p :	0.00		
Fisher´s exact			
p (no assoc.):	6.95E-138		
Other statistics			
Cramer´s V :	0.71	Contingency C :	0.58

Supplementary Table 16. Degree of association and population distribution for microbotanical remains on stone and freestanding soils.

Contingency Table: Microbotany

	Subsurface	Surface
Starch	229	174
Phytolith	307	278
Other	24	84

Chi^2 Microbotany: Surface and Subsurface

Rows, columSurface:	3, 2	df:	2
Chi^2	41.77	p:	8.50E-10
Monte Carlo p :	0.00		
Fisher´s exact			
p (no assoc.):	3.60E-10		
Other statistics			
Cramer´s V :	0.20	Contingency C :	0.19

K-S to Test Equal Distribution: Surface versus Subsurface

Surface		Subsurface	
N:	3	N:	3
D :	0.33	p (same dist.):	0.98
Monte Carlo permutation: p (same dist.):		1	

Starch by Morphotype: Surface and Subsurface

	Subsurface	Surface
Conoid	6	1
Cylindroid	28	38
Globular	147	67
Lenticular	11	6
Parabolic Prism	8	20
Pear shaped	17	21
Prismatic	12	19
Reniform	0	2

Chi^2 of Starch by Morphotype: Surface and Subsurface

Rows, columSurface:	8, 2	df:	7
Chi^2	38.83	p:	2.11E-06
Monte Carlo p :	0.00		
Fisher´s exact			
Not available			
Other statistics			
Cramer´s V :	0.31	Contingency C :	0.30

K-S Test for Equal Distribution: Starch Morphotypes

Subsurface		Surface	
N:	8	N:	8
D :	0.38	p (same dist.):	0.52
Monte Carlo permutation: p (same dist.):		0.67	

Phytolith Morphotypes: Surface and Subsurface

	Subsurface	Surface
Bilobate	35	16
Blocky	36	55
Bulliform	6	9
Cylindroid	31	44
Epidermis, leaf, grass	1	3
Globular granulate	2	3
Globular psilate	0	3
Globular ridged	0	15
Globular ridged (centric ca	21	11
Hair	18	12
Hemisphere	8	0
Rondel	14	5
Saddle	14	6
Saddle long	1	3
Sclereid	5	3
Scutiform	17	9
Shield	0	1
Tabular	70	58
Tabular oblong	18	12
Tower	9	8
Vessel	1	2

Chi^2 of Phytolith by Morphotype: Surface and Subsurface

Rows, columSurface:	21, 2	df:	20
Chi^2	59.27	p:	9.22E-06
Monte Carlo p :	0.00		
Fisher´s exact			
Not available			
Other statistics			
Cramer´s V :	0.32	Contingency C :	0.30

K-S Test for Equal Distribution: Phytolith Morphotypes

Subsurface		Surface	
N:	21	N:	21
D :	0.24	p (same dist.):	0.53
Monte Carlo permutation: p (same dist.):		0.55	

Other: Surface and Subsurface

	Subsurface	Surface
Diatom	6	55
Palynomorph	17	26
Sponge, spicule	1	3

Chi^2 of Other by Morphotypes: Surface and Subsurface

Rows, columSurface:	3, 2	df:	2
Chi^2	12.89	p:	0.00
Monte Carlo p :	0.00		
Fisher´s exact			
p (no assoc.):	0.00		
Other statistics			
Cramer´s V :	0.35	Contingency C :	0.33

K-S Test for Equal Distribution: Others

Subsurface		Surface	
N:	3	N:	3
D :	0.67	p (same dist.):	0.32
Monte Carlo permutation: p (same dist.):		0.40	

Supplementary Method 1. Solid-state NMR spectroscopy.

^{27}Al and ^{29}Si solid state NMR experiments were performed using a Bruker AVANCE III HD spectrometer operating at 130.32 and 99.36 MHz respectively (magnetic field 11.75 T, ^{1}H frequency at 500.13MHz), with a 4 mm DOTY CP-MAS probe. For ^{27}Al Magic Angle Spinning (MAS) NMR experiments, the samples were spinning at a MAS rate of 13 kHz. A 90° pulse of 1.2 μs was used for the ^{27}Al channel, with no ^{1}H decoupling. For all the samples, 1024 scans were accumulated, with a recycle delay of 1 s. Chemical shifts are referenced to 1 M $Al(NO_3)_3$ aqueous solution at 0ppm. Both ^{29}Si MAS and CP/MAS (Cross Polarization) experiments were carried out at a spinning speed of 7.5 kHz, with ^{1}H decoupling. For ^{29}Si MAS experiments, 2300 – 4900 scans were accumulated for different samples, with a 45° pulse and a 10 s recycle delay. For ^{29}Si CP/MAS experiments, 2200 – 4096 scans were accumulated for different samples, with a recycle delay of 2 s and a contact time of 5.0 ms. ^{29}Si Chemical shifts are referenced to a Na-DSS (4,4-dimethyl-4-silapentane-1-sulfonic acid sodium salt) at 0 ppm.

Supplementary Method 2. Powder X-Ray Diffraction.

The powder X-Ray Diffraction (P-XRD) experiments were performed on a Rigaku Ultima IV X-Ray Diffractometer, equipped with a Cu source (1.54056 Å), a CBO optical, and a Scintillation Counter detector. The diffractometer was operated at 40 kV and 44 mA. The measurements were carried out on the Multipurpose Attachment, with parafocusing mode. A K_β filter (Ni foils) were placed at the receiving end. The samples were loaded onto glass sample holder without any treatments. 2θ was scanned from 5° to 90°, with a scan rate of 2° per minute (step size: 0.02°), for all the samples.

Supplementary Method 3. Raman Spectroscopy.

Raman spectroscopy measurements were carried out on a Renishaw InVia Reflex Raman microscope using a solid state diode laser (Renishaw Inc.) operating at 785 nm and a 1200 line/mm grating. The microscope was focussed onto the sample using a Leica 50X NPLAN (NA = 0.75) objective, and the backscattered Raman signals were collected with a Peltier cooled CCD detector. Measurements were collected using extended scan using a 10 s detector time. The laser power was 65-130 mW measured at the sample (archeological samples). The instrument was calibrated using an internal Si(110) sample, which was measured at 520 cm^{-1}. The archeological samples were mounted on a Au-coated Si substrate by suspending a small amount of archeological sample in methanol, and then dispersing the solution onto the substrate. For each sample, the Raman spectrum for 4-6 different mineral samples were acquired.

Supplementary Method 4. Microscopy workflow (IPHES-CERCA).

The initial step in residue analysis was conducted at low magnification, using a stereomicroscope (Euromex DZ.1105), with a magnification range of 8×-80× (0.8×-8× zoom, 10× oculars), and equipped with a 20MP 1" Scemex camera (HFOV 16.6 mm - 1.67 mm).

Next, we used 3D DM (Hirox KH-8700). This microscope is equipped with a dual illumination revolver zoom lens (MXG-5000REZ) which together with the high intensity LED light source allows for the observation of samples with a 5700 K color temperature. This lens consists of a triple objective turret with a different zoom range per objective, allowing for magnifications ranging from 35 to 5000× (HFOV 8.6 mm–60 µ). The microscope is equipped with a high-sensitivity compact CCD camera that enabled the capture of 24 frames per second at high-quality resolution (1600 × 1200 pixels). The dual illumination system allows for the observation of sample topography with ring and coaxial light. The former is suitable for the observation of topographical irregularities with a uniform even illumination at low magnifications, while coaxial light serves to highlight the topography of flat surfaces at high magnifications. Ring and coaxial lights were used individually and even combined, according to the observation conditions required by the samples. A particularly remarkable advance of this microscope was its integrated stacking and stitching technology, capable of performing real-time 2D and 3D tilings and generating wide field of view images of large surface areas which, in turn, enable quantifiable 3D models.

We also used an environmental SEM (ESEM FEI Quanta 600 model) with an energy dispersive X-ray spectrometer (EDX-EXL II System Analytical Oxford) for microanalysis, equipped with an INCA software (v 4.01) from Oxford Instruments for digital image acquisition. Most observations were carried between nominal magnifications ranging from 60× to 10,000× (HFOV 6.9 mm to 41.4 µ, 24" display), although higher magnifications were sporadically used (50,000x, HFOV 8.29 µ). Observation was entirely done in low vacuum mode (LV), so the coating of the samples with conductive materials (gold or carbon) was not required. Large field (LFD) and back-scattered electron detectors (DualBSD) were used in combination to observe both residues and use-wear features.

In addition, we utilized a FESEM-FIB Scios 2 equipment. This is a dual beam scanning electron microscope. The field emission electron source can obtain high resolution images (resolution of 1 nm). The galium focus ion beam enables 3D characterization of all kinds of materials, including magnetic and isolating materials. The microscope has several detectors: in-column for the detection of different energy electrons, STEM to work in transmission mode and an energy dispersive X-ray detector

Supplementary Text. Petrographic descriptions.

Surface 039 (Raw Material Group 1)
<u>Quartzite</u>

Macroscopic traits: very coarse-grained white (N9 White) quartzite, translucent, with no zonation. Sub-primary cortical surfaces with aligned muscovite crystals.

Microscopic traits: Inequigranular crystals with frequent inter - intracrystalline fissures (max width: 327.27 µm).

Mineralogy: Macrocrystalline quartz (95-98%).as main texture (800 µm -1.5mm), but also fibrous quartz with fine hematite upholstering the walls of voids (<2%) (300-750), as recrystallization structures (100-150µm). Nonaligned muscovite (2-5%). 500µm-1.5mm as interstitial crystals.

Surface 06 (Raw Material Group 2)
<u>Quartzite</u>

Macroscopic traits: Coarse-grained quartzite (5GY 8/1 light greenish gray - 10GY 7/2 pale yellowish green). Opaque but edge-translucent. Subprimary cortical surfaces, with yellowish patina, and muscovite. Abundant ferric oxides and fissures.

Microscopic traits: Inequigranular crystals with frequent inter-intra-crystalline fissures (20-50 µm width). Surficial areas present coating/patina (70-121.42µm depth), suggesting weathering and recrystallization processes.

Mineralogy: Macrocrystalline quartz (98%) as main texture (500 µm -1mm), with inter-crystalline fine hematite. Nonaligned muscovite (1-2%) (100µm-1mm), crystals and rare fuchsite (<1%) as inclusions and interstitial. Voids (<670µm) (<1%) are not upholstered.

Surface 042 (Raw Material Group 3)
<u>Quartzite</u>

Macroscopic traits: Very coarse-grained quartzite (10 R 8/2 grayish orange pink - 5YR 7/2 grayish orange pink), opaque. Sub-primary cortical surfaces with orange-pinkish patina (5YR 8/4 Moderate Orange Pink). Presence of nonaligned muscovite, with abundant ferric oxides and diagenetic fissures.

Microscopic traits: Inequigranular crystal with frequent inter-intra-crystalline fissures (50-300µm width). Surficial areas show a coating/patina (80-160µm depth) covering fissures andinter-crystalline crevices, penetrating <850 µm.

Mineralogy: quartz (95-98%). Macrocrystalline quartz (98%) as main texture (700 µm - 1mm). Nonaligned muscovite (2-5%) (200 µm – 2 mm) as interstitial crystals, and massive and fine hematite (80-100µm) as inter-crystalline inclusions are also described. Voids (770µm; <1%) are not upholstered.
