# Supplementary material for: Microbotanical residues for the study of early hominin tools
Source: Sci Rep. 2022 Feb 22;12:2951. doi: 10.1038/s41598-022-06959-1 (PMC8863820; doi:10.1038/s41598-022-06959-1)
Supplement: Supplementary file 8 — Supplementary Legends. [file 41598_2022_6959_MOESM8_ESM.pdf]

Supplementary video captions

Supplementary video 1: Bone cracking. Recording of the experimental process and technical actions utilized in this work to replicate the impact of bone cracking on residue analysis.

Supplementary video 2: Cactus plant pounding. Recording of the experimental process and technical actions utilized in this work to replicate the impact of cactus smashing on residue analysis.

Supplementary video 3: Nut cracking. Recording of the experimental process and technical actions utilized in this work to replicate the impact of nut cracking smashing on residue analysis.

Supplementary video 4: Wood scraping. Recording of the experimental process and technical actions utilized in this work to replicate the impact of wood scraping on residue analysis.

Supplementary video 5: Tuber peeling. Recording of the experimental process and technical actions utilized in this work to replicate the impact of tuber peeling on residue analysis.

Supplementary video 6: Meat pounding. Recording of the experimental process and technical actions utilized in this work to replicate the impact of meat pounding on residue analysis.
